# Asymmetric assembly of high-value α-functionalized organic acids using a biocatalytic chiral-group-resetting process

**DOI:** 10.1038/s41467-018-06241-x

**Published:** 2018-09-19

**Authors:** Wei Song, Jin-Hui Wang, Jing Wu, Jia Liu, Xiu-Lai Chen, Li-Ming Liu

**Affiliations:** 1 State Key Laboratory of Food Science and Technology, Jiangnan University, Wuxi, 214122 China; 2 Key Laboratory of Industrial Biotechnology, Ministry of Education, Jiangnan University, Wuxi, 214122 China; 3 School of Pharmaceutical Sciences, Jiangnan University, Wuxi, 214122 China; 4 National Engineering Laboratory for Cereal Fermentation Technology, Jiangnan University, Wuxi, 214122 China

## Abstract

The preparation of α-functionalized organic acids can be greatly simplified by adopting a protocol involving the catalytic assembly of achiral building blocks. However, the enzymatic assembly of small amino acids and aldehydes to form numerous α-functionalized organic acids is highly desired and remains a significant challenge. Herein, we report an artificially designed chiral-group-resetting biocatalytic process, which uses simple achiral glycine and aldehydes to synthesize stereodefined α-functionalized organic acids. This cascade biocatalysis comprises a basic module and three different extender modules and operates in a modular assembly manner. The engineered *Escherichia coli* catalysts, which contained different module(s), provide access to α-keto acids, α-hydroxy acids, and α-amino acids with excellent conversion and enantioselectivities. Therefore, this biocatalytic process provides an attractive strategy for the conversion of low-cost achiral starting materials to high-value α-functionalized organic acids.

## Abstract (web summary)

Alpha-functionalized organic acids are building blocks of many bioactive compounds. Here, the authors developed a toolbox-like, modular set of enzymes that reset chiral groups, turning achiral glycine and simple aldehydes into stereodefined α-keto acids, α-hydroxy acids, and α-amino acids.

## Introduction

The preparation of multifunctional chiral small molecules such as unnatural α-amino acids and α-hydroxy acids has attracted extensive attention from organic chemists because they play prominent roles in chemical synthesis, cosmetics, and pharmaceutical manufacturing^1–5^. Therefore, a few elegant chemical and enzymatic synthesis methods have been elaborated to provide a sufficient number of chemicals to meet market demand^6–9^. Among them, one particularly attractive synthesis route is the C–H functionalization-mediated asymmetric assembly of small building blocks^10,11^.

However, modern chemical C–H functionalization methods heavily rely on precious and unsustainable transition metals (e.g., Pd, Rh, Ru, and Ir) and protected substrates^12–14^. Hence, some researchers have turned to biocatalytic processes using some key enzymes such as threonine aldolases (TAs)^9,15–17^, which could catalyze the aldol assembly of unprotected small amino acids (principally glycine) and aldehydes to produce small organic acids, providing a dramatically simplified method. Another representative example is pyruvate aldolase, which uses alanine and formaldehyde as starting materials for the biosynthesis of (*S*)- and (*R*)-2-amino-4-hydroxybutanoic acid^9^. Unfortunately, these methods only provide a limited range of products such as β-hydroxy-α-amino acids and L/D-homoserine^9,18,19^. Therefore, the assembly of small amino acids and aldehydes to form numerous α-keto acids and α-hydroxy acids is highly desired and remains a significant challenge. Fortunately, artificially designed multienzyme cascades, which offer a alternative to related chemical methods and protein engineering^3,11,17,20^, provide a useful tool for overcoming some challenging reactions^21–26^, such as the oxy- and amino-functionalization of alkenes^21,24,27,28^.

Here, we report an artificially designed chiral-group-resetting cascade biocatalysis protocol, which could transform unprotected achiral building blocks into prochiral α-keto acids and then to chiral α-hydroxy acids and α-amino acids. The platform operates in a modular assembly manner, enabling the tunable and predictable operation of cascade biocatalysis and retaining a high product diversity (including aliphatic, aromatic, heteroaromatic, and heterocyclic products) and modularity.

## Results

### Designing enzyme cascades for chiral group resetting

**Fig. 1:**
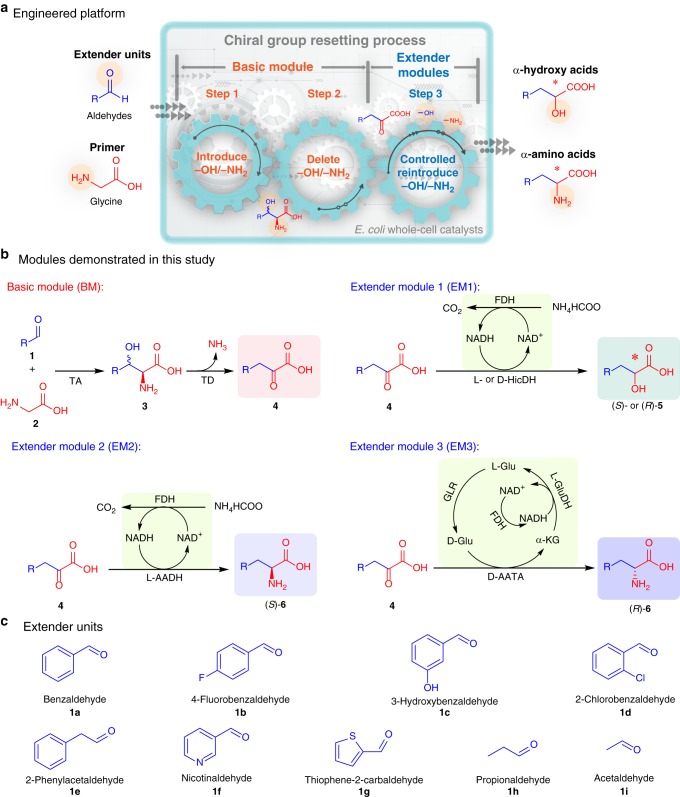
The synthesis of α-functionalized organic acids by proposed modular cascade biocatalysis platform. **a** The engineered modular cascade biocatalysis platform. **b** Four general basic enzyme modules. Basic module: threonine aldolase (TA) and threonine deaminase (TD) for condensation-deamination of aldehydes and glycine to α-keto acids; extender module 1: hydroxyisocaproate dehydrogenase (HicDH) and formate dehydrogenase (FDH) for reduction of α-keto acids to α-hydroxy acid; extender module 2: ʟ-amino acid dehydrogenase (ʟ-AAD) and FDH for reduction of α-keto acids to ʟ-α-amino acids; extender module 3: D-amino acid transaminase (D-AATA), FDH, glutamate racemase (GLR), and glutamate dehydrogenase (GluDH) for transamination of α-keto acids to D-α-amino acids. Functionalized groups (α-position) and extender units (from aldehydes) are colored in red and blue, respectively. **c** Nine different extender units, including aromatic (**1a**–**e**), heteroaromatic (**1f**), heterocyclic (**1g**), and aliphatic (**1h**–**i**) aldehydes

**Fig. 2:**
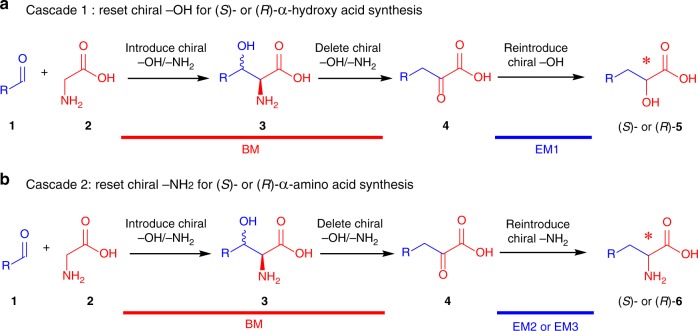
The artificially designed chiral-group-resetting cascades. **a** Cascade 1: reset chiral –OH for stereodefined α-hydroxy acid synthesis via cascade BM with EM1. **b** Cascade 2: reset chiral –NH_2_ for stereodefined α-amino acid synthesis via cascade BM with EM2 or EM3

The designed modular cascade catalysis platform has two parts (Fig. 1a): (i) a basic module (BM), in which achiral aldehyde and glycine are assembled to prochiral α-keto acid (including the introduction and deletion of chiral –OH/–NH_2_ groups), and (ii) extender modules (EMs), in which α-keto acid is converted into α-hydroxy/amino acid by reintroducing chiral –OH/–NH_2_ groups (Fig. 1b). When a BM is coupled with EMs, for chiral –OH/–NH_2_ groups, the whole process is presented as introduction–deletion–reintroduction, which can be seen as an –OH/–NH_2_-group resetting process (Fig. 2a, b). Using easily available glycine as a functionalized primer and nine aldehydes as extender units (Fig. 1c), nine prochiral α-keto acids, 18 chiral α-hydroxy acids, and 18 chiral α-amino acids (Supplementary Fig. 1) were obtained.

The BM starts with the TA-mediated asymmetric assembly (C–C bond formation while introducing chiral –OH/–NH_2_ groups) of the achiral functionalized primer glycine (**2**) with aldehyde extender units (**1**) to produce β-hydroxy-α-amino acids (**3**) (Fig. 1b). The subsequent threonine deaminase (TD)-catalyzed atypical deamination (PLP-dependent α,β-elimination reaction^29^, Supplementary Fig. 2) led to the elimination of the chiral group (C_α_-NH_2_ and C_β_-OH) of **3** while producing prochiral α-keto acids (**4**).

On the basis of the BM, EMs were designed for reintroducing chiral –OH/–NH_2_ groups (Fig. 1b). Extender module 1 (EM1) was designed for the *S*- or *R*-selective reduction of **4** to (*S*)- or (*R*)-hydroxy acids [(*S*)- or (*R*)-**5**] catalyzed by L- or D-hydroxyisocaproate dehydrogenase (ʟ- or D-HicDH), coupled with NADH recycling by formate dehydrogenase (FDH). Extender module 2 (EM2) employed ʟ-amino acid dehydrogenase (ʟ-AADH) for converting **4** into (*S*)-α-amino acids [(*S*)-**6**] and FDH for NADH recycling. Since D-AADHs are not ubiquitous in nature, as opposed to ʟ-selective AADHs, and natural D-AADHs are membrane-bound enzymes dependent on NADPH, which are much more expensive than NADH^30^, we chose D-amino acid transaminase (D-AATA) for extender module 3 (EM3) for transforming **4** into (*R*)-α-amino acids [(*R*)-**6**] and glutamate dehydrogenase (GluDH), FDH, and glutamate racemase (GLR) for amino donor recycling.

The enzymes used in all of these modules were selected on the basis of literature reports on the abilities of specific enzymes and organisms to function in the presence of the required intermediates. For the further assembly of multiple modules and the optimization of enzyme expression in one *E. coli* strain, every module was cloned into four different but compatible plasmids (Supplementary Table 1)—pACYCDuet-1, pCDFDuet-1, pETDuet-1, and pRSFDuet-1—to generate four recombinant plasmids (Supplementary Table 2)^28^. The protein expressions from different plasmids harbored within the cells were induced with isopropyl-β-D-1-thiogalactopyranoside (IPTG), and an SDS-PAGE analysis confirmed the soluble expression of all of the enzymes used.

### Production of prochiral α-keto acid

**Fig. 3:**
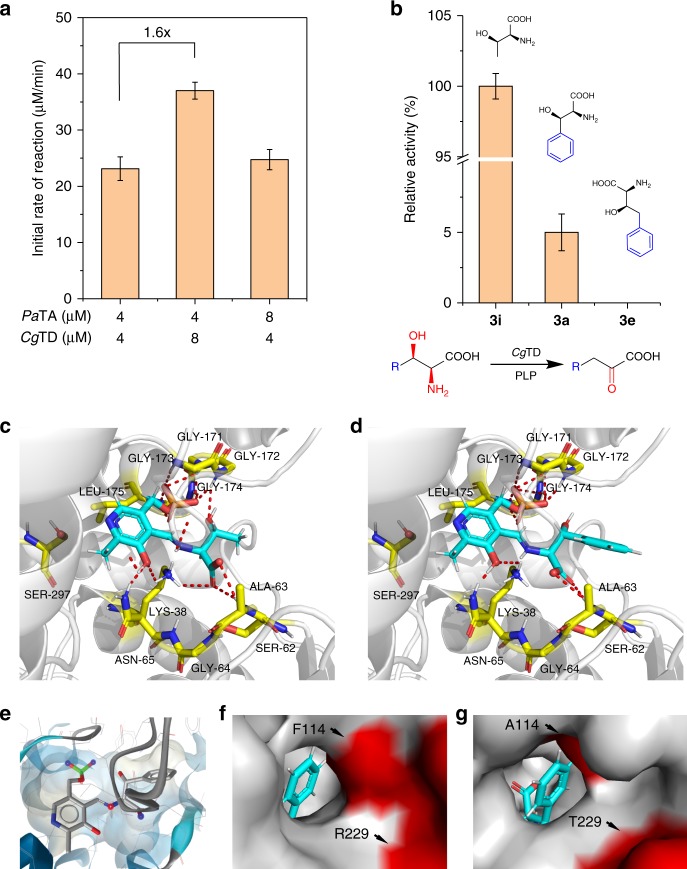
Improvement of the substrate specificity of *Cg*TD toward bulky substrates by protein engineering. **a** The effect of TA and TD concentrations on the initial reaction rate of **1a** to **4a** (reactions were performed in duplicate with *Pa*TA and *Cg*TD enzymes, with 2.5 mM aldehydes, 25 mM glycine, and 50 µM PLP). **b** *Cg*TD activity toward different substrates. **c** Docking of the PLP/**3i** complex into the active site of *Cg*TD. **d** Docking of the PLP/**3a** complex into the active site of *Cg*TD. **e** The surface of binding pocket. **f** Substrate access channel of *Cg*TD_WT_. **g** Substrate access channel of *Cg*TD^F114A,R229T^. All enzymatic assays were performed in triplicate, and error bars indicate ±s.d.

The validation of this system was initially focused on building a capable BM (Fig. 1b); therefore, the talented enzymes TA and TD were needed. First, *Pseudomonas aeruginosa* TA (*Pa*TA) was selected owing to its high activity toward **1a** and **2**^31,32^. Then, for TD screening, eight functionally known TDs were selected from the NCBI database via data mining^33^ and expressed in *E. coli* BL21 (DE3). In order to obtain a complete conversion of **3** to **4**, the TD activities toward both *threo*- and *erythro*-ʟ-phenylserine (*threo*/*erythro*-**3a**) were tested, and only TD from *Corynebacterium glutamicum* (*Cg*TD) showed activities toward both configurations. Therefore, *Pa*TA and *Cg*TD were overexpressed and purified (Supplementary Fig. 3). To identify the potential bottleneck in the BM, the effects of the TA and TD concentrations on the initial reaction rate of **1a** to **4a** were examined in vitro. As shown in Fig. 3a, the initial reaction rate of **1a** to **4a** was slightly increased (7%) when increasing the TA concentration by twofold, while it was increased by 1.6-fold when doubling the concentration of *Cg*TD. These results indicate that *Cg*TD was the rate-limiting enzyme in the C–C bond formation and α,β-elimination cascade reaction. Furthermore, the activity of *Cg*TD toward different substrates was investigated, and the results are shown in Fig. 3b. It was found that the activity of *Cg*TD significantly decreases as the substrate size increased. In comparison with the case using **3i** as a substrate (a natural substrate of *Cg*TD), the relative activity of *Cg*TD toward bulky substrate **1a** decreased to 5% and exhibited no activity toward larger substrate **3e** (Fig. 3b). Therefore, to meet the needs of α-keto acid synthesis, it is imperative to improve the substrate specificity of *Cg*TD toward bulky substrates by protein engineering strategies.

Then, we built a structural model of *Cg*TD via homologous modeling by using *E. coli* TD (PDB ID: 1TDJ) as the template. To simulate the orientation of the external aldimine intermediate^34^, ligands (**3a** and **3i**) were created with the nitrogen atom of **3** linked to the PLP cofactor (Fig. 3c, d). Docking these ligands into the cofactor-free structure yielded outputs with the PLP and substrates with approximately the same orientation, suggesting that bulky substrates show the correct orientation. The surface of the binding pocket (Fig. 3e) also indicated that the volume of the catalytic cavity is sufficiently large to accept bulky substrates. On the other hand, by analyzing the substrate channel of *Cg*TD, steric hindrance was identified as a potential bottleneck (a small entrance, Fig. 3f) for the bulky substrates. Two residues (Phe114 and Arg229) were identified as potential hot spots for broadening the substrate channel. Semisaturated mutation was used for protein engineering, and the highest initial reaction rate (667 µM min^−1^) was observed for **1a** with the variant *Cg*TD^F114A,R229T^ (with a larger substrate channel, Fig. 3g), thus representing a greater-than-18-fold increase when compared with that of *Cg*TD_WT_ for **1a** (37 µM min^−1^).

Four BM plasmids containing the *Pa*TA and *Cg*TD^F114A,R229T^ genes were transformed into the host strain that led to *E. coli* (OA01-04) (Supplementary Table 3). Among them, *E. coli* OA02 was found to be the best whole-cell catalyst in the biotransformation of **1a** into **4a** (Supplementary Fig. 4), which produced 380 mg l^−1^ **4a**. Furthermore, the reaction conditions for the conversion of **1a** to **4a** were optimized, including the pH, temperature, *E. coli* (OA02) concentration, and cosolvents (Supplementary Fig. 5). It was found that under the optimum conditions (pH 8.0, 25 °C, 10 g dcw l^−1^, and 10% DMSO), the titer of **4a** was improved to 1494 mg l^−1^ with a conversion of 91%. However, low levels of unreacted substrate **1a** (2%) and intermediate **3a** (4%) still remained in the conversion system.

**Fig. 4:**
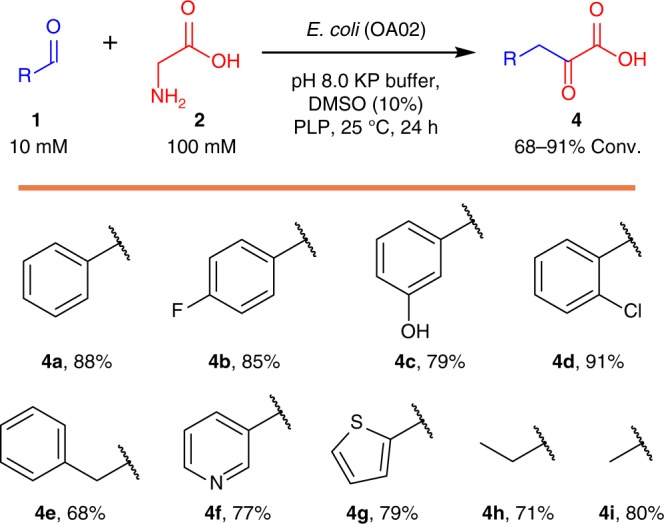
Analytical biotransformation of glycine and aldehydes **1a**–**i** to α-keto acids **4a**–**i** with *E. coli* OA02. Reactions were performed in duplicate with resting cells *E. coli* (OA02) (10 g dcw l^−1^) and **1a**–**i** (10 mM) in KP buffer (50 mM, pH 8.0, 100 µM PLP, and 10% DMSO) at 200 rpm and 25 °C for 24 h. The values are averages of two experiments

The synthetic potential of the BM was then tested with a range of aldehydes (**1b–i**), affording the corresponding α-keto acids (**4b–i**) at good conversions (68–91%) (Fig. 4). Overall, the conversion is higher than that in the case of *Pa*TA alone (conversion < 80%, d.e. < 55%)^31^, which indicated that the BM could overcome the thermodynamically unfavorable equilibria of the TA reaction by coupling it with the irreversible TD reaction. However, even with the BM cascade system, it still cannot lead to complete conversion, which is possibly due to the reversible nature of the TA reaction and the relatively low activity of *Cg*TD for the second reaction. For cascade processes, it is important that the second step is much faster than the first step to reach the theoretical conversion of 100%^28^. Another reason is the metabolic background of *E. coli*, which could convert aldehydes into carboxylic acids or the corresponding alcohols and cause a loss of the starting material^35^.

### Production of α-hydroxy acid by reintroducing chiral –OH

To efficiently reintroduce chiral –OH for converting keto acids into α-hydroxy acids, the highly selective ʟ- or D-HicDH, (ʟ-HicDH obtained from *Lactobacillus confusus* DSM 201966 and D-HicDH obtained from *Lactobacillus paracasei* DSM 20008)^36,37^ was chosen and used to construct EM1. Then, four BM plasmids and eight EM1 plasmids were combinatorially combined and transformed into *E. coli* to engineer the catalysts for the conversion of glycine and aldehydes into α-hydroxy acids. Since plasmids with the same backbone are not compatible with each other, 24 different *E. coli* strains (OA05-28) were obtained (Supplementary Table 3), each coexpressing *Pa*TA, *Cg*TD, *Cb*FDH, and highly selective ʟ- or D-LDH (*E. coli* OA05-16 and *E. coli* OA17-28 provide *S*- and *R*-selective reducing activities, respectively). Next, the capacities of those 24 types of *E. coli* strains (OA05-28) on the transformation of **1a** into (*S*)- and (*R*)-**5a** were examined, and the results showed that *E. coli* (OA15) and *E. coli* (OA23) are the best whole-cell catalysts, which produced 1470 mg l^−1^ of (*S*)-**5a** (>99% e.e. and 89% conversion; Supplementary Fig. 6) and 1404 mg l^−1^ of (*R*)-**5a** ( > 99% e.e. and 85% conversion, Supplementary Fig. 7), respectively, in 36 h. The intermediate **4a** was found at relatively low levels (<1%); this indicated that EM1 has a high conversion efficiency.

To further explore the potential of the engineered cascade, aldehydes **1b**–**i** (10 mM) were biotransformed into α-hydroxy acids by using *E. coli* (OA15) and *E. coli* (OA23). As summarized in Table 1, (*S*)- and (*R*)-**5** were obtained with 55–92% conversion with excellent enantioselectivities (96–99%), demonstrating that the enzymatic cascade comprising BM and EM1 has a high efficiency and broad applicability.

**Table 1:** Analytical biotransformation of glycine and aldehydes to α-hydroxy acids by cascade catalysis


Substrate^a^	Product	Conv (%)	Yield^b^ (mg l^−1^)	e.e.^c^ (%)
**1a**	(*S*)-**5a**	89	1470	99
	(*R*)-**5a**	85	1404	99
**1b**	(*S*)-**5b**	87	1593	99
	(*R*)-**5b**	75	1374	99
**1c**	(*S*)-**5c**	78	1413	99
	(*R*)-**5c**	81	1467	98
**1d**	(*S*)-**5d**	91	1816	99
	(*R*)-**5d**	92	1836	99
**1e**	(*S*)-**5e**	71	1272	98
	(*R*)-**5e**	55	985	96
**1f**	(*S*)-**5f**	78	1296	99
	(*R*)-**5f**	70	1163	99
**1g**	(*S*)-**5g**	71	1215	99
	(*R*)-**5g**	78	1335	99
**1h**	(*S*)-**5h**	87	1019	99
	(*R*)-**5h**	83	972	99
**1i**	(*S*)-**5i**	90	928	99
	(*R*)-**5i**	80	825	99

^a^ Reactions were performed in duplicate with resting cells of *E. coli* (OA15 or OA23) (10 g dcw l^−1^) and **1a**–**i** (10 mM) in 2 ml KP buffer (50 mM, pH 8.0, 100 µM PLP, 1 mM NAD^+^, and 10% DMSO) at 200 rpm and 25 °C for 36 h

^b^ The conversion and yields were obtained after completion of the reactions and determined by HPLC analysis. The values are averages of two experiments

^c^ Enantiomeric excess (e.e.) was determined by chiral HPLC analysis (Supplementary Figs 8-15). The values are averages of two experiments

### Production of α-amino acid by reintroducing chiral –NH_2_

In order to reintroduce the chiral –NH_2_ group, ʟ-AADH from *Bacillus badius*^38^ was selected to construct EM2 for converting α-keto acids into (*S*)-α-amino acids. The combinatorial assembly of four BM plasmids and four EM2 plasmids led to 12 different *E. coli* whole-cell catalysts (OA29-40) (Supplementary Table 3), each coexpressing *Pa*TA, *Cg*TD, *Cb*FDH, and highly selective ʟ-AADH. Consequently, the 12 constructed *E. coli* strains were used for the transformation of **1a** into (*S*)-**6a**, and *E. coli* (OA34) showed the best conversion capacities, which produced 1478 mg l^−1^ (*S*)-**6a** (90% conversion and 95% e.e., Supplementary Fig. 16). Furthermore, *E. coli* (OA34) was used to catalyze different aldehydes **1b–i** (Table 2) to the corresponding (*S*)-amino acids, and excellent conversions of 65–93% (>89% e.e.) were detected.

**Table 2:** Analytical biotransformation of glycine and aldehydes to α-amino acids by cascade biocatalysis


Substrate^a^	Product	Conv (%)	Yield^b^ (mg l^−1^)	e.e.^c^ (%)
**1a**	(*S*)-**6a**	90	1478	95
	(*R*)-**6a**	85	1396	99
**1b**	(*S*)-**6b**	80	1457	93
	(*R*)-**6b**	84	1530	99
**1c**	(*S*)-**6c**	75	1351	98
	(*R*)-**6c**	71	1279	99
**1d**	(*S*)-**6d**	93	1847	98
	(*R*)-**6d**	89	1768	99
**1e**	(*S*)-**6e**	65	1158	92
	(*R*)-**6e**	32	570	98
**1f**	(*S*)-**6f**	79	1189	96
	(*R*)-**6f**	72	1028	99
**1g**	(*S*)-**6g**	73	1243	89
	(*R*)-**6g**	64	1089	99
**1h**	(*S*)-**6h**	87	1010	97
	(*R*)-**6h**	85	987	99
**1i**	(*S*)-**6i**	91	929	98
	(*R*)-**6i**	78	796	99

^a^ Reactions were performed in duplicate with resting cells of *E. coli* (OA34 or OA59) (10 g dcw l^−1^) and **1a**–**i** (10 mM) in 2 ml KP buffer (50 mM, pH 8.0, 100 µM PLP, 1 mM NAD^+^, and 10% DMSO) at 200 rpm and 25 °C for 36 h

^b^ The conversion and yields were obtained after completion of the reactions and determined by HPLC analysis. The values are averages of two experiments

^c^ Enantiomeric excess (e.e.) was determined by chiral HPLC analysis (Supplementary Figs 19-26). The values are averages of two experiments

Similarly, in order to produce (*R*)-α-amino acids, 12 different *E. coli* strains (OA41-52) were obtained, each coexpressing *Pa*TA, *Cg*TD, glucose dehydrogenase from *Bacillus megaterium* (*Bm*GDH, for NADPH recycling), and D-AADH (a *meso*-2,6-D-diaminopimerate dehydrogenase mutant from *Symbiobacterium thermophilum*^39^, *St*DAPDH^H227V^). However, the best strain of *E. coli* (OA51) produced only 370 mg l^−1^ (*R*)-**6a** (Supplementary Fig. 17). In order to improve the conversion of (*R*)-α-amino acids, we recruited the transamination route in EM3 (expressing *B. subtilis* D-AATA) (Fig. 1b) since D-AADHs are not ubiquitous in nature as opposed to ʟ-selective AADHs (only *meso*-DAPDHs have been reported)^30^. Thus, four BM plasmids and four EM3 plasmids were combinatorially assembled to obtain 12 *E. coli* strains (OA53-64) (Supplementary Table 3). The 12 obtained types of *E. coli* strains were used for the conversion of **1a** into (*R*)-**6a**. Finally, the highest titer of (*R*)-**6a** (1396 mg l^−1^ with 85% conversion and 99% e.e., Supplementary Fig. 18) was obtained with *E. coli* (OA59). The potential of *E. coli* (OA59) was examined with aldehydes **1b–i** (Table 2), and (*R*)-**6** was produced with conversions of 71–89%, except (*R*)-**6e** (32%) and (*R*)-**6g** (64%). Remarkably, all of the produced (*R*)-α-amino acids **6a**–**i** were in their enantiopure forms (e.e. >98%). In conclusion, the enzymatic cascade coupling BM with EM2/EM3 exhibited great potential for the synthesis of (*S*)/(*R*)-α-amino acids from renewable glycine and aldehydes.

### Synthesis of α-functionalized organic acids on a 100-ml scale

The synthesis of α-functionalized organic acids was scaled up to 100 ml to further evaluate the feasibility of the biocatalytic cascades. First, the tolerance of the key enzyme *Cg*TD to **1a** and the ratio of the catalyst to substrate **1a** were optimized for the scaled-up biotransformations, and the optimum conditions were identified to be 50 mM **1a** with catalyst-to-substrate ratios of 0.5–1:1 (g dcw l^−1^ mM^−1^) at 25 °C, pH 8.0, and 5% DMSO (Supplementary Fig. 27). Then, the preparative biotransformations of **1a** (50 mM) with *E. coli* (OA02), *E. coli* (OA23), and *E. coli* (OA59) (30 g dcw l^−l^) were performed. An additional 40-mM **1a** was added after 6 h when the remaining concentration of **1a** was lower than 10 mM. Subsequently, a total of 12.5 g l^−1^ **4a**, 12.1 g l^−1^ (*R*)-**5a** (99% e.e.), and 11.9 g l^−1^ (*R*)-**6a** (99% e.e.) was produced at 85, 81, and 80% conversion, respectively, in 30–42 h. Further, the space–time yields (STYs) of **4a**, (*R*)-**5a**, and (*R*)-**6a** reached 10.0, 6.9, and 6.8 g l^−1^ d^−1^, respectively. Finally, **4a**, (*R*)-**5a**, and (*R*)-**6a** were isolated at 75, 69, and 61% yield by chromatography, extraction, and crystallization. Similarly, seven other valuable chemicals (**4i**, (*S*)-**5e**, (*S*)-**5i**, (*R*)-**5i**, (*S*)-**6e**, (*S*)-**6g**, and (*R*)-**6h**) with many applications (Supplementary Table 4)) were successfully synthesized using the corresponding *E. coli* whole-cell catalysts and were isolated in yields of 45–78%.

## Discussion

In this study, we report an artificially designed biocatalytic cascade, which comprises a BM and three different EMs, for the C–H functionalization-mediated asymmetric synthesis of α-functionalized organic acids. This biocatalytic cascade represents a chiral-group-resetting process that operates in a modular assembly manner, enabling the change of different catalyst modules and substrate units to facilitate the control of product species and configurations. Using this biocatalytic process with glycine as a primer and different aldehydes as extender units, 45 types of aliphatic, aromatic, heteroaromatic and heterocyclic products were obtained, including nine prochiral α-keto acids, 18 chiral α-hydroxy acids, and 18 chiral α-amino acids. Therefore, this biocatalytic process exhibited great potential for the production of a variety of α-functionalized organic acids.

The chiral group resetting process reported here provides a useful tool for the regulation of product configurations and species and helps to expand the application of unsatisfactory enzymes (such as TAs). During this process, the chiral information of each product was changed via three steps (Fig. 2): (i) introduce chiral –OH/–NH_2_ groups (low selectivity), (ii) delete chiral –OH/–NH_2_ groups, and (iii) reintroduce –OH/–NH_2_ groups (with the desired configurations and species). With this process, both optically pure α-amino acids and α-hydroxy acids were finally obtained. Furthermore, this artificially designed biocatalytic process also overcame the thermodynamic (reversible reaction) and kinetic limitations (low diastereoselectivity for β-carbon) associated with the use of TAs^31,40,41^ by cascading with an irreversible TD reaction. From an organic synthesis perspective, for the regulation of product configurations and species, the chiral-group-resetting process is unique and advantageous over the majority of other enzymatic processes, e.g., asymmetric reduction (from achiral to *S*/*R*-NH_2_/-OH)^3,39^, stereoinversion (from *S*-NH_2_/-OH to *R*-NH_2_/-OH)^3–5^, deracemization (from *rac*-NH_2_/-OH to *R*-NH_2_/-OH)^3–5^, and type conversion (from *S*-NH_2_ to *S*/*R*-OH)^26,36,37^. In related chemical methods, the product configurations and species are the same as the substrate since the active chiral functional groups (e.g., –OH and –NH_2_)^10,11^ were protected.

The achiral glycine and aldehydes, which are readily available and inexpensive, for the production of α-hydroxy acids and α-amino acids represent a sustainable alternative to other complementary methods. In contrast, in most enzymatic production processes of α-hydroxy acids and α-amino acids, the commonly used substrates are keto acids^3,39^, (*S*)-amino acids or their racemic mixtures^3,26,36^, and (*S*)-hydroxy acids or their racemic mixtures^4,5^; these are not readily available and much more expensive than the corresponding aldehydes. Regarding chemical methods, the protection and deprotection processes have the particular drawback of cost due to the protected substrates that are needed, such as *N*-phthalimido-protected L-α-alanine^11^ and methyl or benzyl-protected (*S*)-α-lactic acid^10^. Thus, this biocatalytic process provides an attractive strategy for the conversion of low-cost achiral starting materials to high-value α-functionalized organic acids.

The biocatalytic process in this study is elegant and nicely exploits the power of cascades: (i) it provides a universal, efficient, and renewable catalyst to replace precious transition metals such as Pd^10,11^ in the modern chemical C–H functionalization-mediated asymmetric assembly of α-functionalized organic acids and (ii) when compared to other enzymatic methods^3–5,9,26,28,36,39^, the universality and practicality of this biocatalytic cascade is more attractive owing to the more diverse product range that it supports, including liphatic, aromatic, heteroaromatic, and heterocyclic products from three classes (α-keto/hydroxy/amino acids). Furthermore, since enantiocomplementary ʟ- and D-specific TAs were identified to accept nucleophilic substrates other than glycine (such as alanine, serine, and cysteine)^42,43^, the expansion of this biocatalytic process could enable the synthesis of a vast array of α-functionalized products simply by combinatorically integrating different components such as other catalyst modules and substrate units.

## Methods

### Strains and plasmids

Wild-type *E. coli* BL21 DE3 was used for all molecular cloning and biocatalysis experiments. Plasmid based gene overexpression was achieved by cloning the desired gene(s) into a set of compatible plasmids pACYCDuet-1, pCDFDuet-1, pETDuet-1, and pRSFDuet-1 (Novagen, Darmstadt, Germany) (Supplementary Table 1) digested with appropriate restriction enzymes (see Supplementary Methods for genetic constructions). Further transformation of constructed plamid(s) into *E. coli* strain gave an *E. coli* strain containing one or two designed modules for the desired transformations. All resulting plasmids and strains used in this study are listed in Supplementary Tables 2 and 3.

### Docking simulations

The structure model of *Cg*TD and its variants were built via SWISS-MODEL [https://swissmodel.expasy.org/] by using *E. coli* TD (PDB ID: 1TDJ [https://www.rcsb.org/structure/1TDJ]) as template. Docking simulations were performed using Autodock Vina and *Cg*TD models. BioLuminate (Schrödinger) was used to draw ligands, and AutoDockTools was used to generate PDBQT files. To simulate the orientation of the external aldimine intermediate, ligands were created having the nitrogen atom linked to the PLP cofactor.

### Enzyme mutagenesis

Site-directed mutagenesis was performed by PCR using mutagenic primers and plasmid pETduet-*Cg*TD as template according to the manufacturer’s instructions of QuickChange (Stratagene) (see Supplementary Methods for directed evolution experiments). The primers used in this study are listed in Supplementary Table 5. The *Dpn*I-digested PCR product of 3 μl was used to transform 80 μl of *E. coli* JM109 chemically competent cells and colonies after transformation were incubated for DNA sequencing until all the designed mutants were obtained. Subsequently the plasmid of each mutant was extracted and transformed into *E. coli* BL21(DE3) for enzyme expression.

### Biotransformation procedures

Analytic transformation of **1a**–**i** (10 mM) to **4a**–**i** were conducted with resting cells of *E. coli* (OA02) (10 g dcw l^−1^) in 2 ml KP buffer (50 mM, pH 8.0, 100 µM PLP, and 10% DMSO) at 200 rpm and 25 °C for 24 h. Analytic transformation of **1a**–**i** (10 mM) to (*S*)- and (*R*)-5a–i were conducted with resting cells of *E. coli* (OA15) and *E. coli* (OA23) (10 g dcw l^−1^) in 2 ml KP buffer (50 mM, pH 8.0, 100 µM PLP, and 10% DMSO) at 200 rpm and 25 °C for 36 h, respectively. Analytic transformation of **1a**–**i** (10 mM) to (*S*)- and (*R*)-**6a**–**i** were conducted with and resting cells of *E. coli* (OA33) and *E. coli* (OA71) (10 g dcw l^-1^) in 2 ml KP buffer (50 mM, pH 8.0, 100 µM PLP, and 10% DMSO) at 200 rpm and 25 °C for 36 h, respectively. At the end of the reaction, 100 μl of supernatant was separated after centrifugation (13000 g, 2 min), diluted with MeCN/H_2_O solution (900 μl, 5:4) containing 0.1% TFA. The protein was removed by centrifugation and the solution was filtered through 0.22 µm membrane filters. The resulting solution was analyzed by HPLC for quantifying the products under the conditions stated below. To determine the e.e. of 5**a**–**i**, the sample (200 μl) at the end of reaction was separated after centrifugation (13,000 g, 2 min), acidified with HCl (2.0 M) and extracted with EtOAc. After evaporation of ethyl acetate, the residue was dissolved in solvent (*n*-hexane: IPA = 95:5) for chiral HPLC analysis (see Supplementary Methods for analysis methods).

Preparative transformation of **1a** or **1i** (50 mM initial concentration and 40 mM addition at 6 h; total 90 mM) to **4a** or **4i** was conducted with resting cells of *E. coli* (OA02) (30 g dcw l^-1^) in 100 ml KP buffer (50 mM, pH 8.0, 100 µM PLP, and 5% DMSO) at 200 rpm and 25 °C for 30 h. Preparative transformation of **1a**, **1e**, or **1i** (50 mM initial concentration and 40 mM addition at 6 h; total 90 mM) to (*S*)-**5e**, (*S*)-**5i**, or (*R*)-**5a** was conducted with resting cells of *E. coli* (OA15) or *E. coli* (OA23) (30 g dcw l^-1^) in 100 ml KP buffer (50 mM, pH 8.0, 100 µM PLP, and 5% DMSO) at 200 rpm and 25 °C for 42 h, respectively. Preparative transformation of **1a**, **1e**, **1** **g**, or **1** **h** (50 mM initial concentration and 40 mM addition at 6 h; total 90 mM) to (*S*)-**6e**, (*S*)-**6g**, (*R*)-**6a**, or (*R*)-**6h** was conducted with and resting cells of *E. coli* (OA33) or *E. coli* (OA71) (30 g dcw l^-1^) in 100 ml KP buffer (50 mM, pH 8.0, 100 µM PLP, and 5% DMSO) at 200 rpm and 25 °C for 42 h, respectively. At the end of the reaction, the reaction mixtures were centrifuged at 6000 × g for 30 min, and the supernatants was collected for product isolation (see Supplementary Methods for isolation protocols). The isolated products were further identified by nuclear magnetic resonance (NMR) and high resolution mass spectrometry (HRMS) analysis (see Supplementary Figs 28–47 and Supplementary Notes).

## Electronic supplementary material

Supplementary Information

## Data Availability

The data supporting the findings of this study are available within the paper and its Supplementary Information files, or are available from the authors upon request.
