# Supplementary material for: Asymmetric assembly of high-value α-functionalized organic acids using a biocatalytic chiral-group-resetting process
Source: Nat Commun. 2018 Sep 19;9:3818. doi: 10.1038/s41467-018-06241-x (PMC6145935; doi:10.1038/s41467-018-06241-x)
Supplement: Supplementary file 1 — Supplementary Information [file 41467_2018_6241_MOESM1_ESM.pdf]

Supplementary Information

Asymmetric assembly of high-value α-functionalized organic acids using a biocatalytic chiral-group-resetting process

Wei Song[a,b], Jin-Hui Wang[a,b], Jing Wu[c], Jia Liu[a,b], Xiu-Lai Chen[a,b], Li-Ming Liu[a,b,d]*

[a]State Key Laboratory of Food Science and Technology, Jiangnan University, Wuxi 214122, China;

[b]Key Laboratory of Industrial Biotechnology, Ministry of Education, Jiangnan University, Wuxi 214122, China;

[c]School of Pharmaceutical Sciences, Jiangnan University, Wuxi 214122, China;

[d]National Engineering Laboratory for Cereal Fermentation Technology, Jiangnan University, Wuxi 214122, China.

*Corresponding author: Correspondence to Li-Ming Liu.

E-mail address: mingll@jiangnan.edu.cn (Li-Ming Liu)

Table of contents:

1. **Supplementary Figures 1-47**
2. **Supplementary Tables 1-5**
3. **Supplementary Methods**
4. **Supplementary Notes**
5. **Supplementary References**

Supplementary Figures

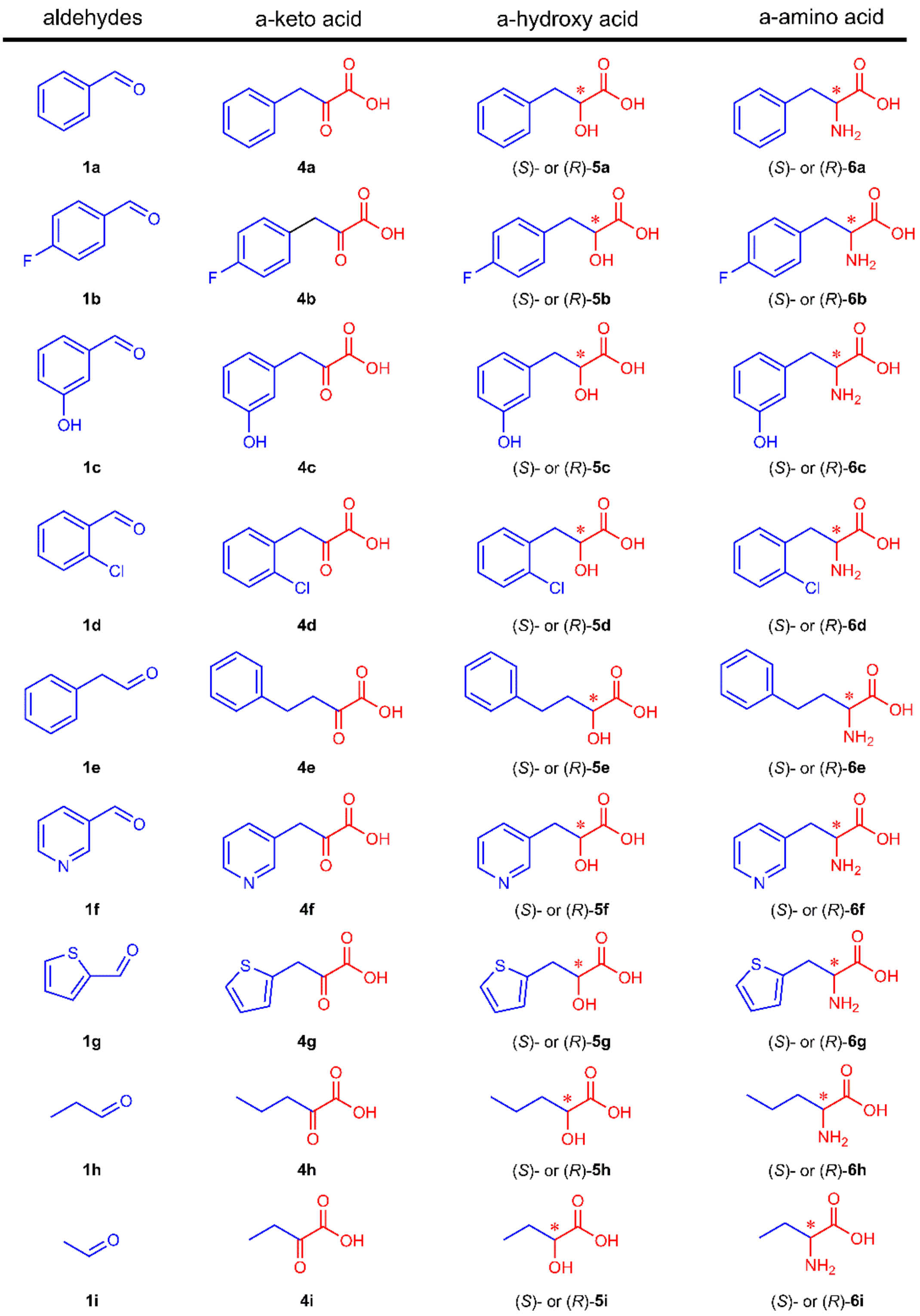

Supplementary Figure 1. Substrates and corresponding products. **1a-i**: selected aldehyde substrates. **4a-i**: α-keto acid products. (*S*)- and (*R*)-**5a-i**: chiral α-hydroxy acid products. (*S*)- and (*R*)-**6a-i**: chiral α-amino acid products.

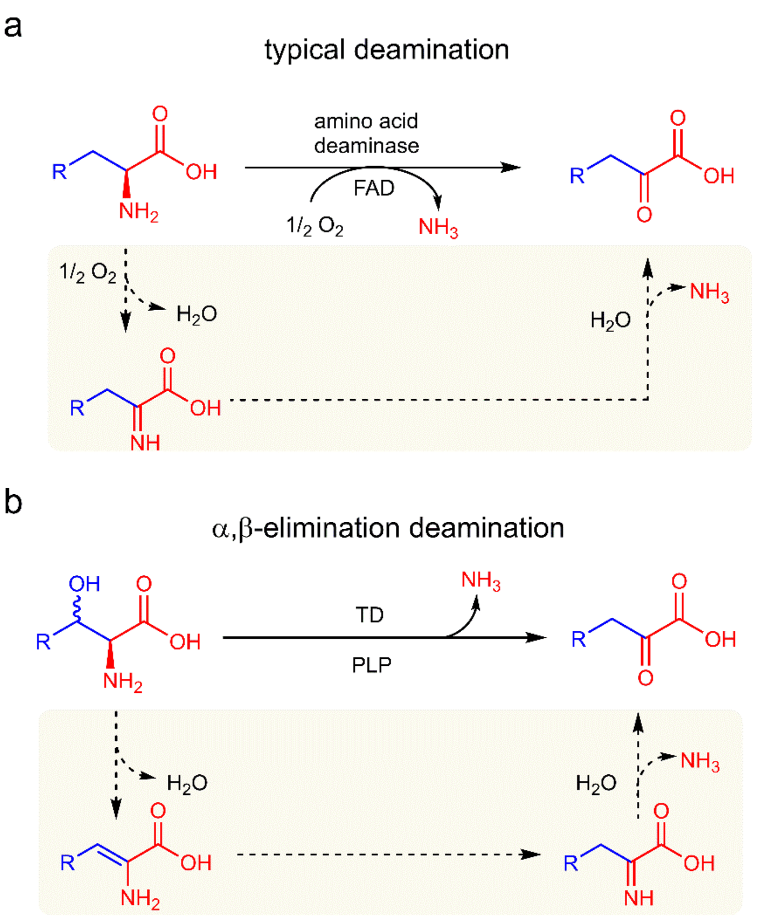

Supplementary Figure 2. Typical (**a**) deamination and α,β-elimination deamination (**b**) reactions. (**a**) Reaction mechanism of typical deamination. Typical deamination catalysed by amino acid deaminase employ FAD catalyze the deamination of L-amino acids, yielding an imine, which is rapidly hydrolyzed to the corresponding α-keto acid and ammonia in the aqueous environment. (**b**) Reaction mechanism of α,β-elimination deamination. Different from normal deaminase, TD eliminate water from the β-position of β-hydroxy-α-amino acid based on PLP, yielding an enamine that tautomerizes to the imine and is rapidly hydrolyzed nonenzymatically in the aqueous environment, yielding the corresponding α-keto acid and ammonia[1].

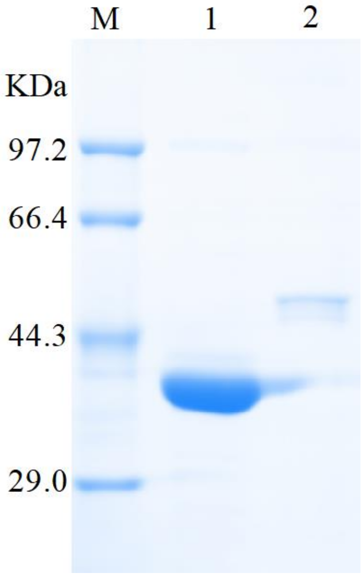

Supplementary Figure 3. Purified *Pa*TA and *Cg*TD. M: Maker; Line 1: purified *Pa*TA; Line 2: purified *Cg*TD.

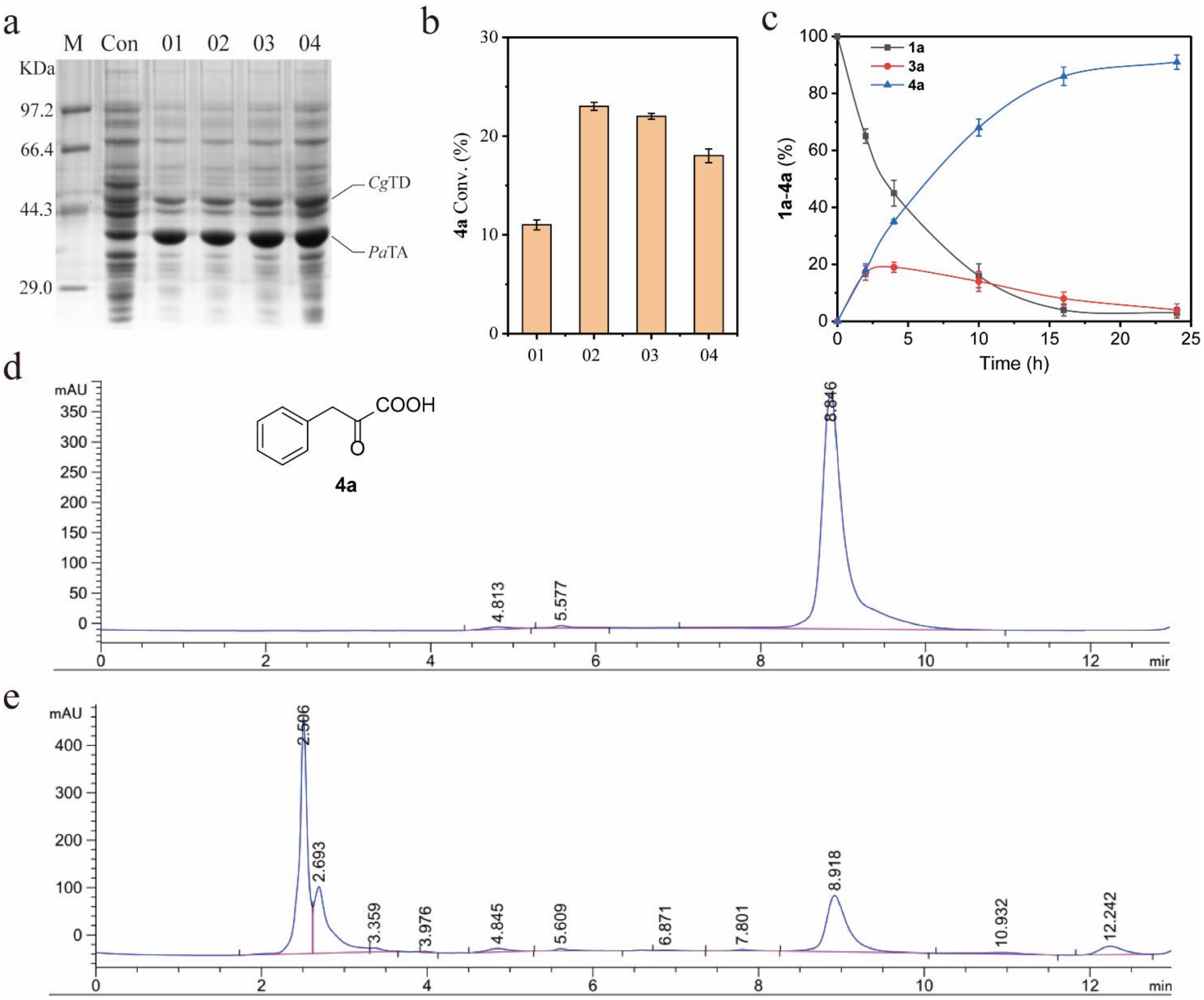

Supplementary Figure 4. Asymmetric assembly of **4a** from **1a** and **2** with engineered *E. coli* whole cell catalysts. (**a**) SDS-PAGE analysis of engineered *E. coli* (OA01-OA04) (M: marker; Con: *E. coli* cell without overexpressing any enzymes). (**b**) Conversion of **1a** to **4a** with *E. coli* (OA01-OA04). (**c**) Time course of biotransformation of **1a** to **4a** with *E. coli* OA02. (**d**) HPLC chromatogram of **4a** standard. (**e**) HPLC chromatogram of sample from biotransformation of **1a** to **4a** with *E. coli* OA02. Reactions were performed with resting cells *E. coli* (OA01-OA04) (10 g dcw l^{-1}) and **1a** (10 mM) in KP buffer (50 mM, pH 8.0, 100 µM PLP, and 10% DMSO) at 200 rpm and 25 ºC for 24 h. All biotransformations were performed in triplicate, and error bars indicate ± s.d.

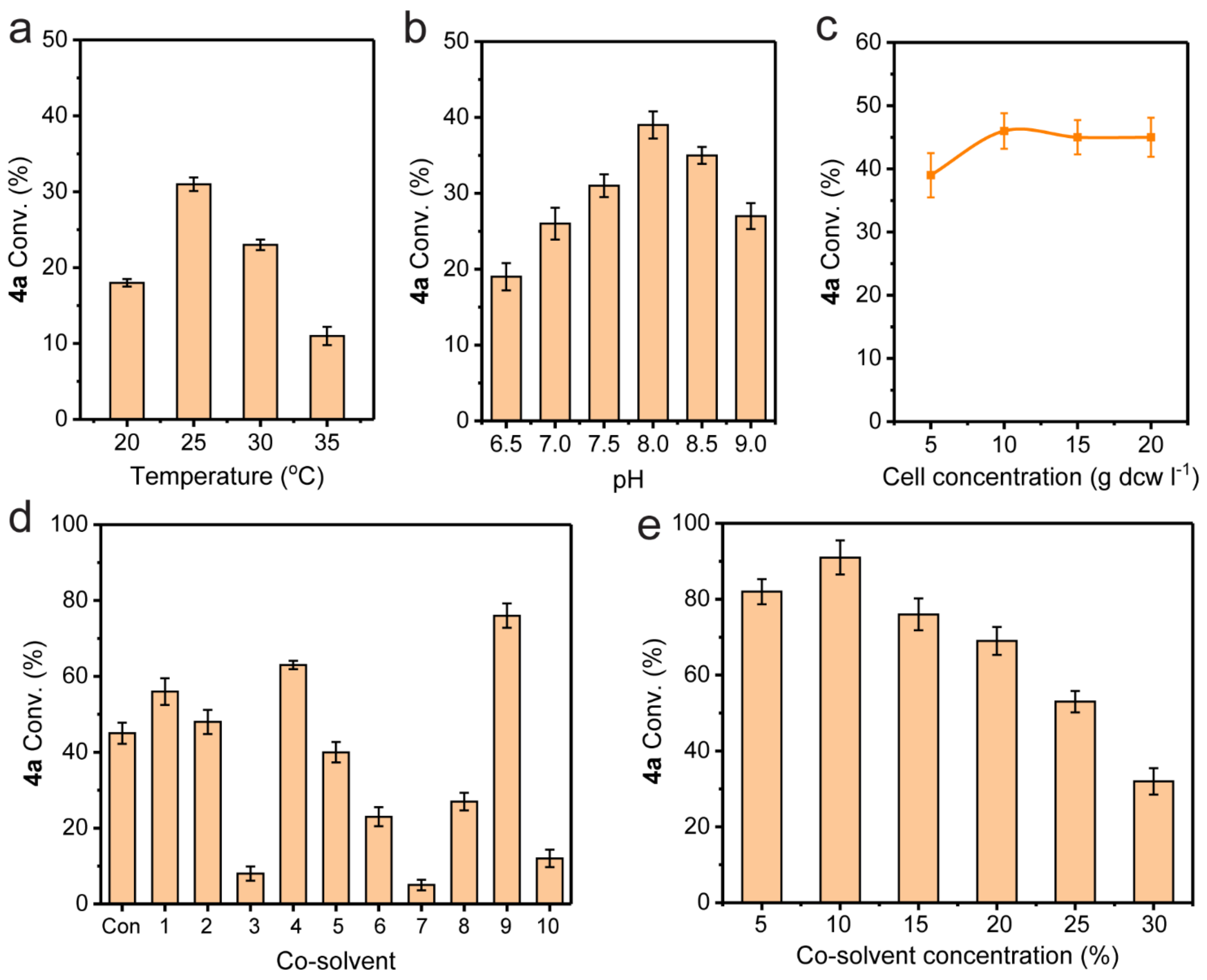

Supplementary Figure 5. Optimization of biotransformation conditions of **1a** to **4a** and **2** with *E. coli* OA02. (**a**) Temperature. (**b**) pH. (**c**) Cell concentration. (**d**) Co-solvent species, Con: *n*-hexadecane; 1: MTBE; 2: oleic acid; 3: *n*-propyl alcohol; 4: cyclohexane; 5: isooctane; 6: isopropyl ether; 7: amyl fat; 8: cyclopentane ether; 9: DMSO; 10: methyl tetrahydrofuran. (**e**) Co-solvent concentration. Initial conditions: *E. coli* (OA02) (15 g dcw l^{-1}) in KP buffer (50 mM, pH 7.5, 100 μM PLP, and 30% *n*-hexadecane at 200 rpm and 30 °C for 24 h. Optimized conditions: *E. coli* (OA02) (10 g dcw l^{-1}) in KP buffer (50 mM, pH 8.0, 100 μM PLP, and 10% DMSO) at 200 rpm and 25 °C for 24 h. All biotransformations were performed in triplicate, and error bars indicate ± s.d.

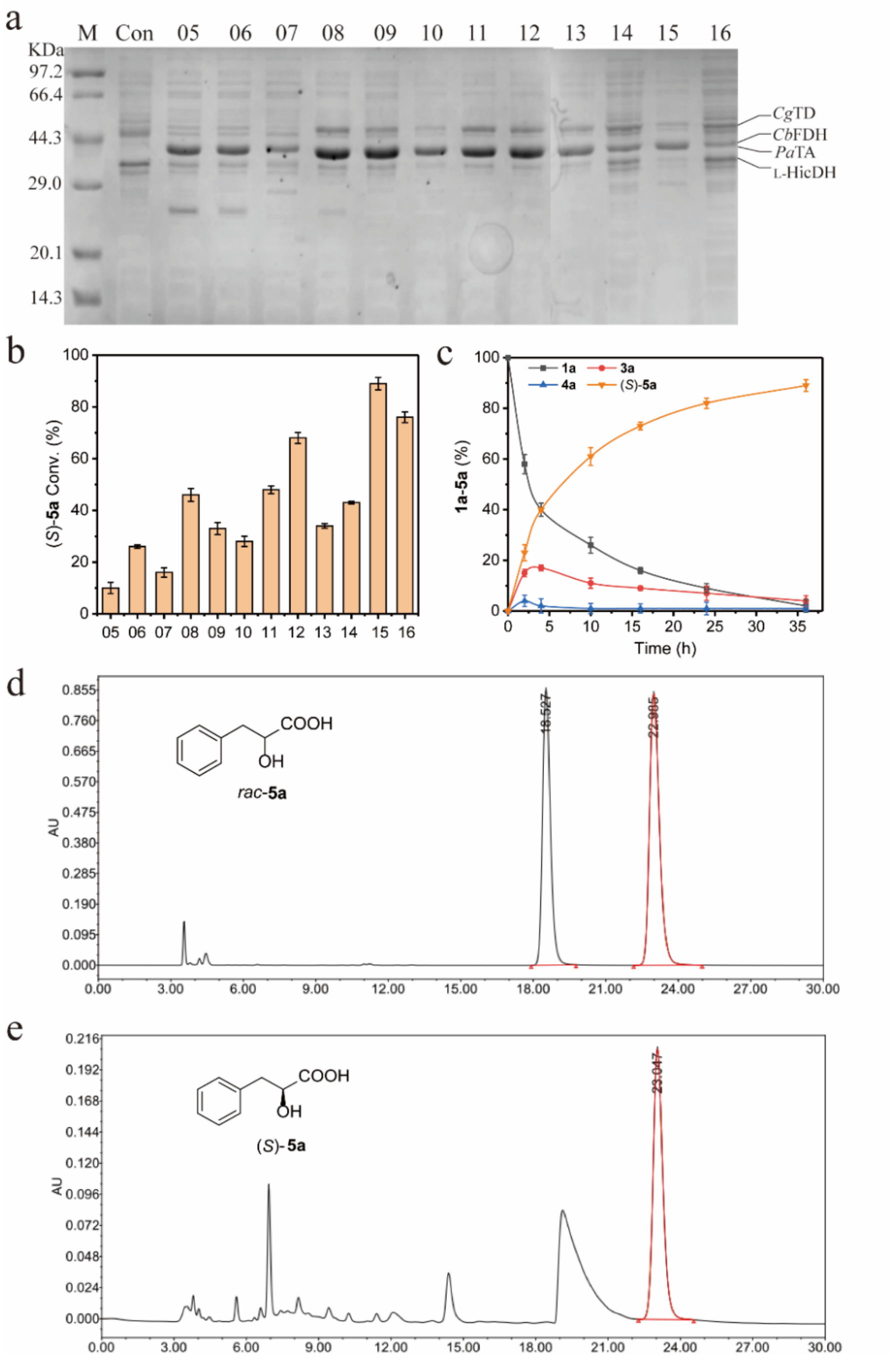

Supplementary Figure 6. Asymmetric assembly of (*S*)-**5a** from **1a** and **2** with engineered *E. coli* whole cell catalysts. (**a**) SDS-PAGE analysis of engineered *E. coli* (OA05-OA16) (M: marker; Con: *E. coli* cell without overexpressing any enzymes). (**b**) Conversion of **1a** to (*S*)-**5a** with *E. coli* (OA05-OA16). (**c**) Time course of biotransformation of **1a** to (*S*)-**5a** with *E. coli* OA15. (**d**) HPLC chromatogram of *rac*-**5a** standard. (**e**) Chiral HPLC chromatogram of sample from biotransformation of **1a** to (*S*)-**5a** with *E. coli* OA15. Reactions were performed with resting cells of *E. coli* (OA05-OA16) (10 g dcw l^{-1}) and **1a** (10 mM) in 2 ml KP buffer (50 mM, pH 8.0, 100 μM PLP, 1 mM NAD^+, and 10% DMSO) at 200 rpm and 25 °C for 36 h. All biotransformations were performed in triplicate, and error bars indicate ± s.d.

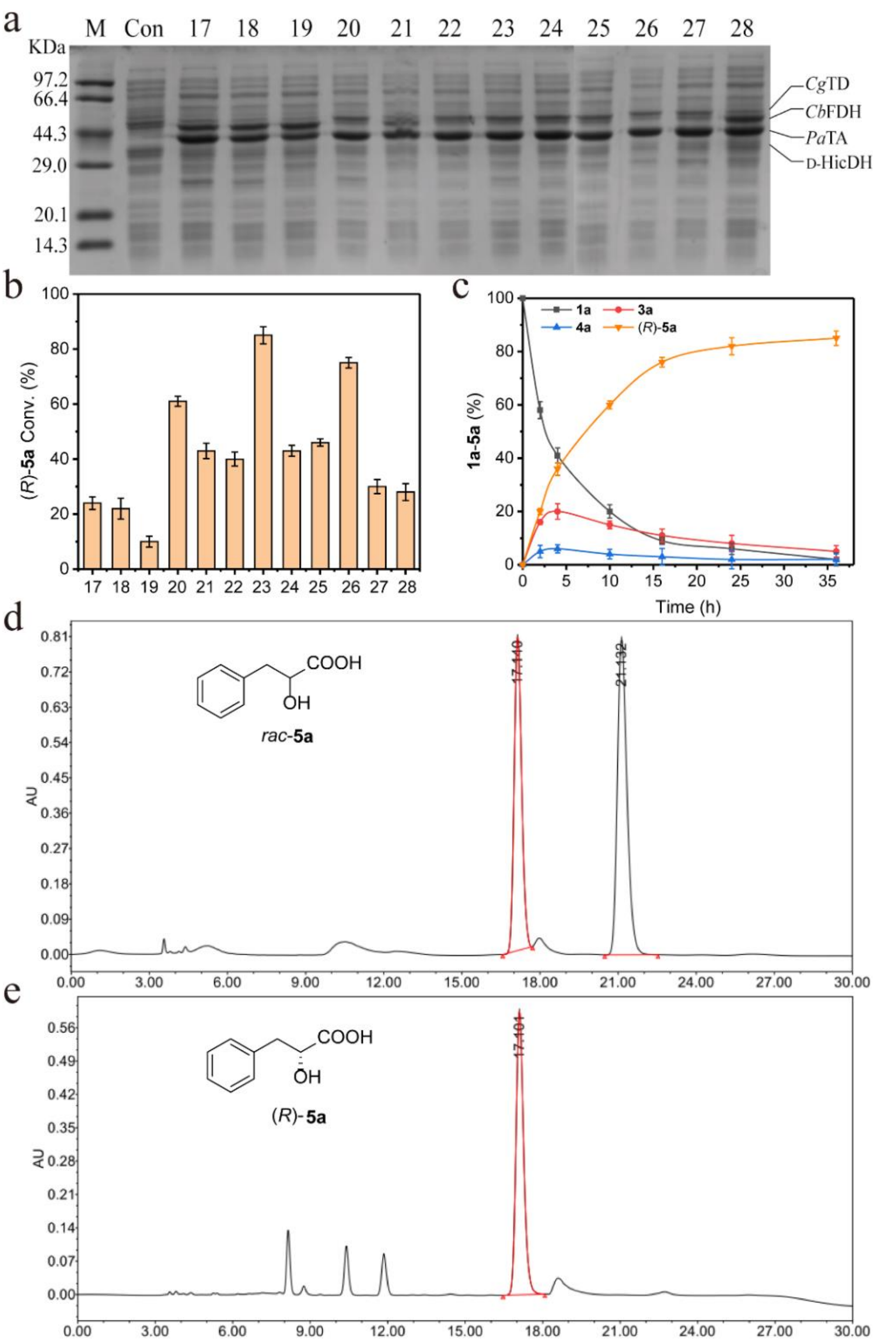

Supplementary Figure 7. Asymmetric assembly of (*R*)-**5a** from **1a** and **2** with engineered *E. coli* whole cell catalysts. (**a**) SDS-PAGE analysis of engineered *E. coli* (OA17-OA28). (**b**) Conversion of **1a** to (*R*)-**5a** with *E. coli* (OA17-OA28) (M: marker; Con: *E. coli* cell without overexpressing any enzymes). (**c**) Time course of biotransformation of **1a** to (*R*)-**5a** with *E. coli* OA23. (**d**) HPLC chromatogram of *rac*-**5a** standard. (**e**) Chiral HPLC chromatogram of sample from biotransformation of **1a** to (*R*)-**5a** with *E. coli* OA23. Reactions were performed with resting cells of *E. coli* (OA17-OA28) (10 g dcw l^{-1}) and **1a** (10 mM) in 2 ml KP buffer (50 mM, pH 8.0, 100 µM PLP, 1 mM NAD^+, and 10% DMSO) at 200 rpm and 25 ºC for 36 h. All biotransformations were performed in triplicate, and error bars indicate ± s.d.

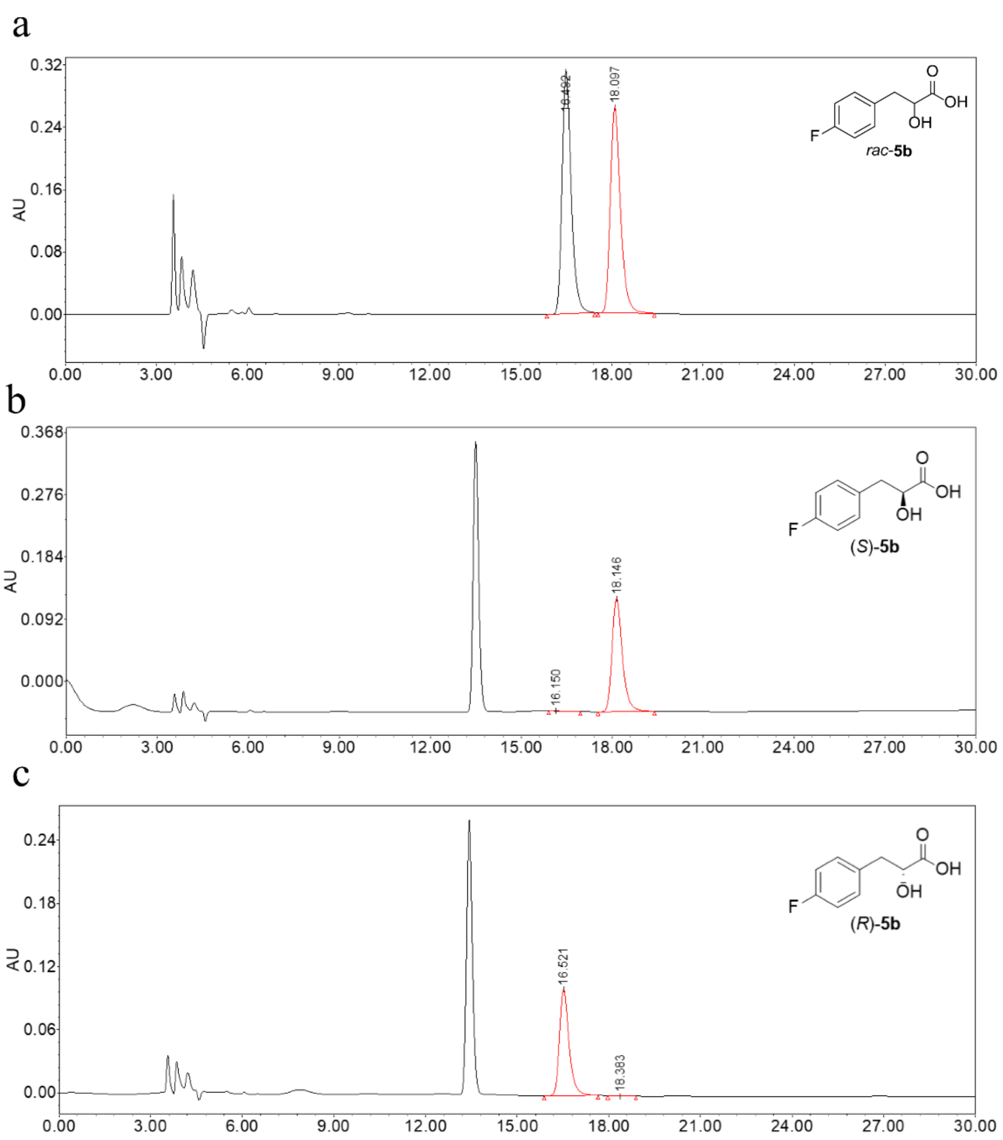

Supplementary Figure 8. Chiral HPLC chromatograms of **5b**. (**a**) Racemic **5b** standard. (**b**) Sample from biotransformation of **1b** to (*S*)-**5b**. (**c**) Sample from biotransformation of **1b** to (*R*)-**5b**.

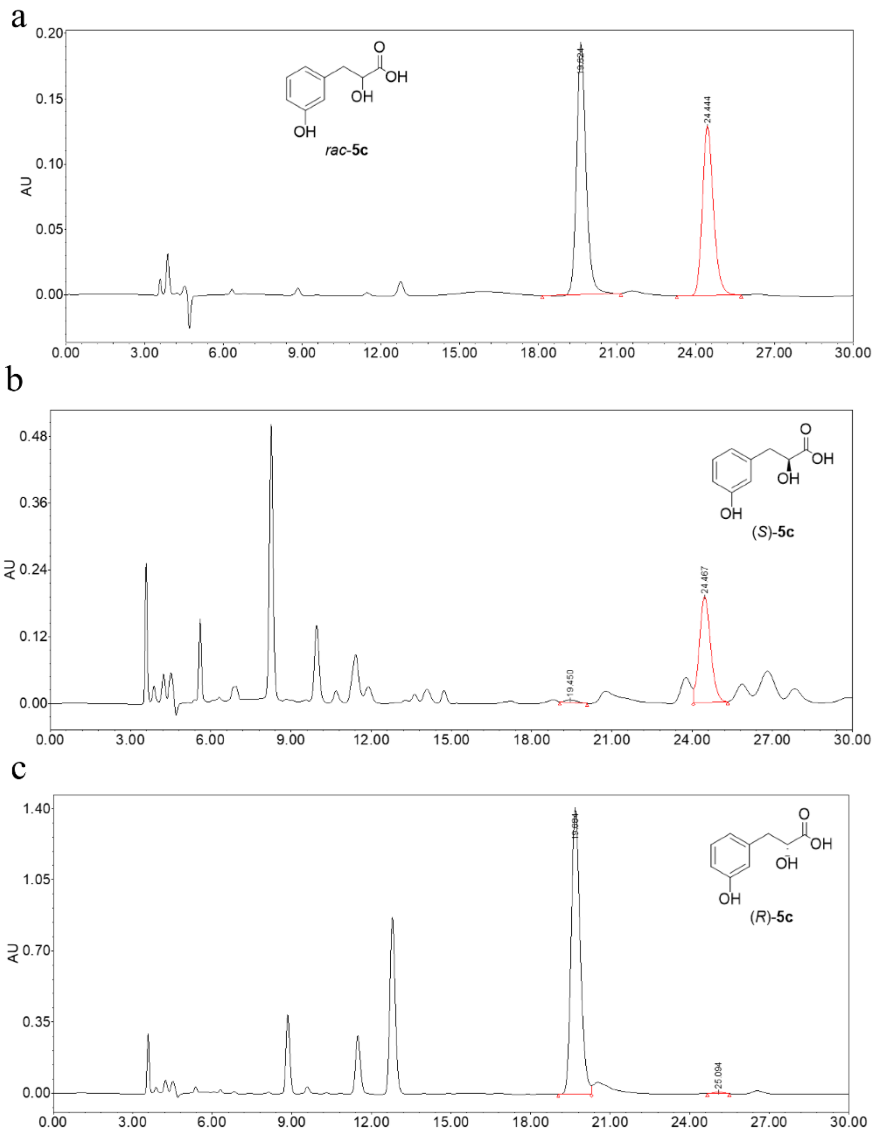

Supplementary Figure 9. Chiral HPLC chromatograms of **5c**. (**a**) Racemic **5c** standard. (**b**) Sample from biotransformation of **1c** to (*S*)-**5c**. (**c**) Sample from biotransformation of **1c** to (*R*)-**5c**.

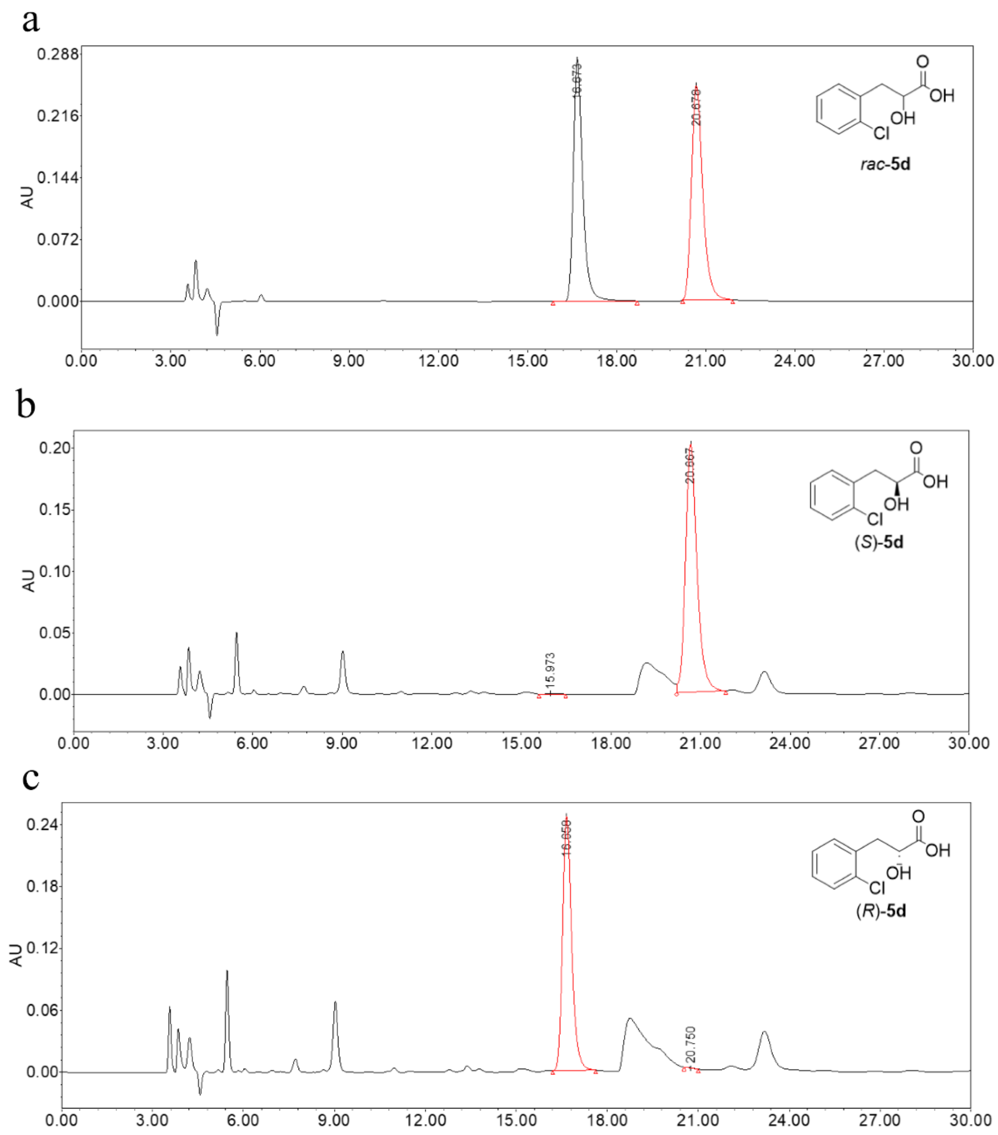

Supplementary Figure 10. Chiral HPLC chromatograms of **5d**. (**a**) Racemic **5d** standard. (**b**) Sample from biotransformation of **1d** to (*S*)-**5d**. (**c**) Sample from biotransformation of **1d** to (*R*)-**5d**.

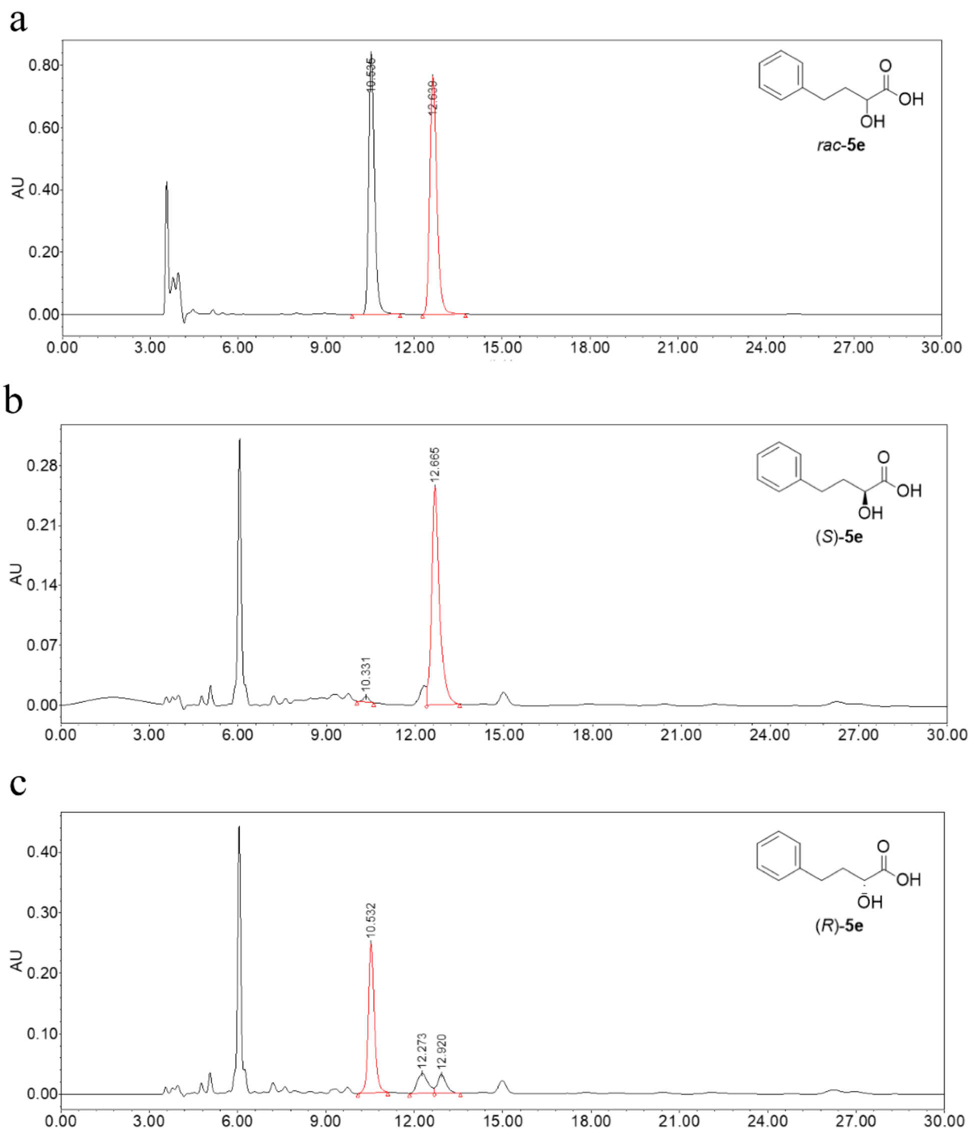

Supplementary Figure 11. Chiral HPLC chromatograms of **5e**. (**a**) Racemic **5e** standard. (**b**) Sample from biotransformation of **1e** to (*S*)-**5e**. (**c**) Sample from biotransformation of **1e** to (*R*)-**5e**.

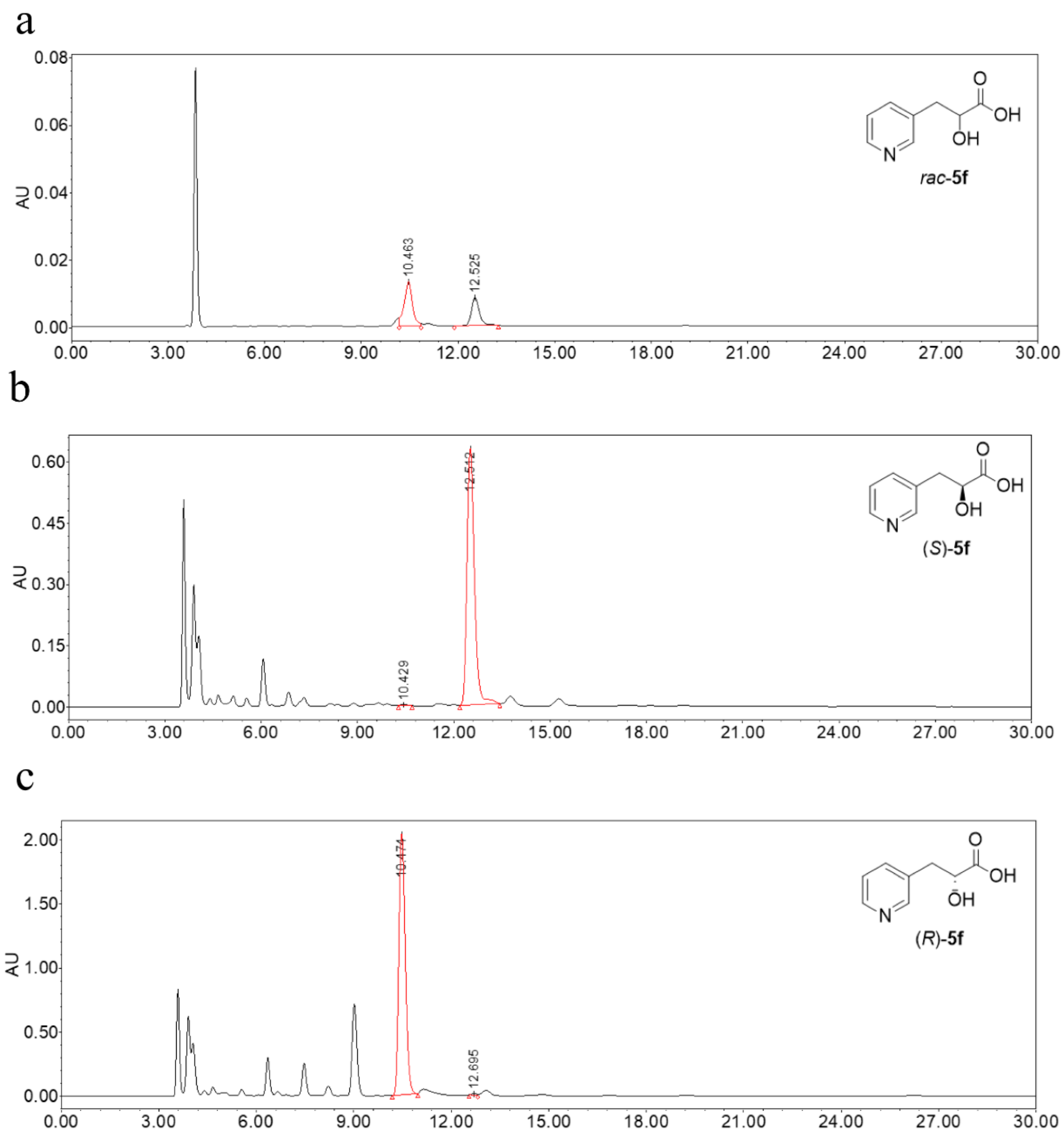

Supplementary Figure 12. Chiral HPLC chromatograms of **5f**. (**a**) Racemic **5f** standard. (**b**) Sample from biotransformation of **1f** to (*S*)-**5f**. (**c**) Sample from biotransformation of **1f** to (*R*)-**5f**.

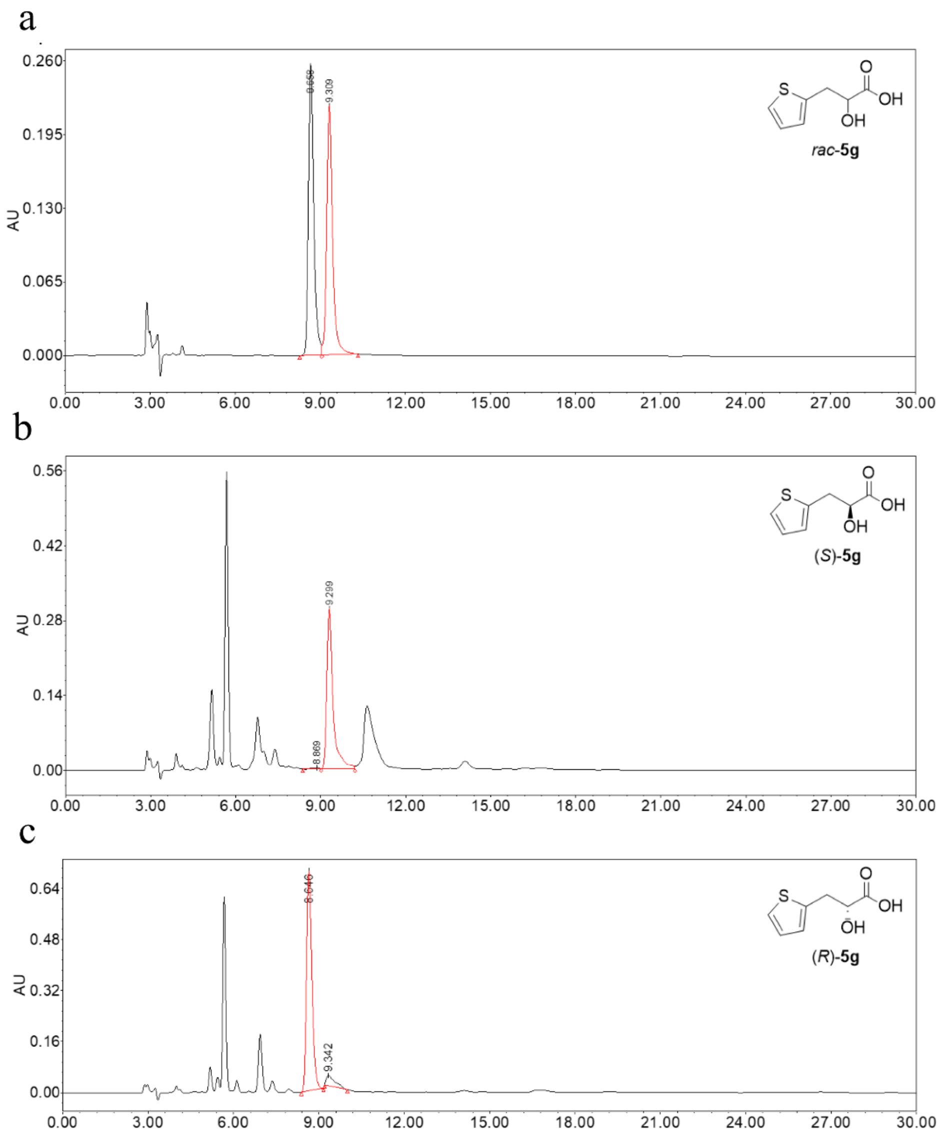

Supplementary Figure 13. Chiral HPLC chromatograms of **5g**. (**a**) Racemic **5g** standard. (**b**) Sample from biotransformation of **1g** to (*S*)-**5g**. (**c**) Sample from biotransformation of **1g** to (*R*)-**5g**.

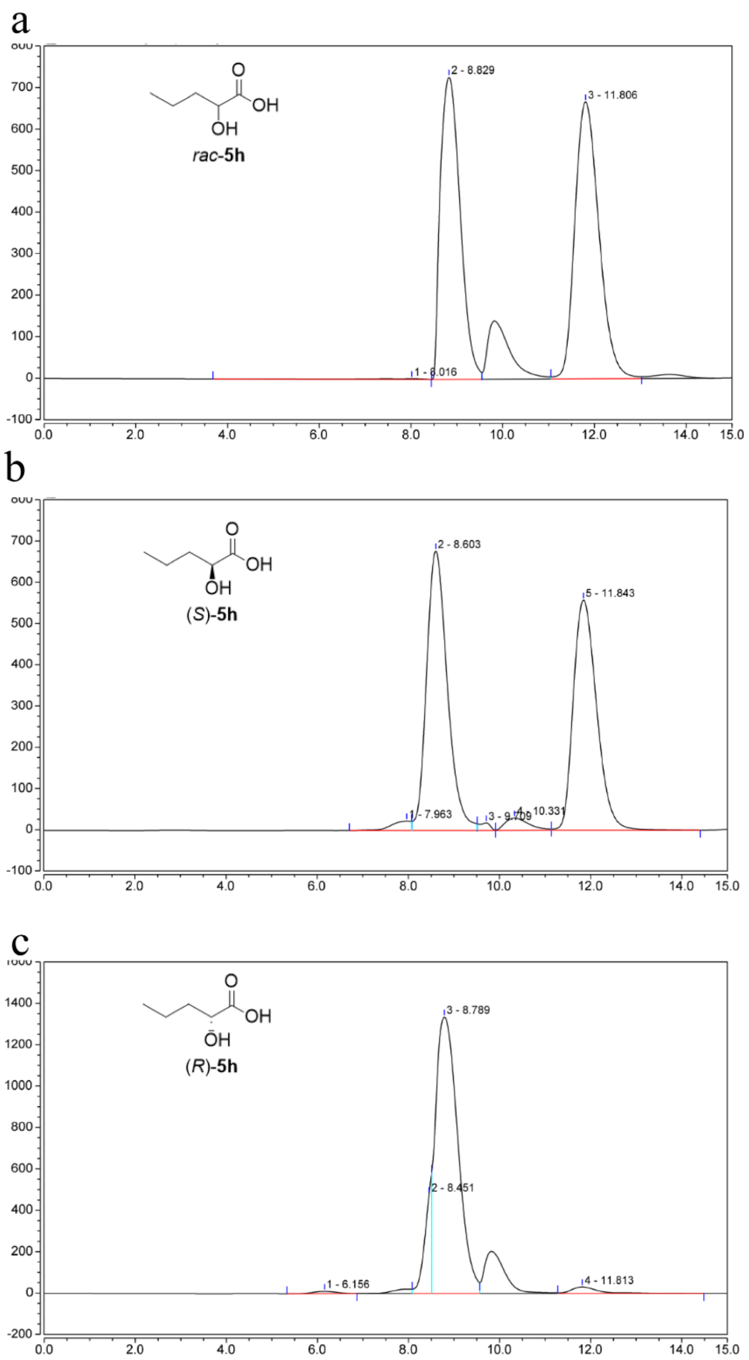

Supplementary Figure 14. Chiral HPLC chromatograms of **5h**. (**a**) Racemic **5h** standard. (**b**) Sample from biotransformation of **1h** to (*S*)-**5h**. (**c**) Sample from biotransformation of **1h** to (*R*)-**5h**.

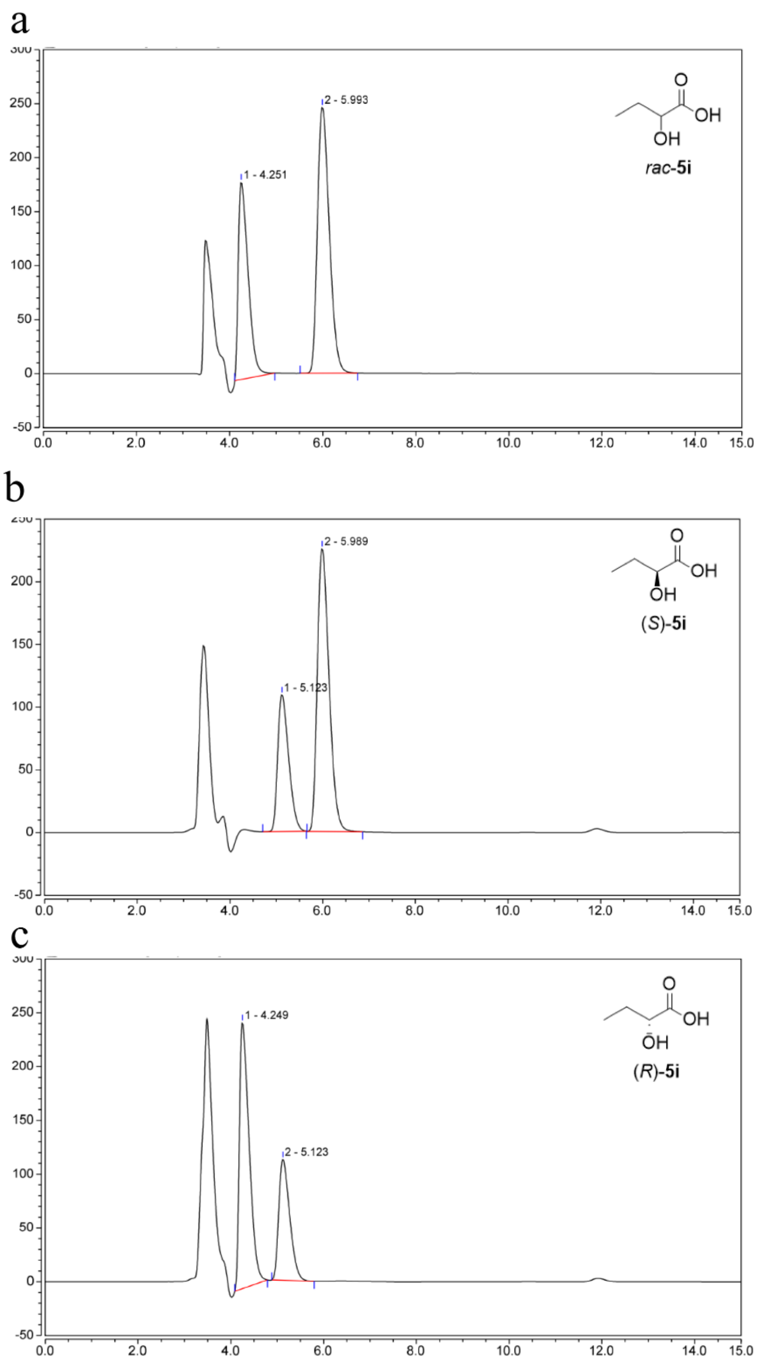

Supplementary Figure 15. Chiral HPLC chromatograms of **5i**. (**a**) Racemic **5i** standard. (**b**) Sample from biotransformation of **1i** to (*S*)-**5i**. (**c**) Sample from biotransformation of **1i** to (*R*)-**5i**.

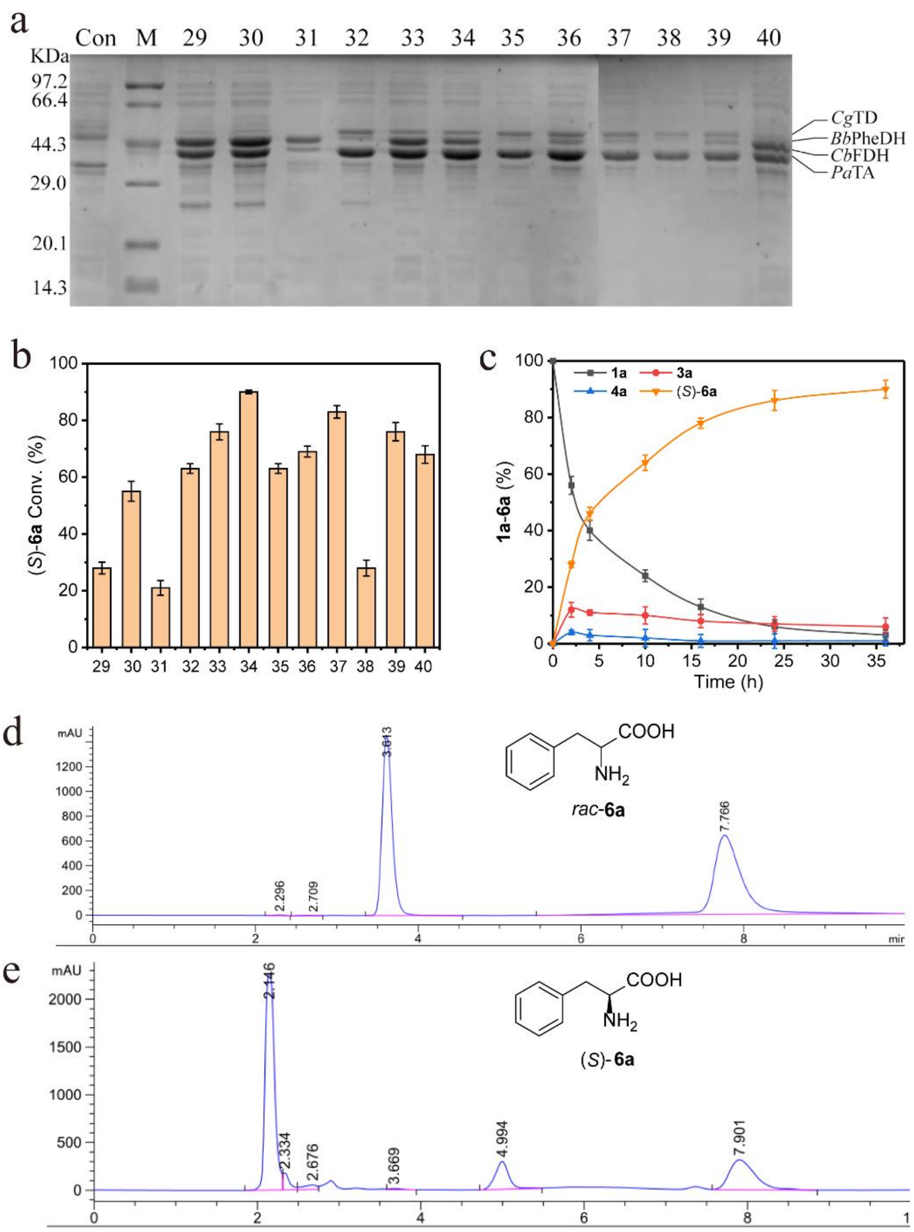

Supplementary Figure 16. Asymmetric assembly of (*S*)-**6a** from **1a** and **2** with engineered *E. coli* whole cell catalysts. (**a**) SDS-PAGE analysis of engineered *E. coli* (OA29-OA40). (**b**) Conversion of **1a** to (*S*)-**6a** with *E. coli* (OA29-OA40) (M: marker; Con: *E. coli* cell without overexpressing any enzymes). (**c**) Time course of biotransformation of **1a** to (*S*)-**6a** with *E. coli* OA34. (**d**) HPLC chromatogram of *rac*-**6a** standard. (**e**) Chiral HPLC chromatogram of sample from biotransformation of **1a** to (*S*)-**6a** with *E. coli* OA34. Reactions were performed with resting cells of *E. coli* (OA29-OA40) (10 g dcw l^{-1}) and **1a** (10 mM) in 2 ml KP buffer (50 mM, pH 8.0, 100 μM PLP, 1mM NAD^{+}, and 10% DMSO) at 200 rpm and 25 ºC for 36 h. All biotransformations were performed in triplicate, and error bars indicate ± s.d.

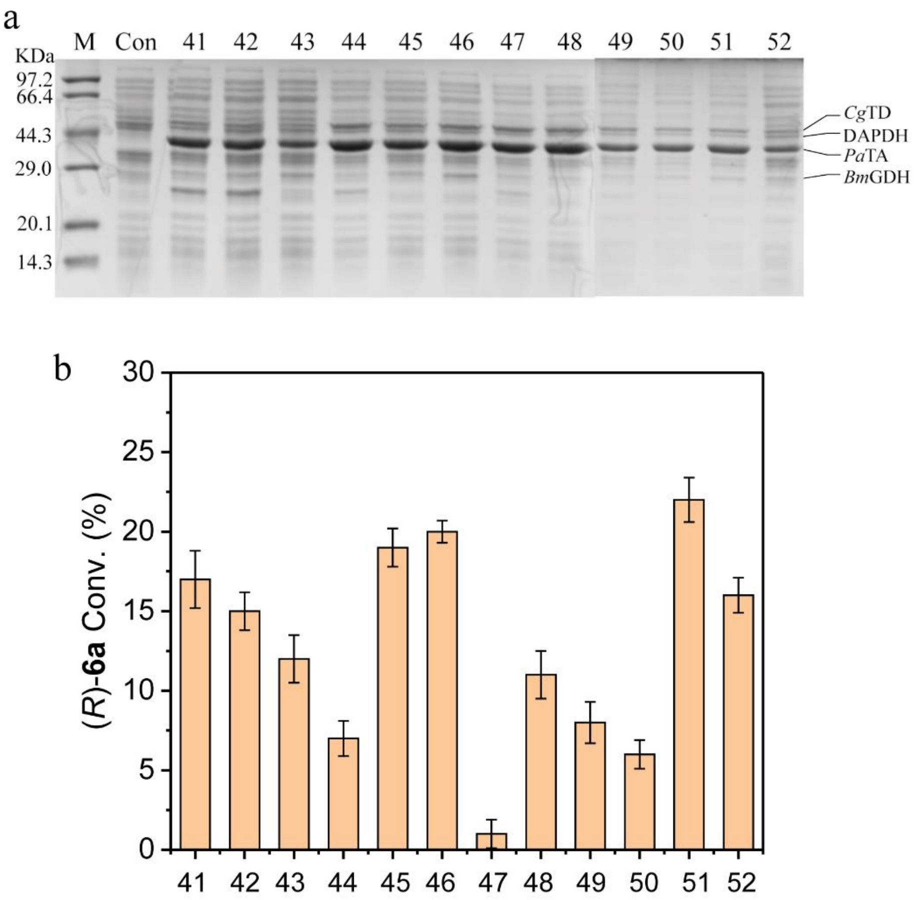

Supplementary Figure 17. Asymmetric assembly of (*R*)-**6a** from **1a** and **2** with engineered *E. coli* whole cell catalysts. (**a**) SDS-PAGE analysis of engineered *E. coli* (OA41-OA52) (M: marker; Con: *E. coli* cell without overexpressing any enzymes). (**b**) Conversion of **1a** to (*R*)-**6a** with *E. coli* (OA41-OA52). Reactions were performed with resting cells of *E. coli* (OA41-OA52) (10 g dcw l^{-1}) and **1a-i** (10 mM) in 2 ml KP buffer (50 mM, pH 8.0, 100 μM PLP, 1 mM $NADP^+$ and 10% DMSO) at 200 rpm and 25 °C for 36 h. All biotransformations were performed in triplicate, and error bars indicate ± s.d.

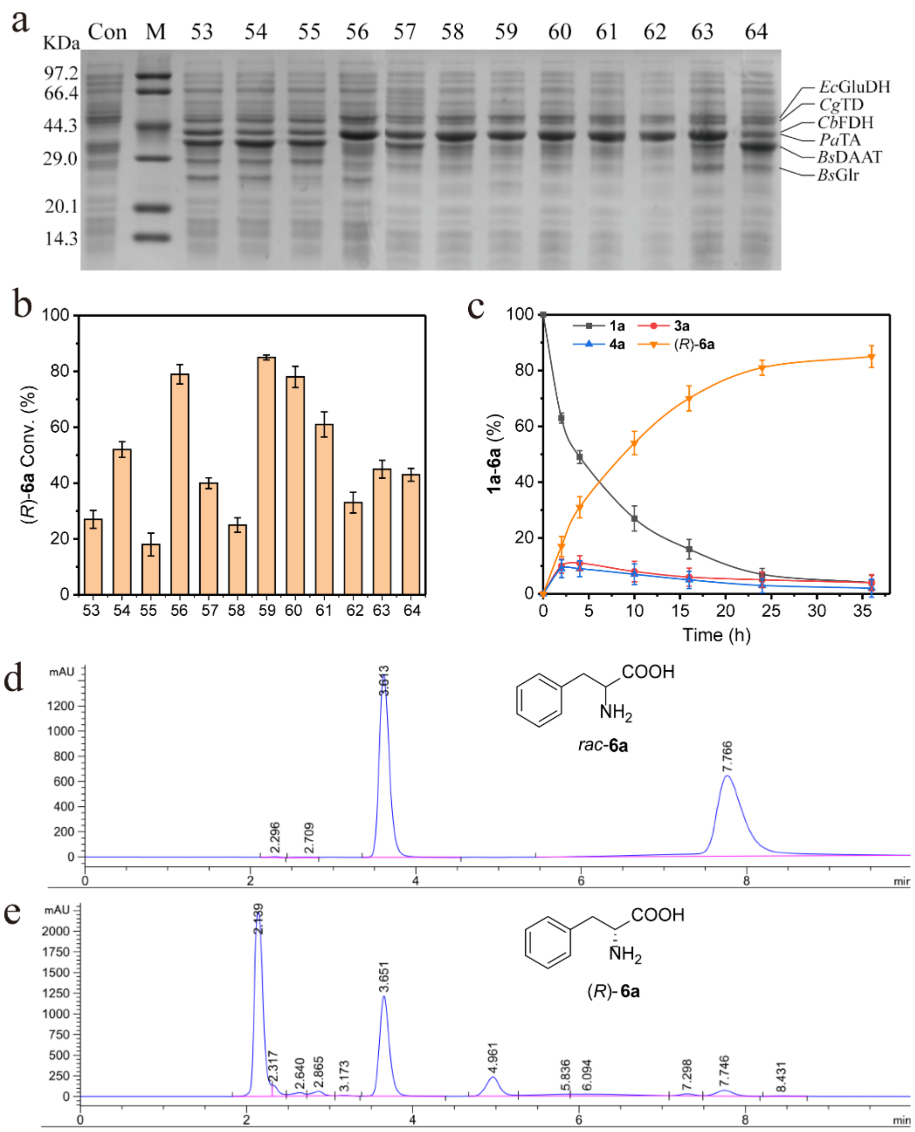

Supplementary Figure 18. Asymmetric assembly of (*R*)-**6a** from **1a** and **2** with engineered *E. coli* whole cell catalysts. (**a**) SDS-PAGE analysis of engineered *E. coli* (OA53-OA64). (**b**) Conversion of **1a** to (*R*)-**6a** with *E. coli* (OA53-OA64) (M: marker; Con: *E. coli* cell without overexpressing any enzymes). (**c**) Time course of biotransformation of **1a** to (*R*)-**6a** with *E. coli* OA59. (**d**) HPLC chromatogram of *rac*-**6a** standard. (**e**) Chiral HPLC chromatogram of sample from biotransformation of **1a** to (*R*)-**6a** with *E. coli* OA59. Reactions were performed with resting cells of *E. coli* (OA53-OA64) (10 g dcw l^{-1}) and **1a** (10 mM) in 2 ml KP buffer (50 mM, pH 8.0, 100 µM PLP, 1 mM NAD^+, and 10% DMSO) at 200 rpm and 25 ºC for 36 h. All biotransformations were performed in triplicate, and error bars indicate ± s.d.

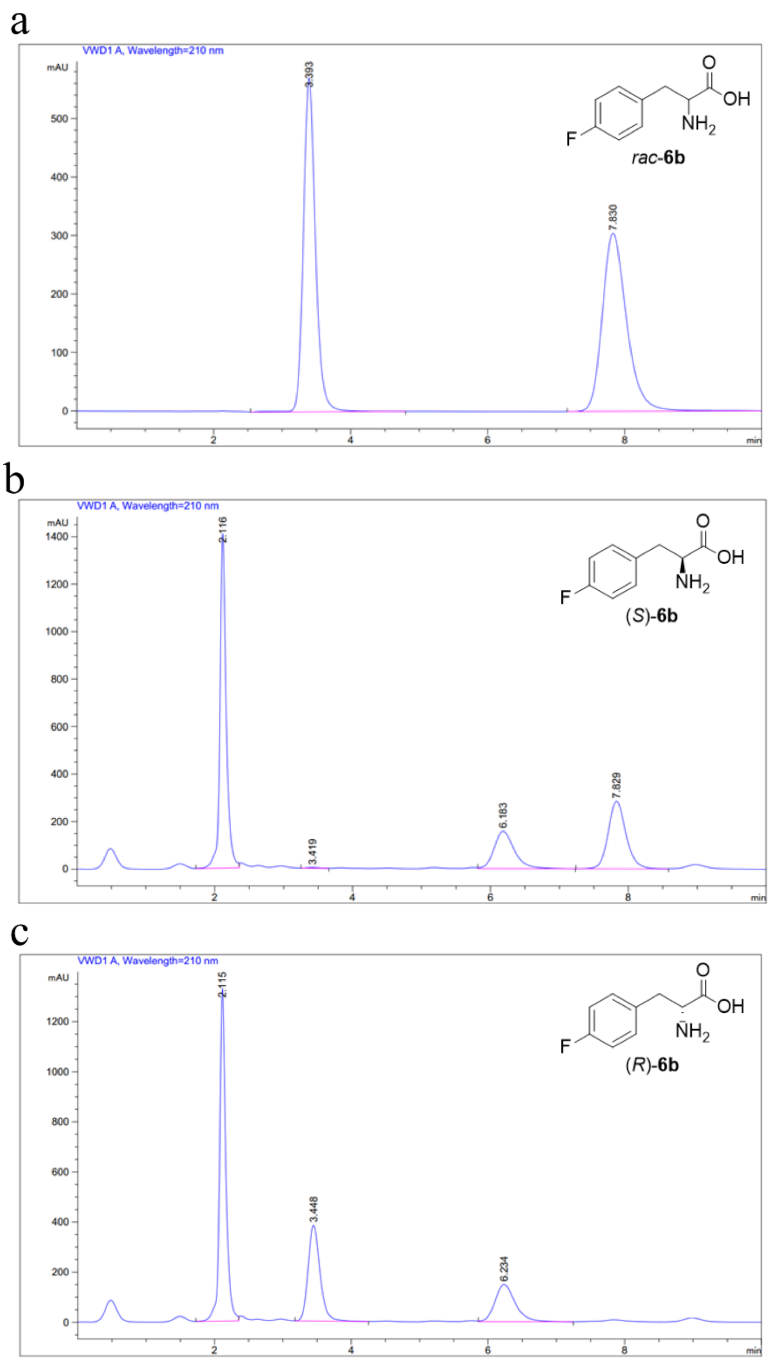

Supplementary Figure 19. Chiral HPLC chromatograms of **6b**. (**a**) Racemic **6b** standard. (**b**) Sample from biotransformation of **1b** to (*S*)-**6b**. (**c**) Sample from biotransformation of **1b** to (*R*)-**6b**.

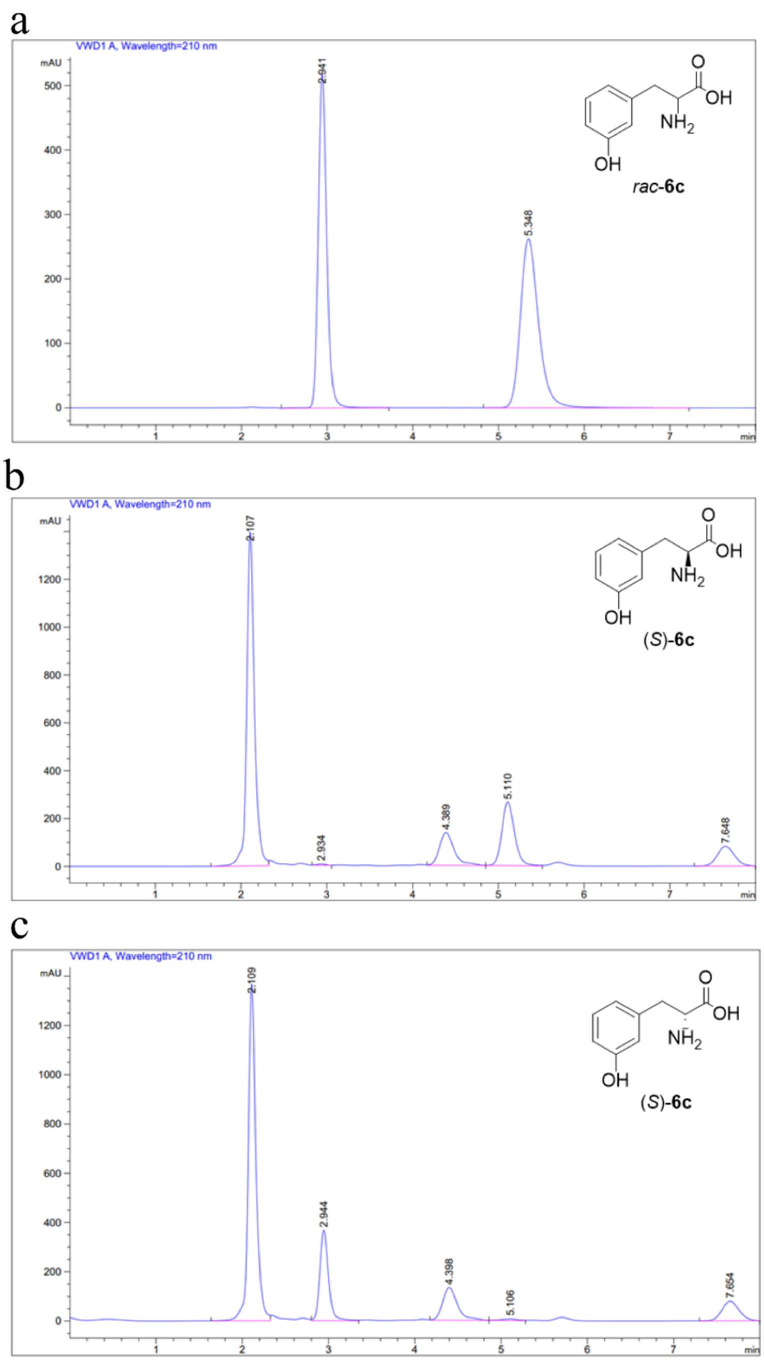

Supplementary Figure 20. Chiral HPLC chromatograms of **6c**. (**a**) Racemic **6c** standard. (**b**) Sample from biotransformation of **1c** to (*S*)-**6c**. (**c**) Sample from biotransformation of **1c** to (*R*)-**6c**.

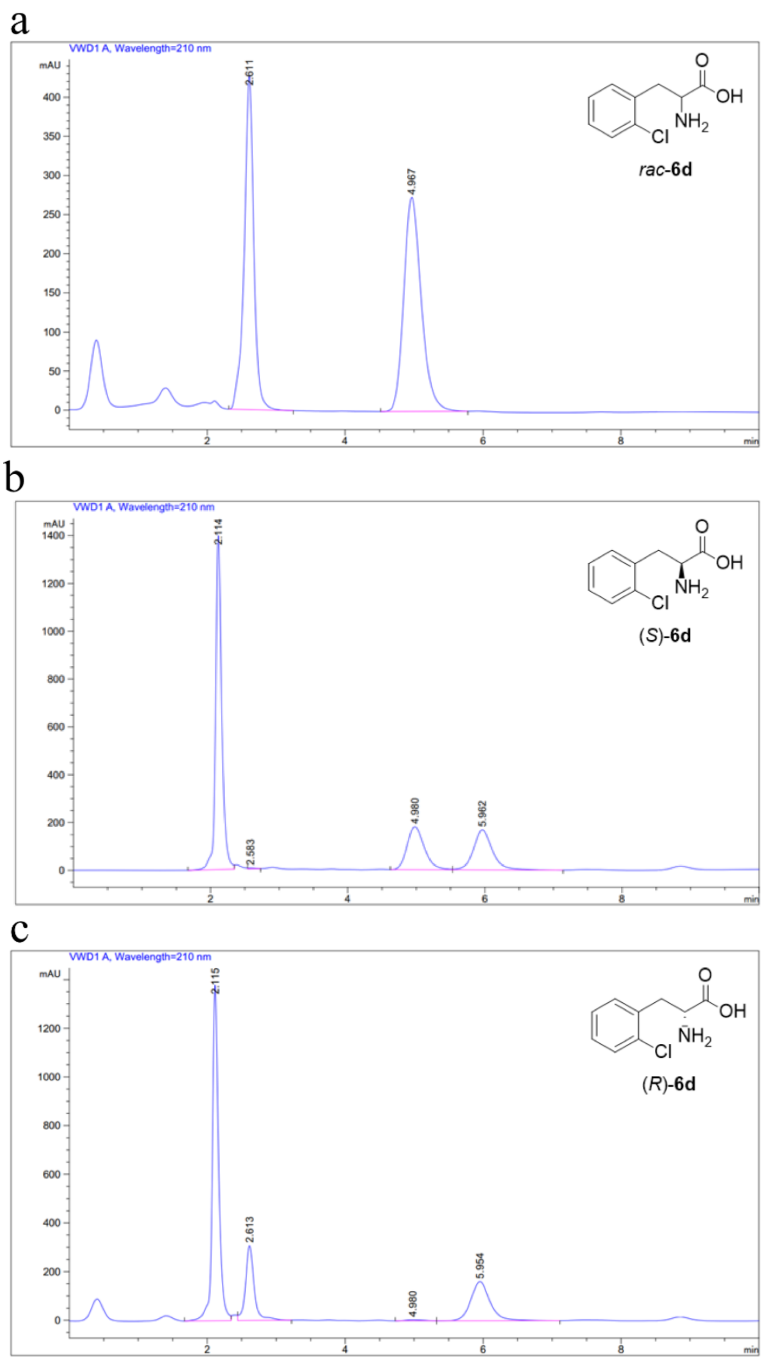

Supplementary Figure 21. Chiral HPLC chromatograms of **6d**. (**a**) Racemic **6d** standard. (**b**) Sample from biotransformation of **1d** to (*S*)-**6d**. (**c**) Sample from biotransformation of **1d** to (*R*)-**6d**.

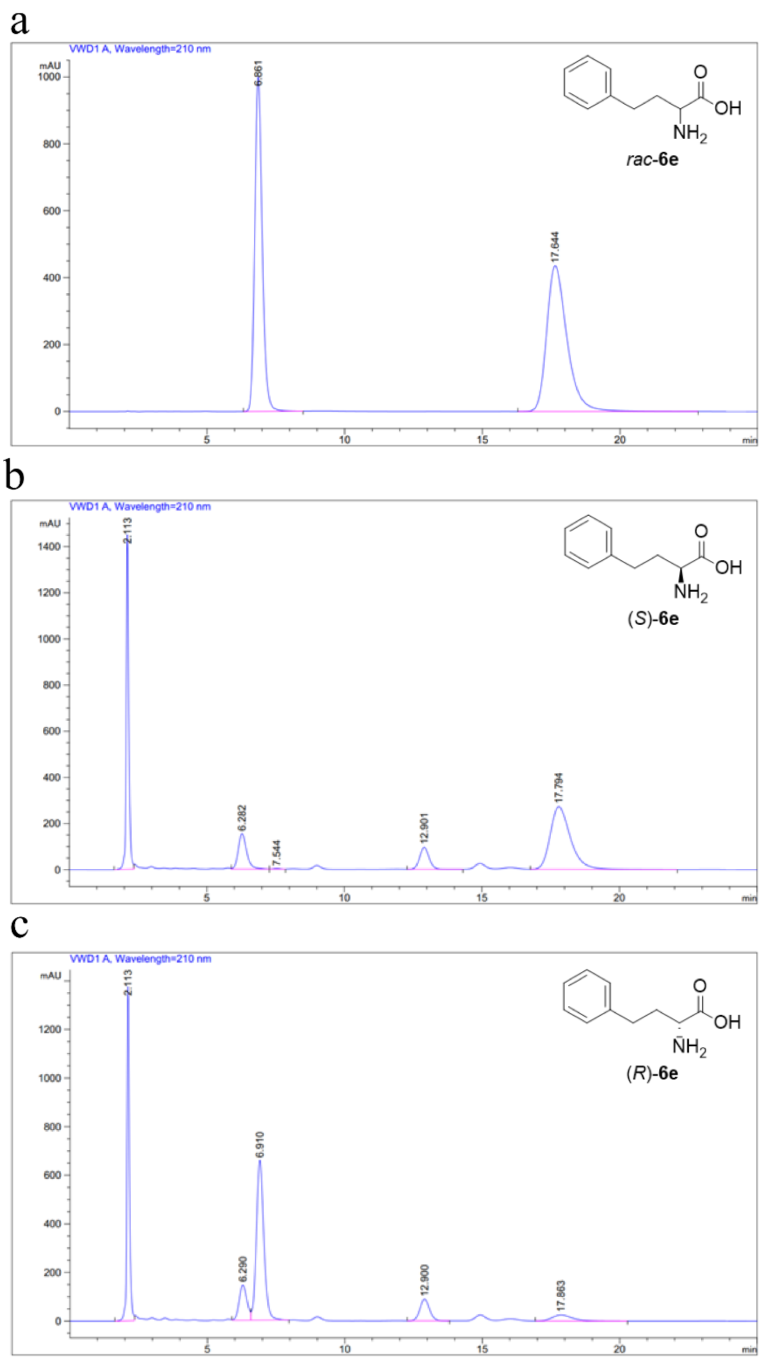

Supplementary Figure 22. Chiral HPLC chromatograms of **6e**. (**a**) Racemic **6e** standard. (**b**) Sample from biotransformation of **1e** to (*S*)-**6e**. (**c**) Sample from biotransformation of **1e** to (*R*)-**6e**.

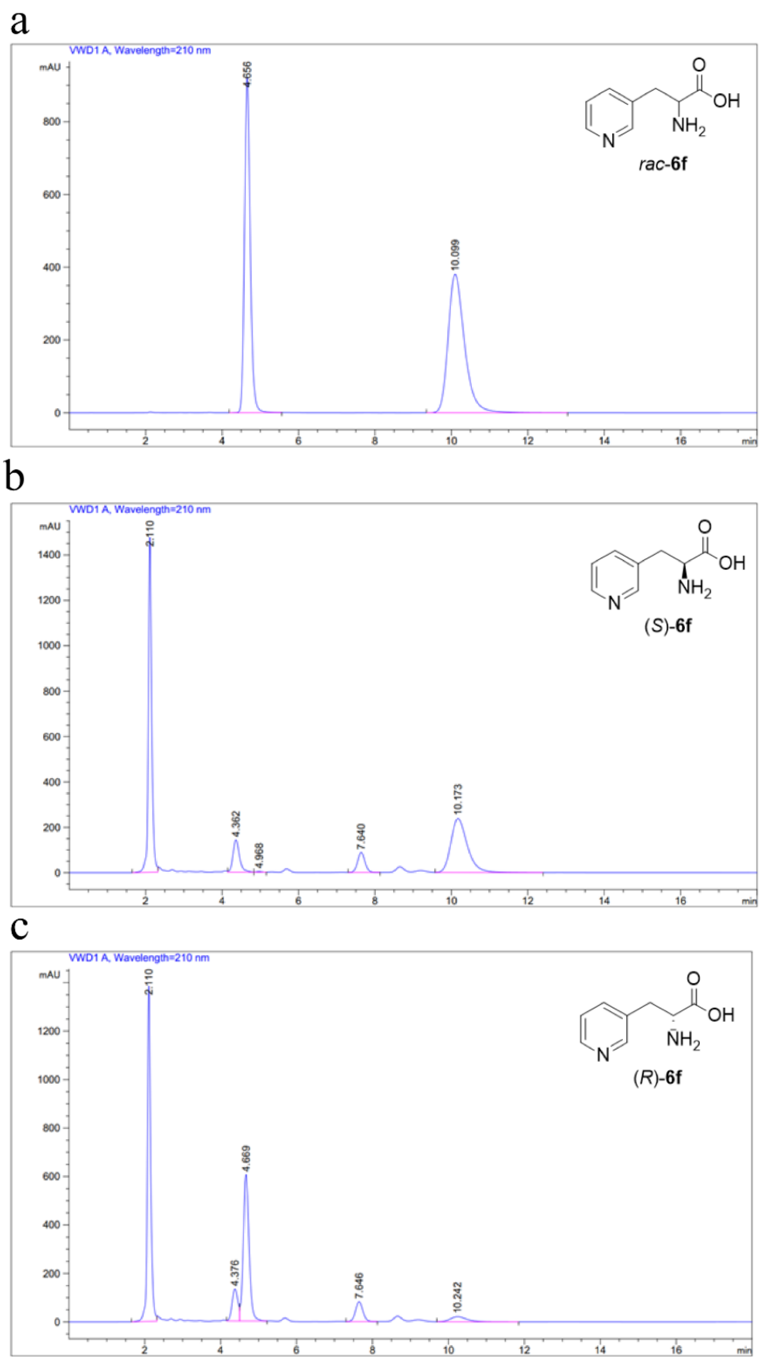

Supplementary Figure 23. Chiral HPLC chromatograms of **6f**. (**a**) Racemic **6f** standard. (**b**) Sample from biotransformation of **1f** to (*S*)-**6f**. (**c**) Sample from biotransformation of **1f** to (*R*)-**6f**.

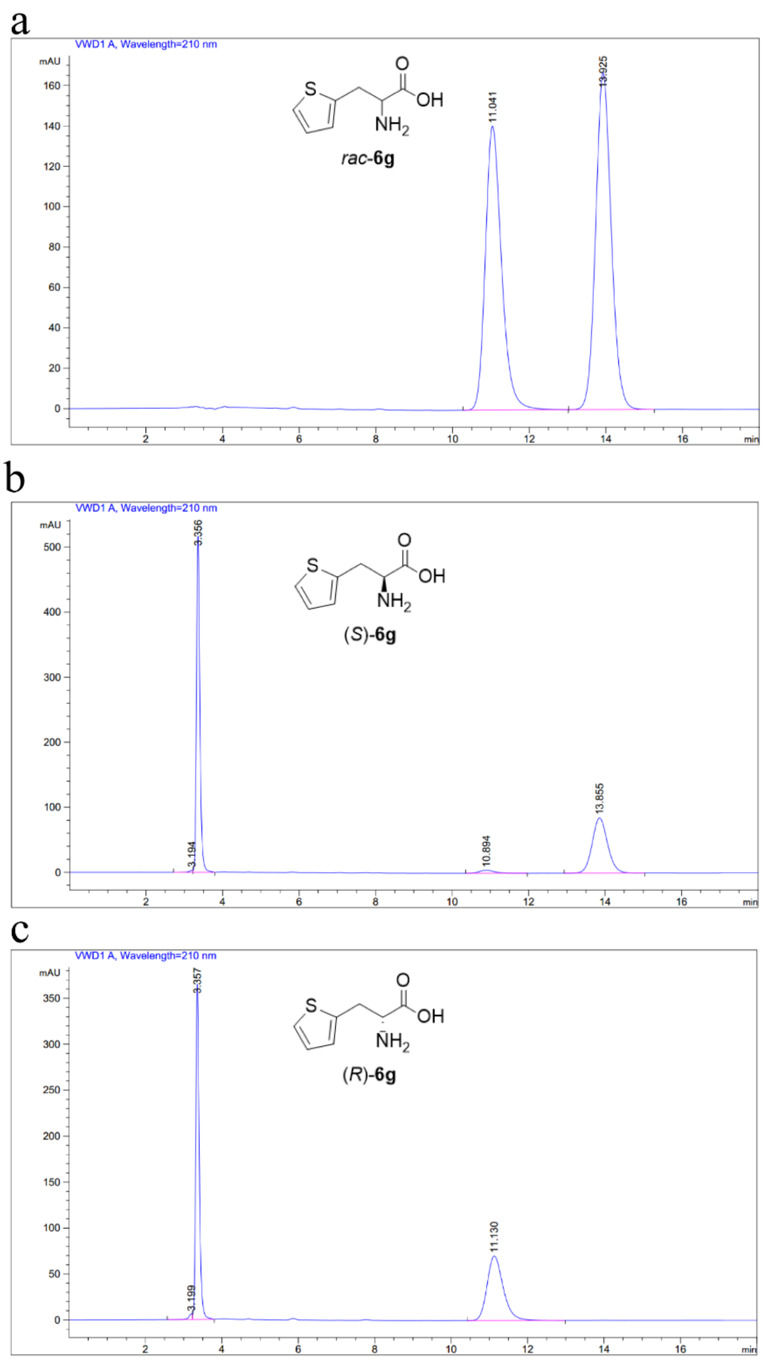

Supplementary Figure 24. Chiral HPLC chromatograms of **6g**. (**a**) Racemic **6g** standard. (**b**) Sample from biotransformation of **1g** to (*S*)-**6f**. (**c**) Sample from biotransformation of **1g** to (*R*)-**6g**.

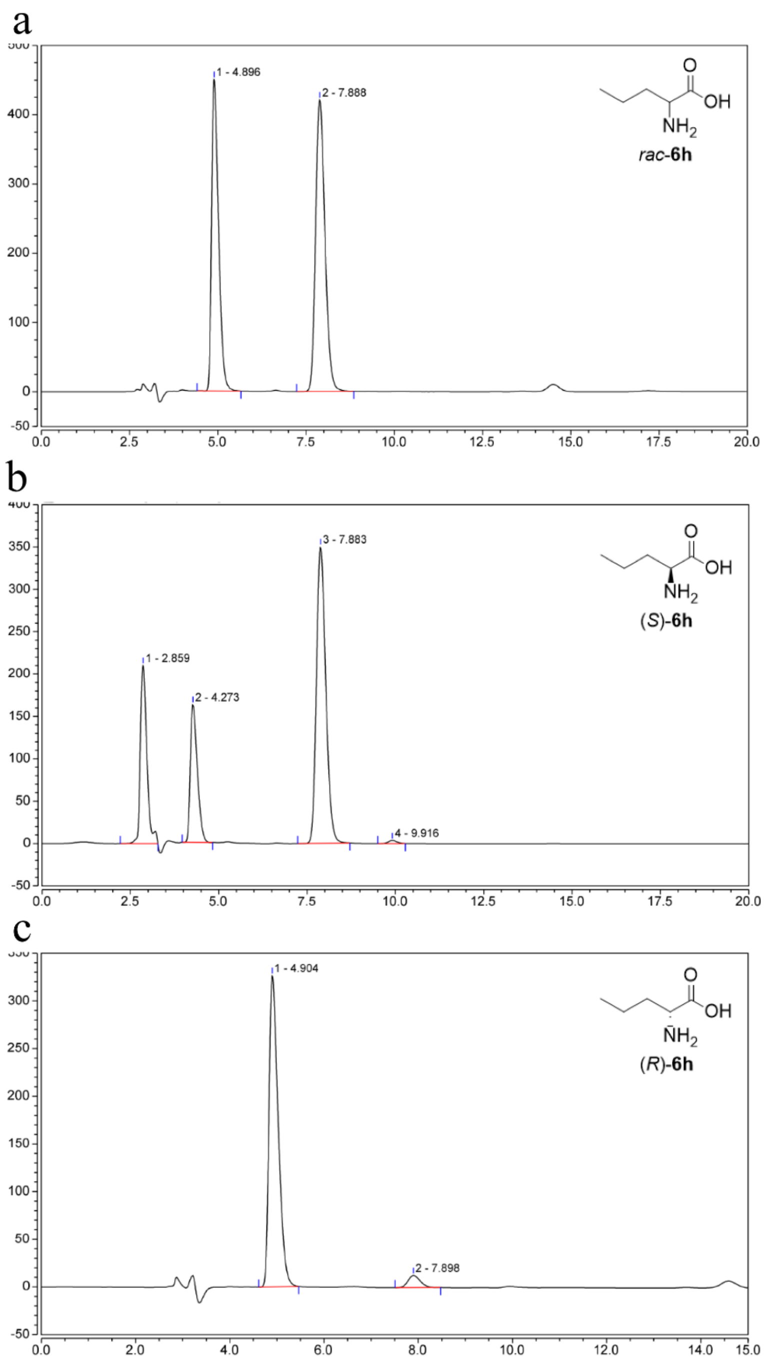

Supplementary Figure 25. Chiral HPLC chromatograms of **6h**. (**a**) Racemic **6h** standard. (**b**) Sample from biotransformation of **1h** to (*S*)-**6h**. (**c**) Sample from biotransformation of **1h** to (*R*)-**6h**.

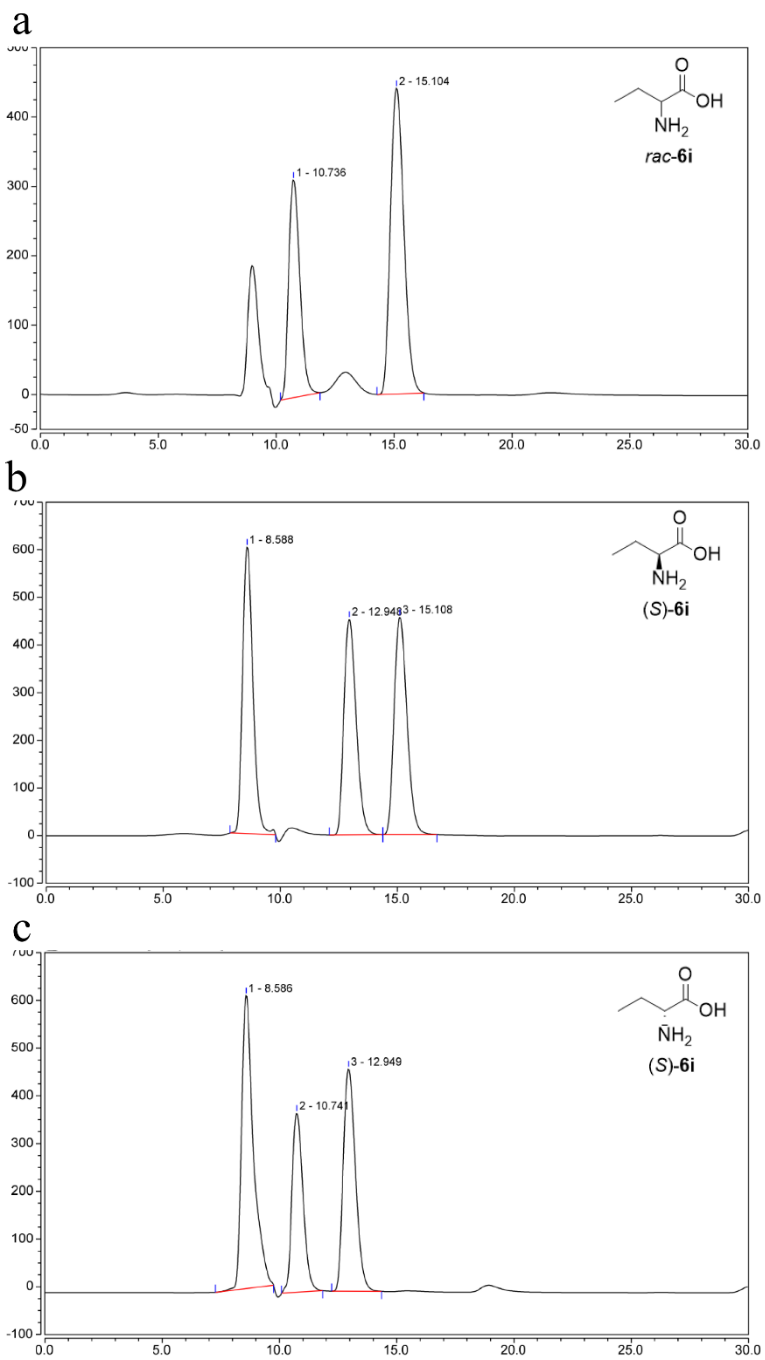

Supplementary Figure 26. Chiral HPLC chromatograms of **6i**. (**a**) Racemic **6i** standard. (**b**) Sample from biotransformation of **1i** to (*S*)-**6i**. (**c**) Sample from biotransformation of **1i** to (*R*)-**6i**.

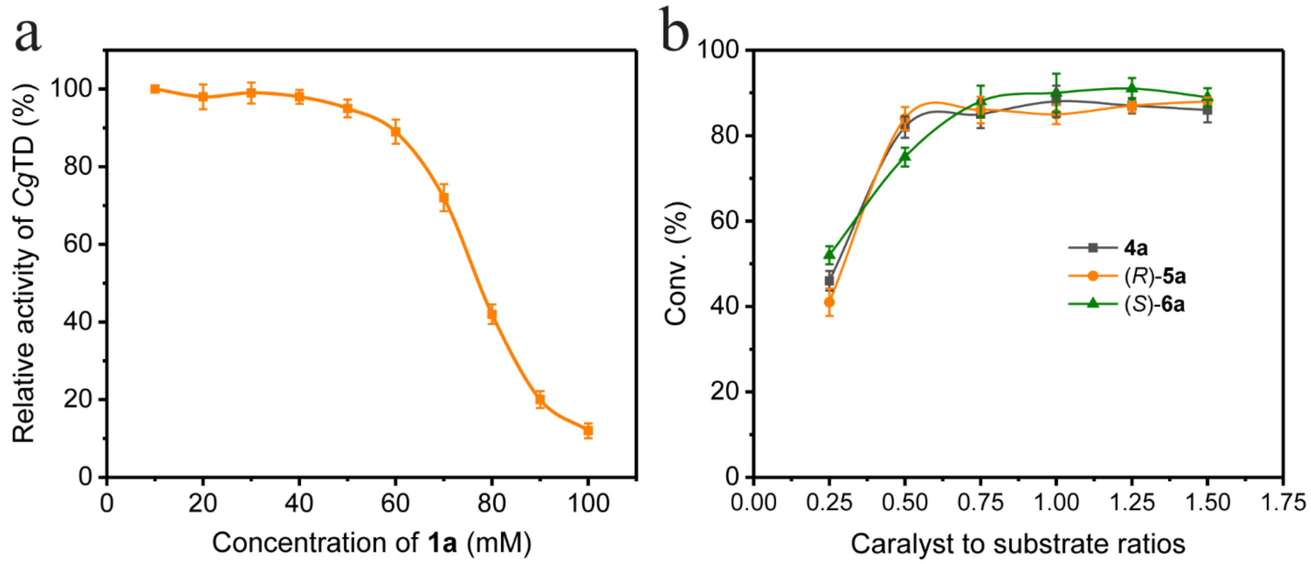

Supplementary Figure 27. Optimization of biotransformation conditions for the scaled-up biotransformations. (**a**) The tolerance of the key enzyme *Cg*TD to **1a**. (**b**) The ratios of the catalyst to substrate (from 0.25-1.75:1 g dcw l^{-1}/mM substrate, with 50 mM **1a**). The DMSO concentration was examined for the biotransformation of **1a** to **4a**, and 5%-15% (v/v) gave similar high conversion for **4a** (Fig. S6e). Thus, the preparative biotransformations were performed 5% DMSO to reduce the use of organic solvent. Optimized conditions: whole-cell catalyst (30 g dcw l^{-1}) and 50mM substrate in KP buffer (50 mM, pH 8.0, 100 µM PLP, and 5% DMSO) at 200 rpm and 25 ºC for 30-42 h. All experiments were performed in triplicate, and error bars indicate ± s.d.

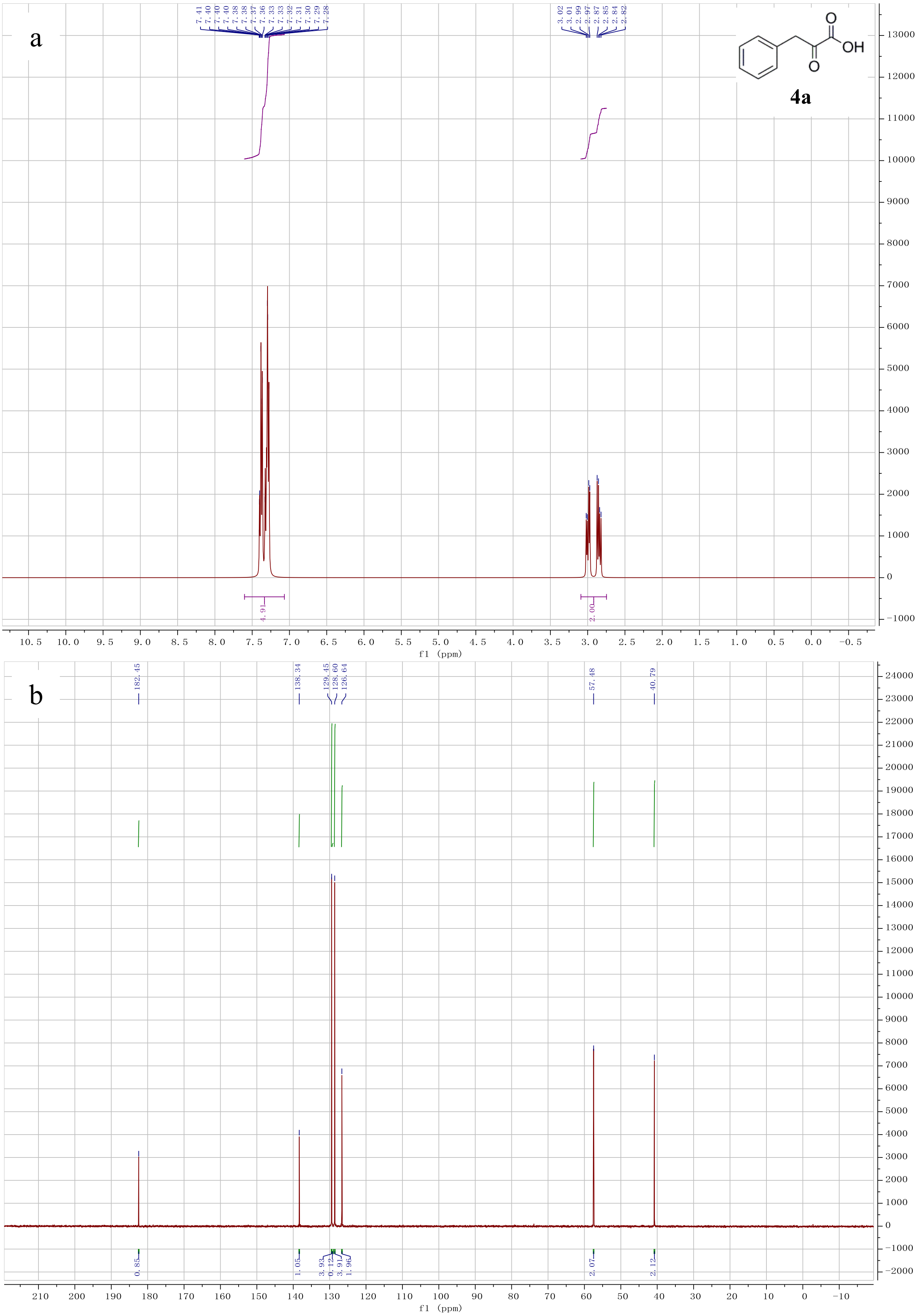

Supplementary Figure 28. NMR spectra of **4a**. (**a**) ^{1}H-NMR spectra of **4a**. (**b**) ^{13}C-NMR spectra of **4a**.

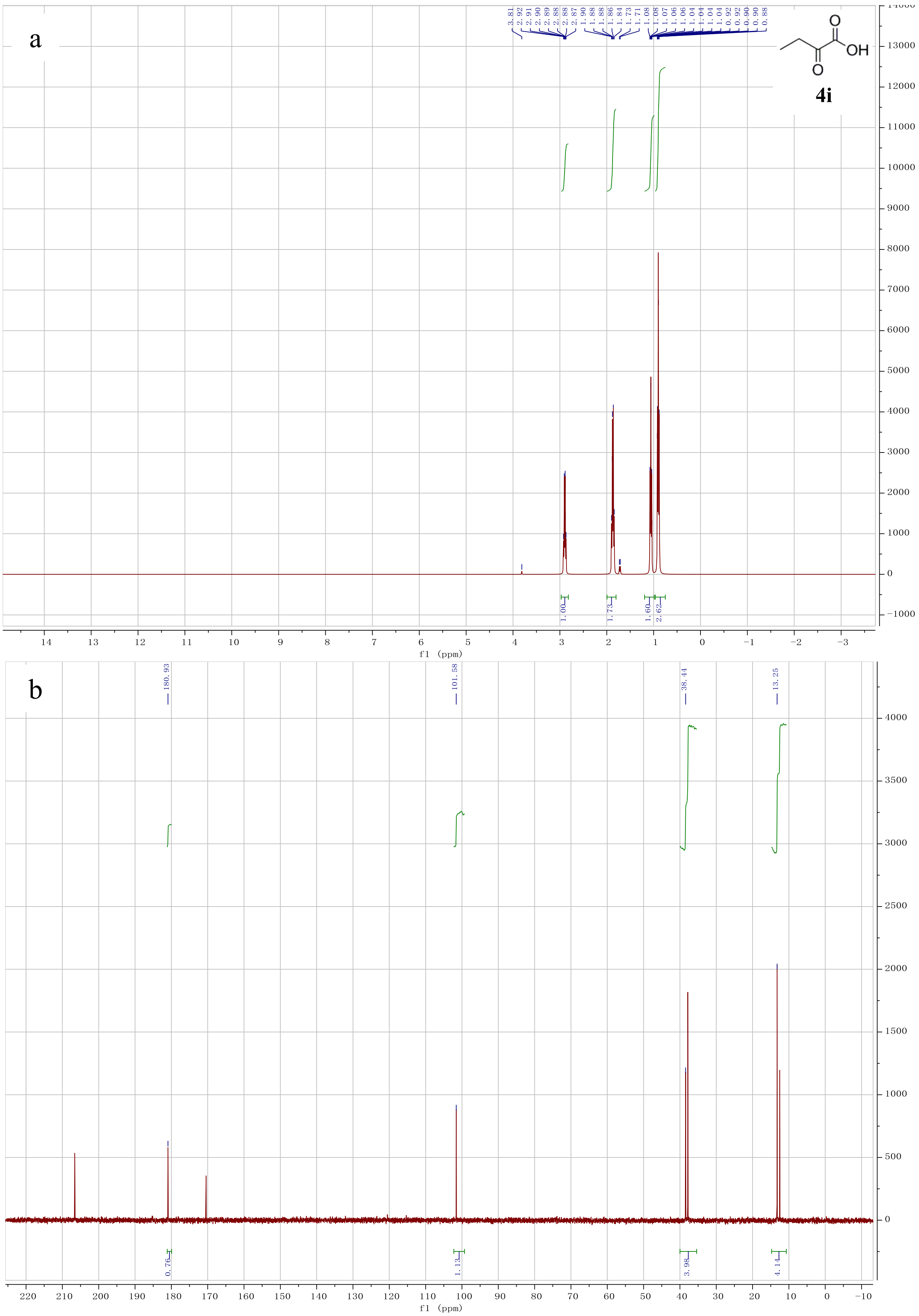

Supplementary Figure 29. NMR spectra of **4i**. (**a**) ^{1}H-NMR spectra of **4i**. (**b**) ^{13}C-NMR spectra of **4i**.

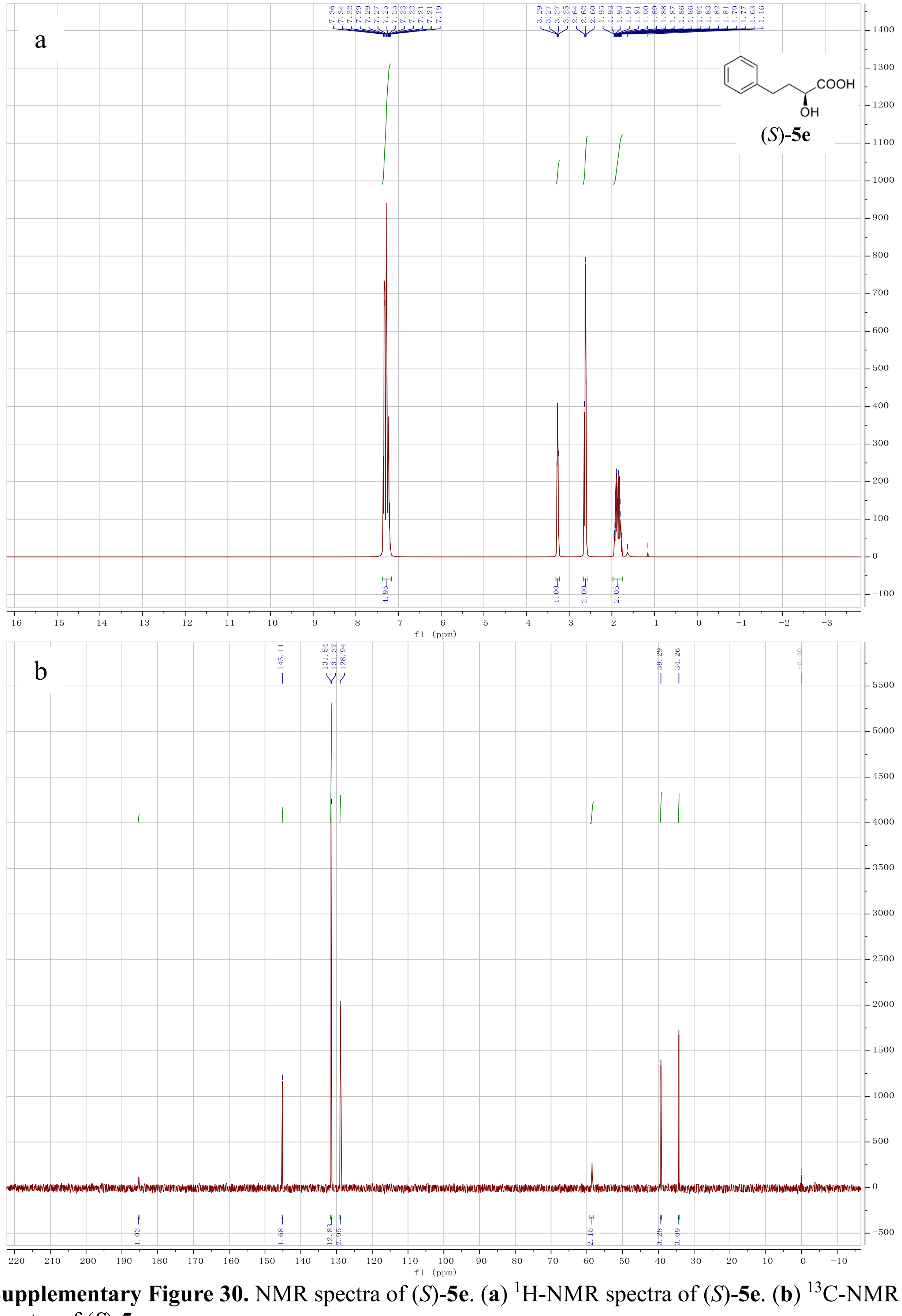

Supplementary Figure 30. NMR spectra of (*S*)-**5e**. (**a**) ^{1}H-NMR spectra of (*S*)-**5e**. (**b**) ^{13}C-NMR spectra of (*S*)-**5e**.

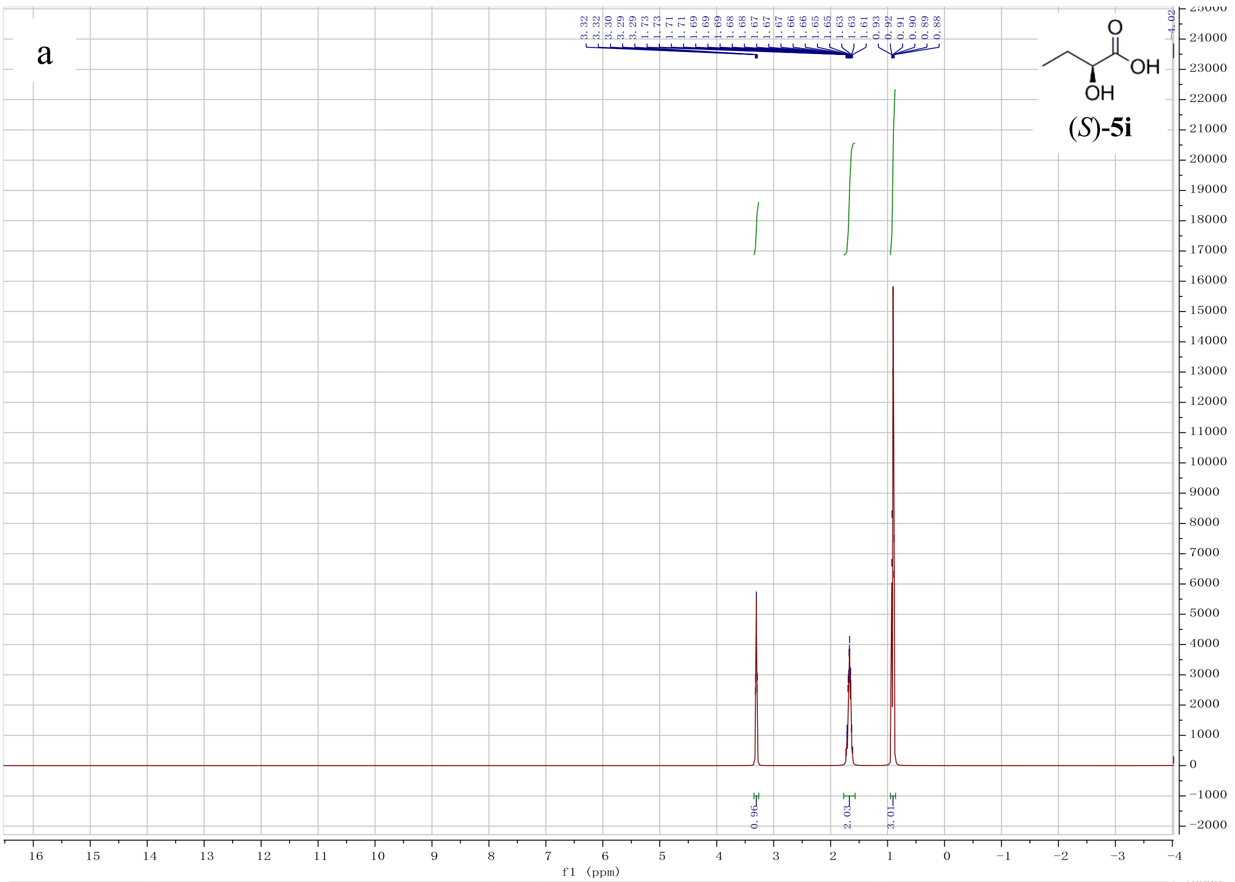

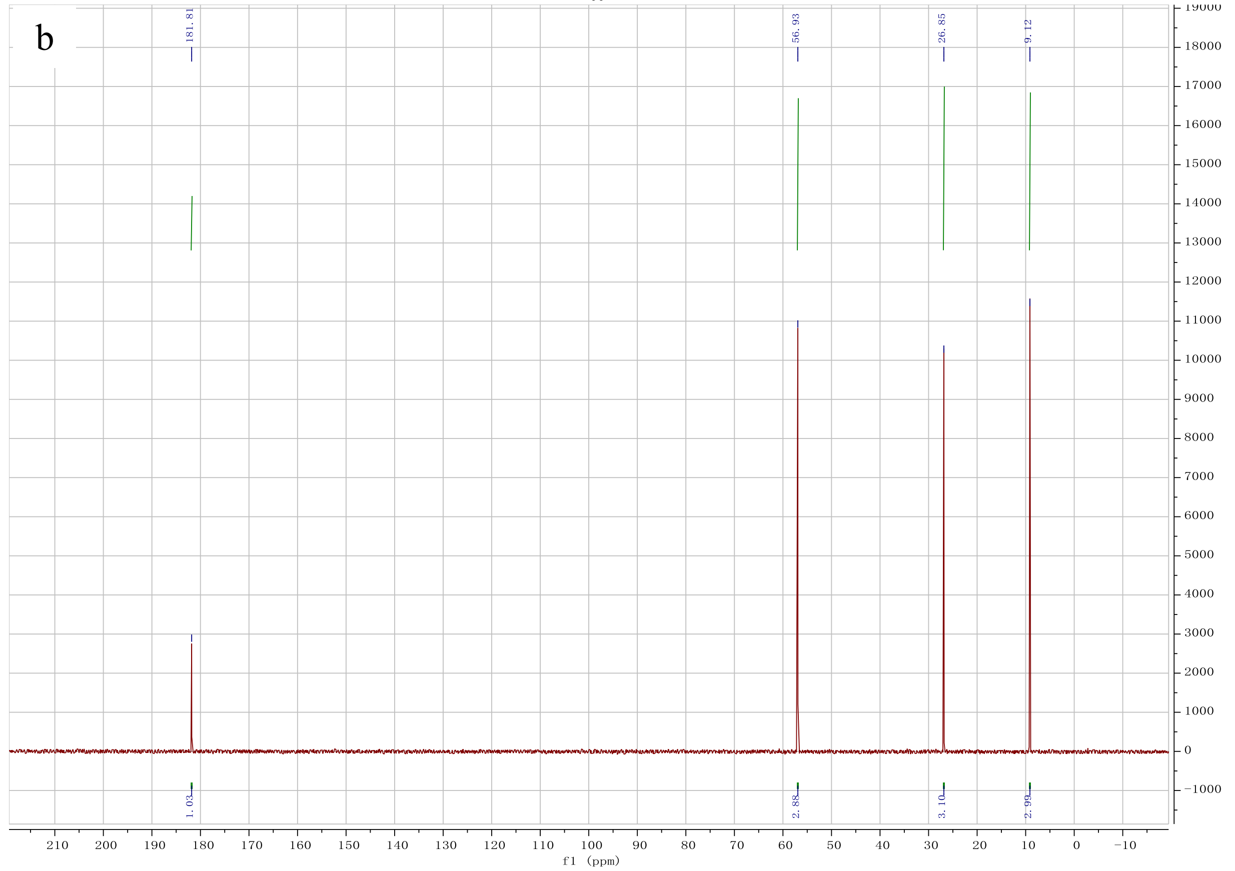

Supplementary Figure 31. NMR spectra of (*S*)-**5i**. (**a**) ^{1}H-NMR spectra of (*S*)-**5i**. (**b**) ^{13}C-NMR spectra of (*S*)-**5i**.

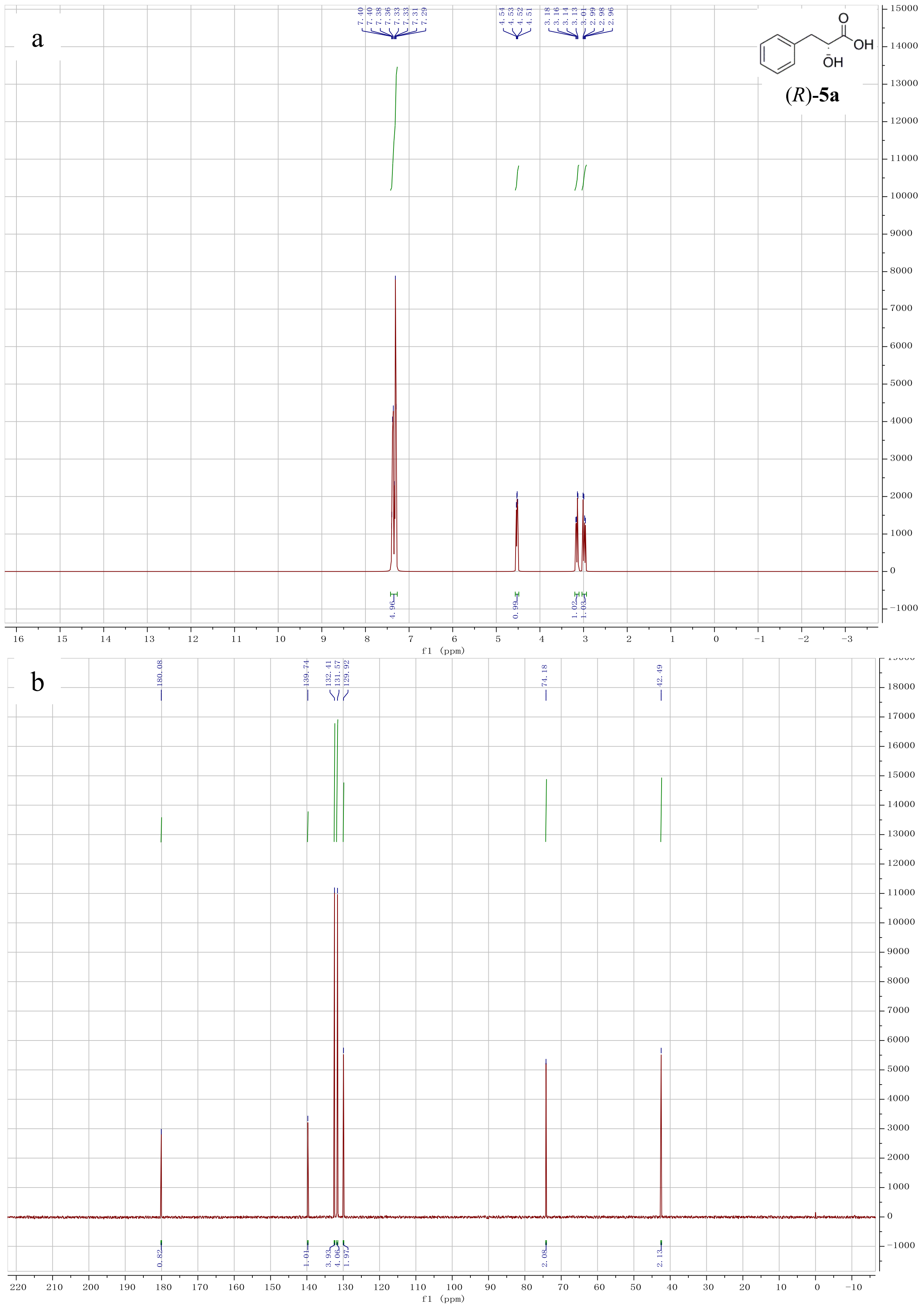

Supplementary Figure 32. NMR spectra of (*R*)-**5a**. (**a**) ^{1}H-NMR spectra of (*R*)-**5a**. (**b**) ^{13}C-NMR spectra of (*R*)-**5a**.

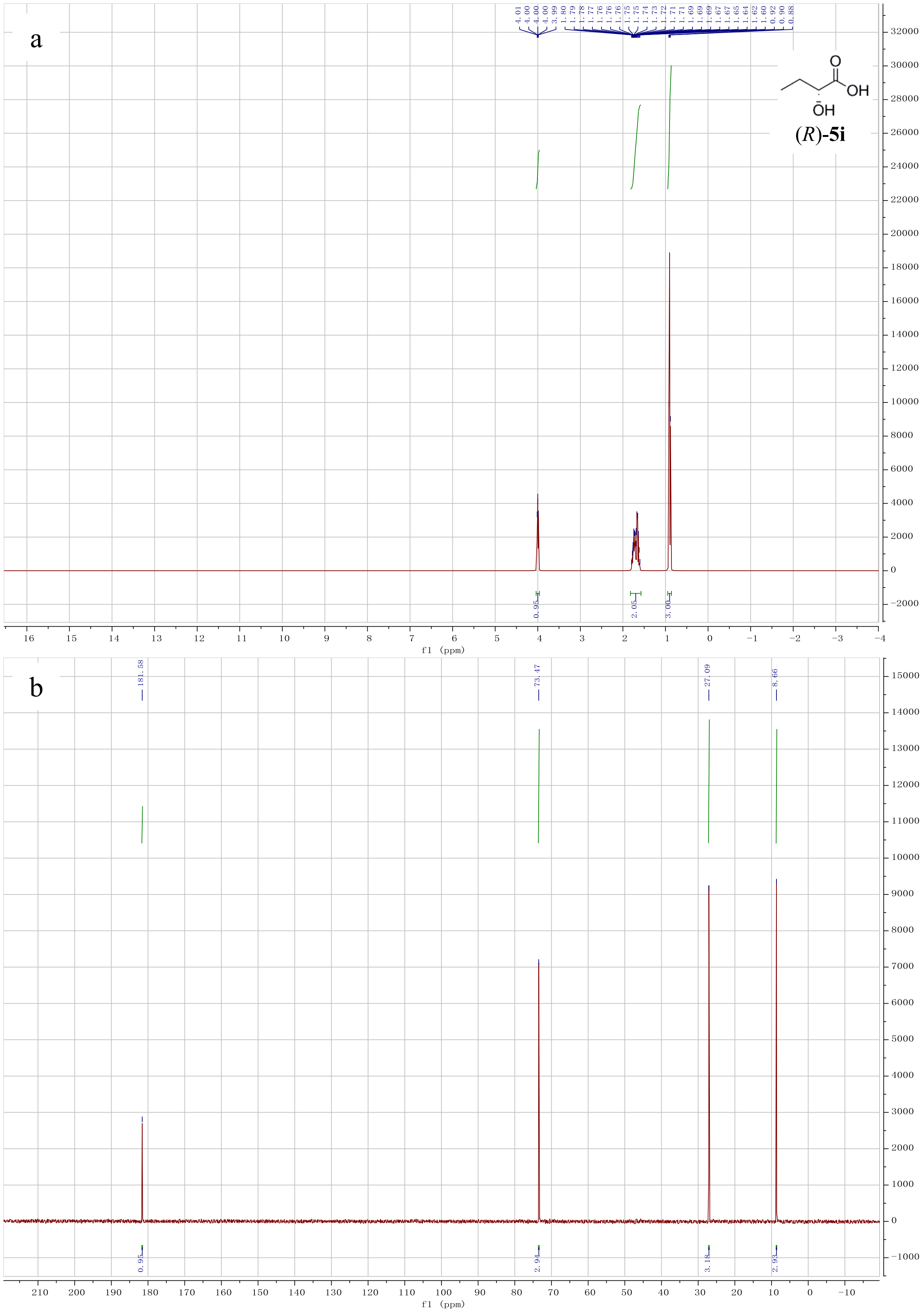

Supplementary Figure 33. NMR spectra of (*R*)-**5i**. (**a**) ^{1}H-NMR spectra of (*R*)-**5i**. (**b**) ^{13}C-NMR spectra of (*R*)-**5i**.

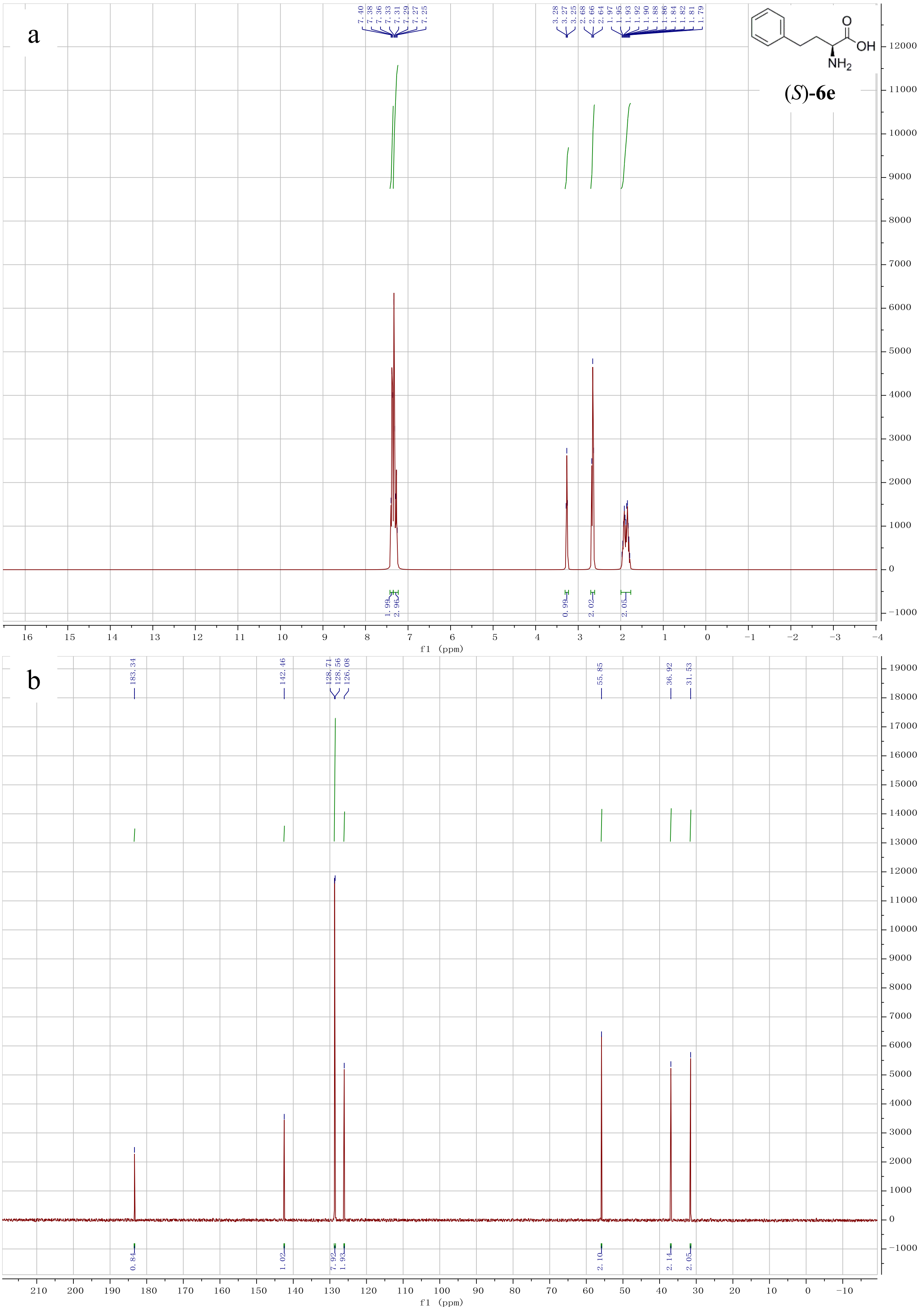

Supplementary Figure 34. NMR spectra of (*S*)-**6e**. (**a**) ^{1}H-NMR spectra of (*S*)-**6e**. (**b**) ^{13}C-NMR spectra of (*S*)-**6e**.

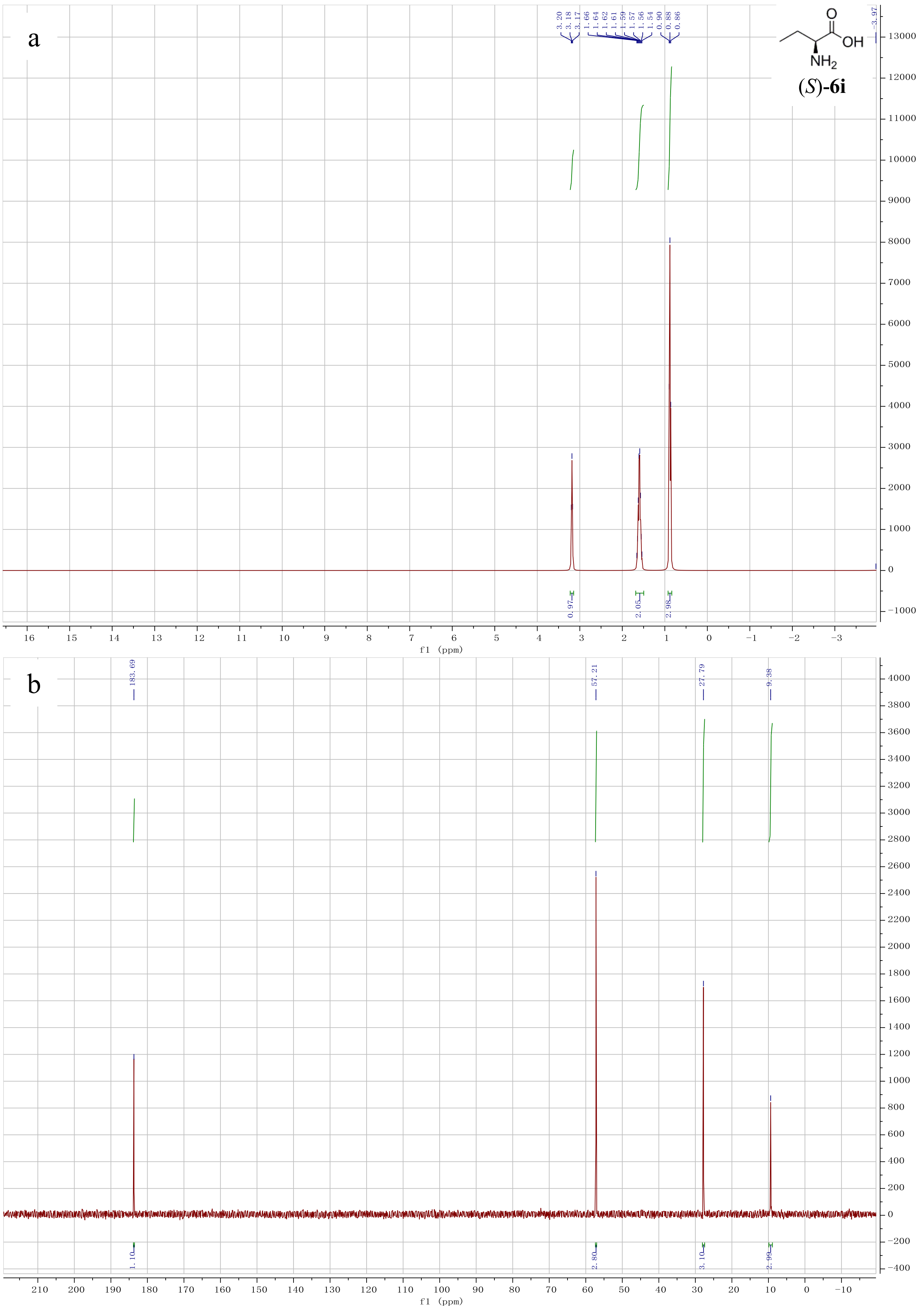

Supplementary Figure 35. NMR spectra of (*S*)-**6i**. (**a**) ^{1}H-NMR spectra of (*S*)-**6i**. (**b**) ^{13}C-NMR spectra of (*S*)-**6i**.

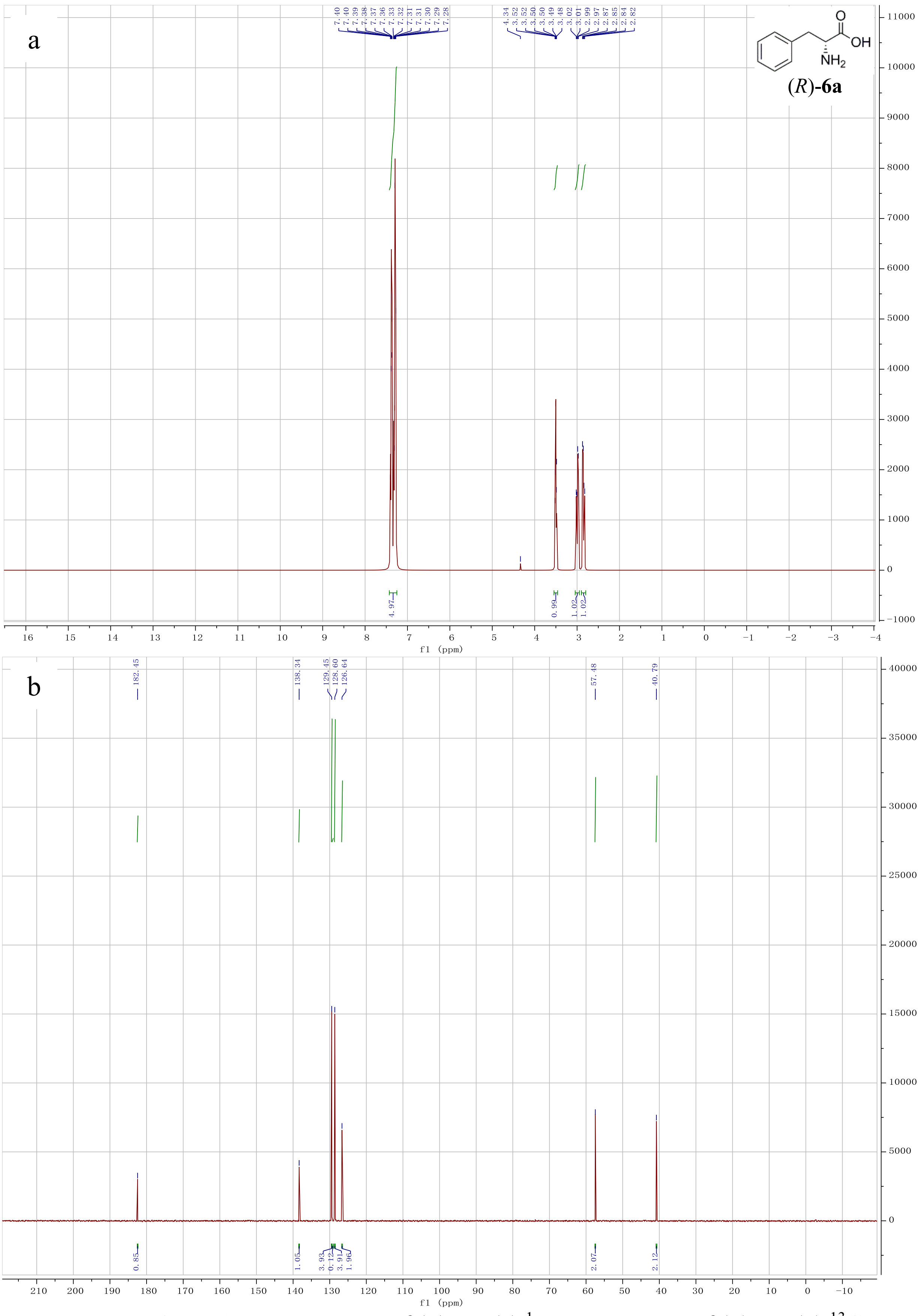

Supplementary Figure 36. NMR spectra of (*R*)-**6a**. (**a**) ^{1}H-NMR spectra of (*R*)-**6a**. (**b**) ^{13}C-NMR spectra of (*R*)-**6a**.

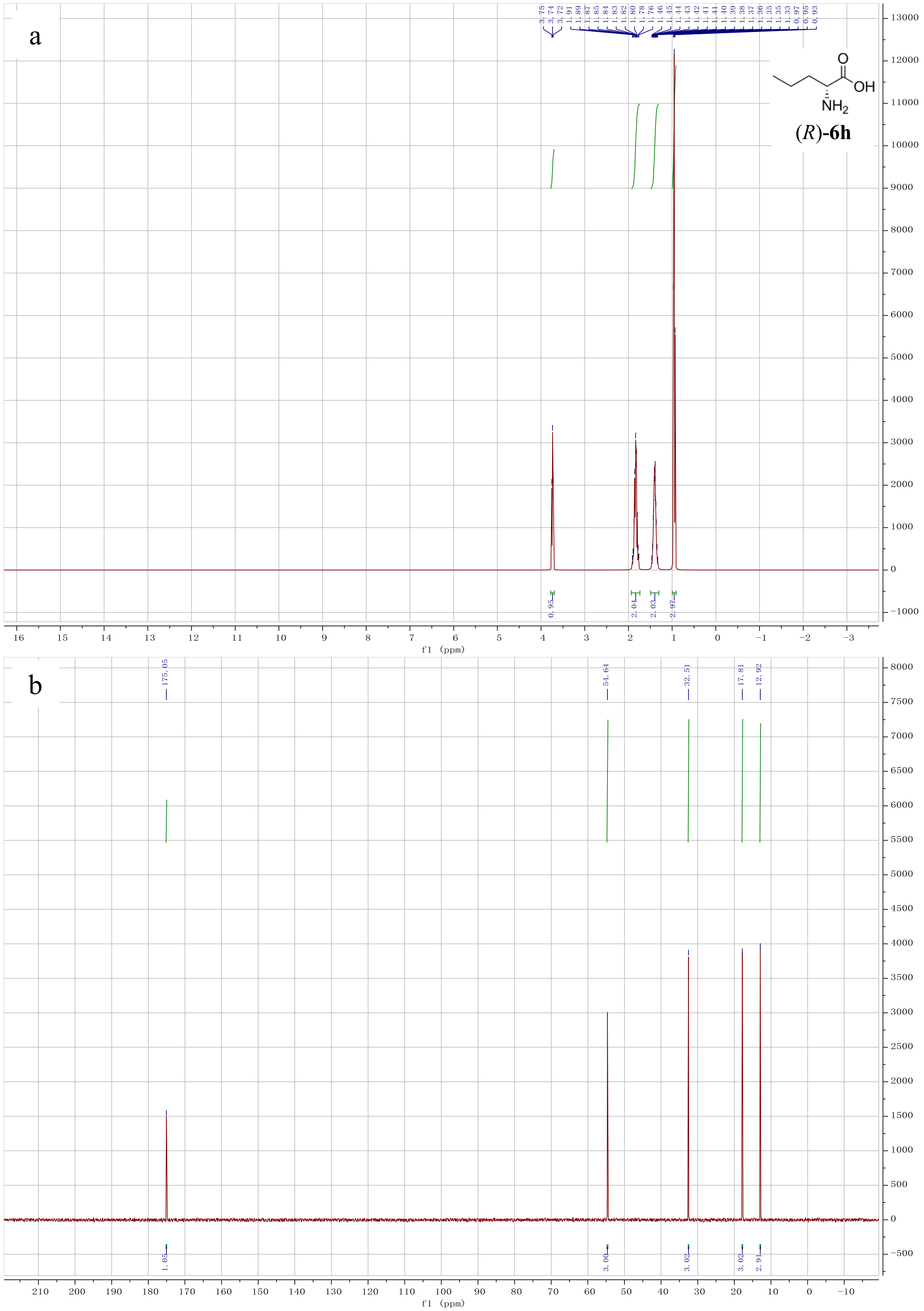

Supplementary Figure 37. NMR spectra of (*R*)-**6h**. (**a**) ^{1}H-NMR spectra of (*R*)-**6h**. (**b**) ^{13}C-NMR spectra of (*R*)-**6h**.

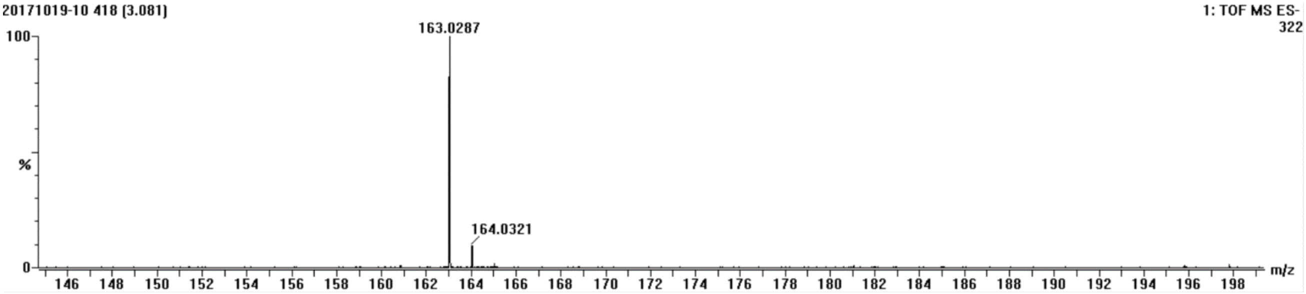

Supplementary Figure 38. HRMS spectrum of **4a**. (ESI$^-$) *m/z*: $C_9H_8O_3$ [M-H]$^-$, 163.0287.

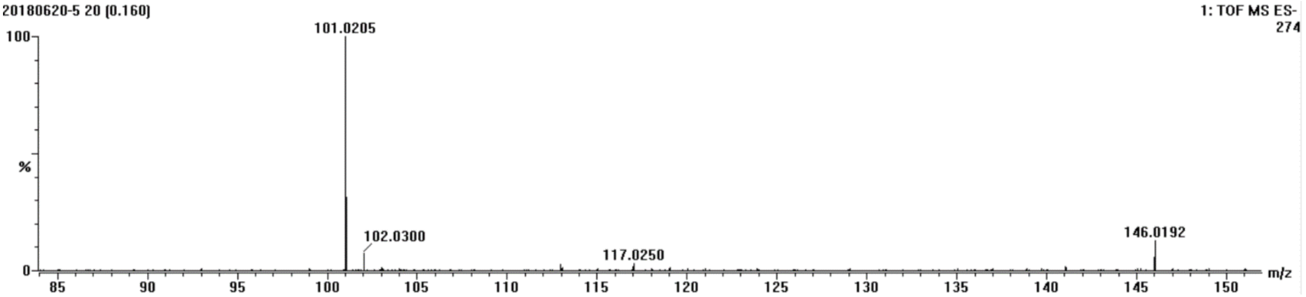

Supplementary Figure 39. HRMS spectrum of **4i**. (ESI$^-$) *m/z*: $C_4H_6O_3$ [M-H]$^-$, 101.0205.

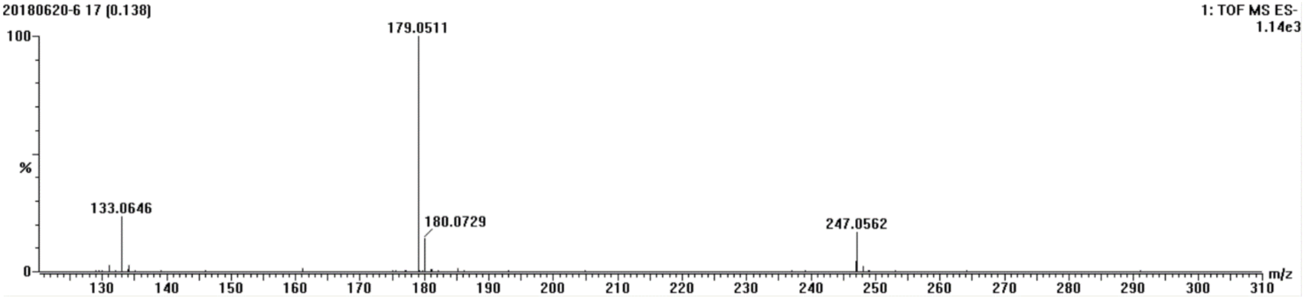

Supplementary Figure 40. HRMS spectrum of (*S*)-**5e**. (ESI$^-$) *m/z*: $C_{10}H_{12}O_3$ [M-H]$^-$, 179.0511.

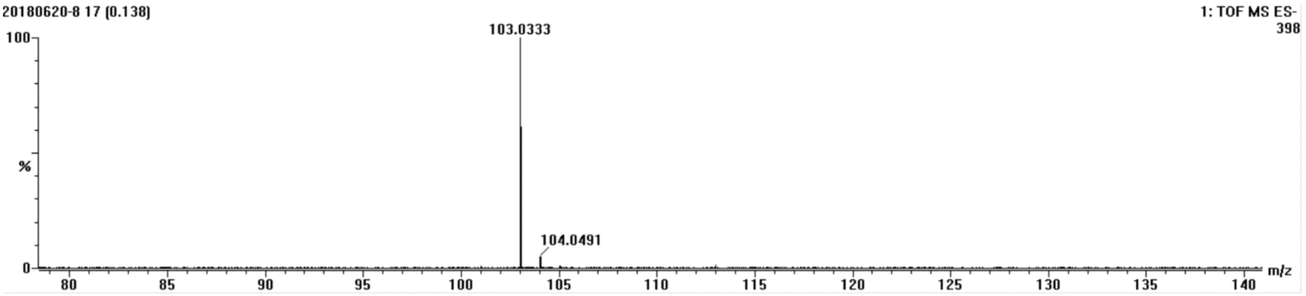

Supplementary Figure 41. HRMS spectrum of (*S*)-**5i**. (ESI$^-$) *m/z*: $C_4H_8O_3$ [M-H]$^-$, 103.0333.

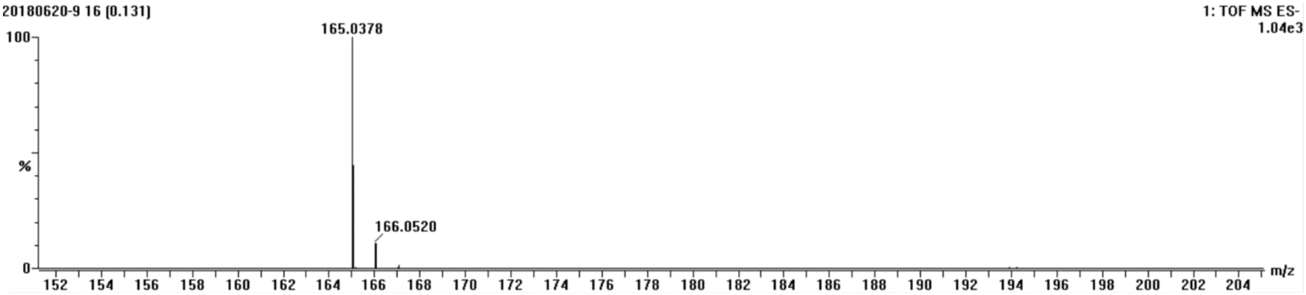

Supplementary Figure 42. HRMS spectrum of (*R*)-**5a**. (ESI$^-$) *m/z*: $C_9H_{10}O_3$ [M-H]$^-$, 165.0378.

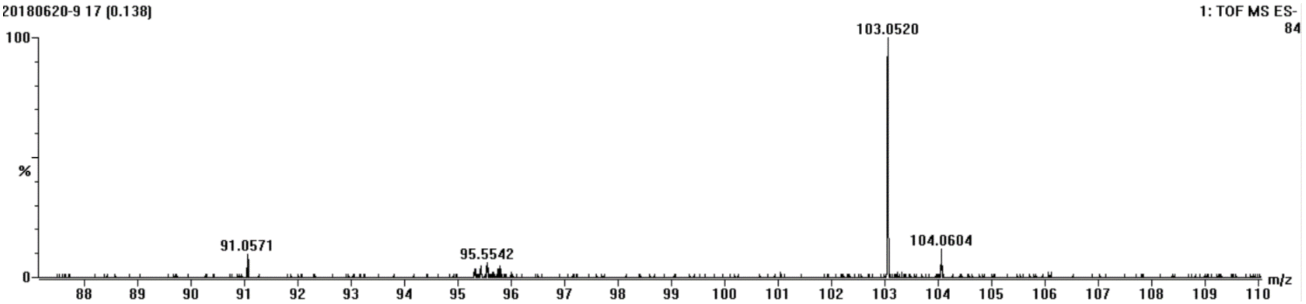

Supplementary Figure 43. HRMS spectrum of (*R*)-**5i**. (ESI$^-$) *m/z*: $C_4H_8O_3$ [M-H]$^-$, 103.0520.

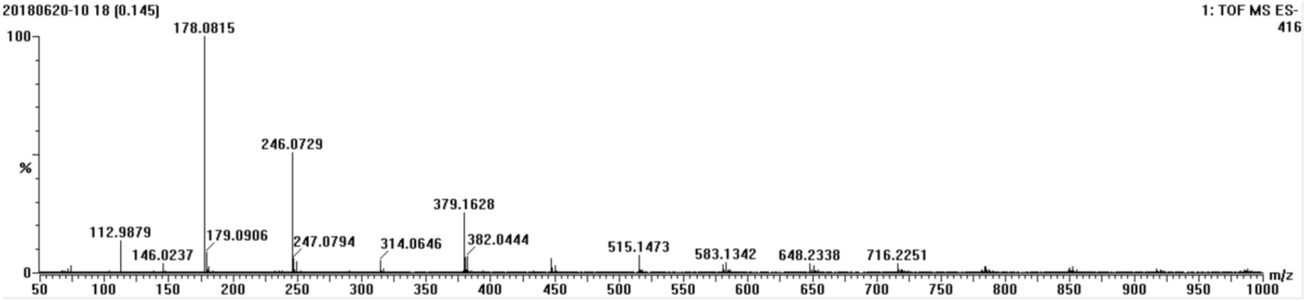

Supplementary Figure 44. HRMS spectrum of (*S*)-**6e**. (ESI$^-$) *m/z*: $C_{10}H_{13}NO_2$ [M-H]$^-$, 178.0815.

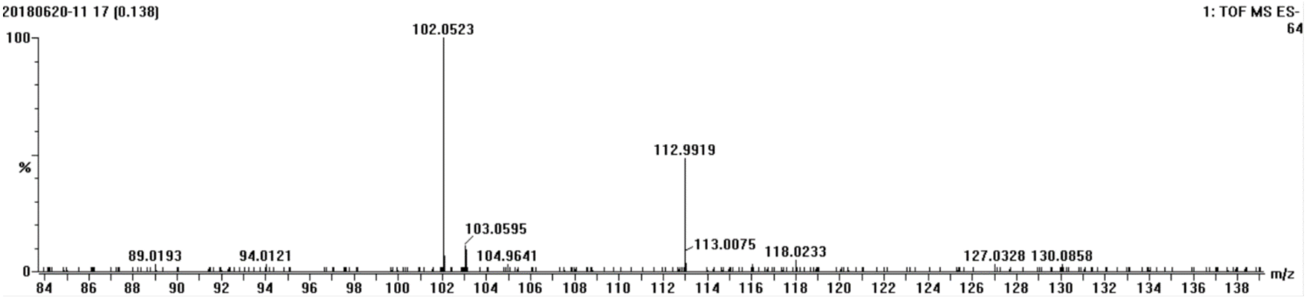

Supplementary Figure 45. HRMS spectrum of (*S*)-**6i**. (ESI$^-$) *m/z*: $C_4H_9NO_2$ [M-H]$^-$, 102.0523.

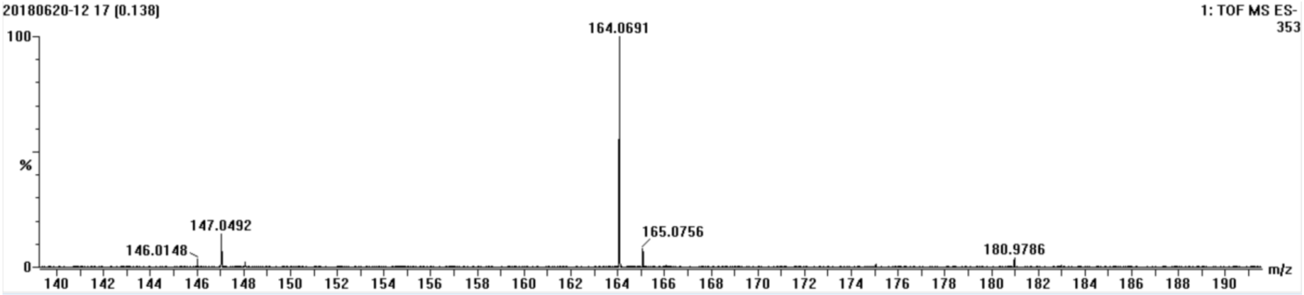

Supplementary Figure 46. HRMS spectrum of (*R*)-**6a**. (ESI$^-$) *m/z*: $C_9H_{11}NO_2$ [M-H]$^-$, 164.0691.

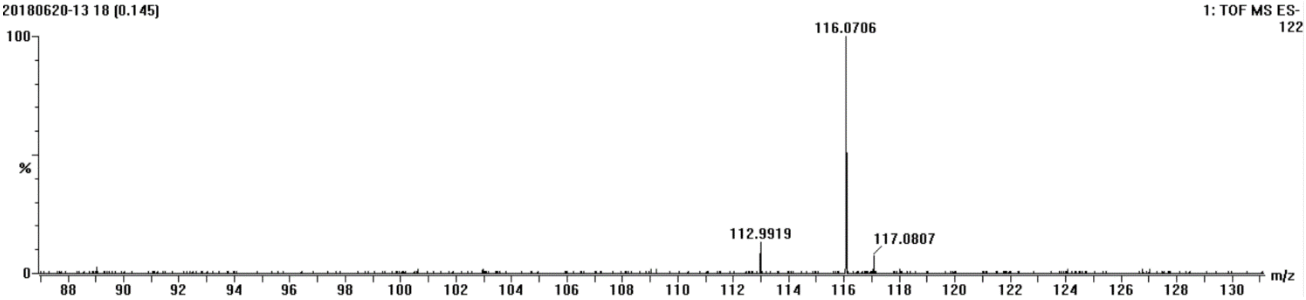

Supplementary Figure 47. HRMS spectrum of (*R*)-**6h**. (ESI$^-$) *m/z*: $C_5H_{11}NO_2$ [M-H]$^-$, 116.0706.

Supplementary Tables

Supplementary Table 1. Plasmids used in this study.

Plasmids	Copy number	Resistance	Origin	Abbreviation
pACYCDuet-1	10	chloramphenicol	P15A	A
pCDFDuet-1	20	streptomycin	CDF	C
pETDuet-1	40	ampicillin	PBR322	E
pRSFDuet-1	100	kanamycin	RSF	R

Supplementary Table 2. Constructed plasmids of each module.

Module	Expressed gene	Recombinant plasmids
Basic module (BM)	*Pa*TA, *Cg*TD^{F114A,R229T}	A-BM, C-BM, E-BM, R-BM
Extender module 1 (EM1)	L-HicDH, *Cb*FDH	A-EM1$_L$, C-EM1$_L$, E-EM1$_L$, R-EM1$_L$
	D-HicDH, *Cb*FDH	A-EM1$_D$, C-EM1$_D$, E-EM1$_D$, R-EM1$_D$
Extender module 2 (EM2)	L-AADH (*Bb*Phe), *Cb*FDH	A-EM2$_L$, C-EM2$_L$, E-EM2$_L$, R-EM2$_L$
	D-AADH (DAPDH), *Bm*GDH	A-EM2$_D$, C-EM2$_D$, E-EM2$_D$, R-EM2$_D$
Extender module 3 (EM3)	*Bs*DAAT, *Ec*GluDH, *Cb*FDH, *Bs*Glr	A-EM3, C-EM3, E-EM3, R-EM3

Supplementary Table 3. Host strains and plasmids enabling the synthesis of α-functionalised organic acids.

Strain[a]	Recombinant plasmids[b] in the strain	Cascade Reactions
E. coli (TA)	pETDuet-*Pa*TA	**1a-i** to **3a-i**
E. coli (TD)	pETDuet-*Cg*TD$_{WT}$	**3a-i** to **4a-i**
E. coli (OA01)	pACYC-*Pa*TA-*Cg*TD (A-BM)	**1a-i** to **4a-i**
E. coli (OA02)	pCDF-*Pa*TA-*Cg*TD (C-BM)	**1a-i** to **4a-i**
E. coli (OA03)	pET-*Pa*TA-*Cg*TD (E-BM)	**1a-i** to **4a-i**
E. coli (OA04)	pRSF-*Pa*TA-*Cg*TD (R-BM)	**1a-i** to **4a-i**
E. coli (OA05)	pACYC-*Pa*TA-*Cg*TD, pCDF-L-HicDH-*Cb*FDH (A-BM_C-EM1$_L$)	**1a-i** to (*S*)-**5a-i**
E. coli (OA06)	pACYC-*Pa*TA-*Cg*TD, pET-L-HicDH-*Cb*FDH (A-BM_E-EM1$_L$)	**1a-i** to (*S*)-**5a-i**
E. coli (OA07)	pACYC-*Pa*TA-*Cg*TD, pRSF-L-HicDH-*Cb*FDH (A-BM_R-EM1$_L$)	**1a-i** to (*S*)-**5a-i**
E. coli (OA08)	pCDF-*Pa*TA-*Cg*TD, pACYC-L-HicDH-*Cb*FDH (C-BM_A-EM1$_L$)	**1a-i** to (*S*)-**5a-i**
E. coli (OA09)	pCDF-*Pa*TA-*Cg*TD, pET-L-HicDH-*Cb*FDH (C-BM_E-EM1$_L$)	**1a-i** to (*S*)-**5a-i**
E. coli (OA10)	pCDF-*Pa*TA-*Cg*TD, pRSF-L-HicDH-*Cb*FDH (C-BM_R-EM1$_L$)	**1a-i** to (*S*)-**5a-i**
E. coli (OA11)	pET-*Pa*TA-*Cg*TD, pACYC-L-HicDH-*Cb*FDH (E-BM_A-EM1$_L$)	**1a-i** to (*S*)-**5a-i**
E. coli (OA12)	pET-*Pa*TA-*Cg*TD, pCDF-L-HicDH-*Cb*FDH (E-BM_C-EM1$_L$)	**1a-i** to (*S*)-**5a-i**
E. coli (OA13)	pET-*Pa*TA-*Cg*TD, pRSF-L-HicDH-*Cb*FDH (E-BM_R-EM1$_L$)	**1a-i** to (*S*)-**5a-i**
E. coli (OA14)	pRSF-*Pa*TA-*Cg*TD, pACYC-L-HicDH-*Cb*FDH (R-BM_A-EM1$_L$)	**1a-i** to (*S*)-**5a-i**
E. coli (OA15)	pRSF- *Pa*TA-*Cg*TD, pCDF-L-HicDH-*Cb*FDH (R-BM_C-EM1$_L$)	**1a-i** to (*S*)-**5a-i**
E. coli (OA16)	pRSF-*Pa*TA-*Cg*TD, pET-L-HicDH-*Cb*FDH (R-BM_E-EM1$_L$)	**1a-i** to (*S*)-**5a-i**
E. coli (OA17)	pACYC-*Pa*TA-*Cg*TD, pCDF-D-HicDH-*Cb*FDH (A-BM_C-EM1$_D$)	**1a-i** to (*R*)-**5a-i**
E. coli (OA18)	pACYC-*Pa*TA-*Cg*TD, pET-D-HicDH-*Cb*FDH (A-BM_E-EM1$_D$)	**1a-i** to (*R*)-**5a-i**
E. coli (OA19)	pACYC-*Pa*TA-*Cg*TD, pRSF-D-HicDH-*Cb*FDH (A-BM_R-EM1$_D$)	**1a-i** to (*R*)-**5a-i**
E. coli (OA20)	pCDF-*Pa*TA-*Cg*TD, pACYC-D-HicDH-*Cb*FDH (C-BM_A-EM1$_D$)	**1a-i** to (*R*)-**5a-i**
E. coli (OA21)	pCDF-*Pa*TA-*Cg*TD, pET-D-HicDH-*Cb*FDH (C-BM_E-EM1$_D$)	**1a-i** to (*R*)-**5a-i**
E. coli (OA22)	pCDF-*Pa*TA-*Cg*TD, pRSF-D-HicDH-*Cb*FDH (C-BM_R-EM1$_D$)	**1a-i** to (*R*)-**5a-i**
E. coli (OA23)	pET-*Pa*TA-*Cg*TD, pACYC-D-HicDH-*Cb*FDH (E-BM_A-EM1$_D$)	**1a-i** to (*R*)-**5a-i**
E. coli (OA24)	pET-*Pa*TA-*Cg*TD, pCDF-D-HicDH-*Cb*FDH (E-BM_C-EM1$_D$)	**1a-i** to (*R*)-**5a-i**
E. coli (OA25)	pET-*Pa*TA-*Cg*TD, pRSF-D-HicDH-*Cb*FDH (E-BM_R-EM1$_D$)	**1a-i** to (*R*)-**5a-i**
E. coli (OA26)	pRSF-*Pa*TA-*Cg*TD, pACYC-D-HicDH-*Cb*FDH (R-BM_A-EM1$_D$)	**1a-i** to (*R*)-**5a-i**
E. coli (OA27)	pRSF-*Pa*TA-*Cg*TD, pCDF-D-HicDH-*Cb*FDH (R-BM_C-EM1$_D$)	**1a-i** to (*R*)-**5a-i**
E. coli (OA28)	pRSF-*Pa*TA-*Cg*TD, pET-D-HicDH-*Cb*FDH (R-BM_E-EM1$_D$)	**1a-i** to (*R*)-**5a-i**
E. coli (OA29)	pACYC-*Pa*TA-*Cg*TD, pCDF-*Bb*PheDH-*Cb*FDH (A-BM_C-EM2$_L$)	**1a-i** to (*S*)-**6a-i**
E. coli (OA30)	pACYC-*Pa*TA-*Cg*TD, pET-*Bb*PheDH-*Cb*FDH (A-BM_E-EM2$_L$)	**1a-i** to (*S*)-**6a-i**
E. coli (OA31)	pACYC-*Pa*TA-*Cg*TD, pRSF-*Bb*PheDH-*Cb*FDH (A-BM_R-EM2$_L$)	**1a-i** to (*S*)-**6a-i**
E. coli (OA32)	pCDF-*Pa*TA-*Cg*TD, pACYC-*Bb*PheDH-*Cb*FDH (C-BM_A-EM2$_L$)	**1a-i** to (*S*)-**6a-i**

E. coli (OA33)	pCDF-*Pa*TA-*Cg*TD, pET-*Bb*PheDH-*Cb*FDH (C-BM_E-EM2$_L$)	**1a-i** to (*S*)-**6a-i**
E. coli (OA34)	pCDF-*Pa*TA-*Cg*TD, pRSF-*Bb*PheDH-*Cb*FDH (C-BM_R-EM2$_L$)	**1a-i** to (*S*)-**6a-i**
E. coli (OA35)	pET-*Pa*TA-*Cg*TD, pACYC-*Bb*PheDH-*Cb*FDH (E-BM_A-EM2$_L$)	**1a-i** to (*S*)-**6a-i**
E. coli (OA36)	pET-*Pa*TA-*Cg*TD, pCDF-*Bb*PheDH-*Cb*FDH (E-BM_C-EM2$_L$)	**1a-i** to (*S*)-**6a-i**
E. coli (OA37)	pET-*Pa*TA-*Cg*TD, pRSF-*Bb*PheDH-*Cb*FDH (E-BM_R-EM2$_L$)	**1a-i** to (*S*)-**6a-i**
E. coli (OA38)	pRSF-*Pa*TA-*Cg*TD, pACYC-*Bb*PheDH-*Cb*FDH (R-BM_A-EM2$_L$)	**1a-i** to (*S*)-**6a-i**
E. coli (OA39)	pRSF-*Pa*TA-*Cg*TD, pCDF-*Bb*PheDH-*Cb*FDH (R-BM_C-EM2$_L$)	**1a-i** to (*S*)-**6a-i**
E. coli (OA40)	pRSF-*Pa*TA-*Cg*TD, pET-*Bb*PheDH-*Cb*FDH (R-BM_E-EM2$_L$)	**1a-i** to (*S*)-**6a-i**
E. coli (OA41)	pACYC-*Pa*TA-*Cg*TD, pCDF-DAPDH-*Bm*GDH (A-BM_C-EM2$_D$)	**1a-i** to (*R*)-**6a-i**
E. coli (OA42)	pACYC-*Pa*TA-*Cg*TD, pET-DAPDH-*Bm*GDH (A-BM_E-EM2$_D$)	**1a-i** to (*R*)-**6a-i**
E. coli (OA43)	pACYC-*Pa*TA-*Cg*TD, pRSF-DAPDH-*Bm*GDH (A-BM_R-EM2$_D$)	**1a-i** to (*R*)-**6a-i**
E. coli (OA44)	pCDF-*Pa*TA-*Cg*TD, pACYC-DAPDH-*Bm*GDH (C-BM_A-EM2$_D$)	**1a-i** to (*R*)-**6a-i**
E. coli (OA45)	pCDF-*Pa*TA-*Cg*TD, pET-DAPDH-*Bm*GDH (C-BM_E-EM2$_D$)	**1a-i** to (*R*)-**6a-i**
E. coli (OA46)	pCDF-*Pa*TA-*Cg*TD, pRSF-DAPDH-*Bm*GDH (C-BM_R-EM2$_D$)	**1a-i** to (*R*)-**6a-i**
E. coli (OA47)	pET-*Pa*TA-*Cg*TD, pACYC-DAPDH-*Bm*GDH (E-BM_A-EM2$_D$)	**1a-i** to (*R*)-**6a-i**
E. coli (OA48)	pET-*Pa*TA-*Cg*TD, pCDF-DAPDH-*Bm*GDH (E-BM_C-EM2$_D$)	**1a-i** to (*R*)-**6a-i**
E. coli (OA49)	pET-*Pa*TA-*Cg*TD, pRSF-DAPDH-*Bm*GDH (E-BM_R-EM2$_D$)	**1a-i** to (*R*)-**6a-i**
E. coli (OA50)	pRSF-*Pa*TA-*Cg*TD, pACYC-DAPDH-*Bm*GDH (R-BM_A-EM2$_D$)	**1a-i** to (*R*)-**6a-i**
E. coli (OA51)	pRSF-*Pa*TA-*Cg*TD, pCDF-DAPDH-*Bm*GDH (R-BM_C-EM2$_D$)	**1a-i** to (*R*)-**6a-i**
E. coli (OA52)	pRSF-*Pa*TA-*Cg*TD, pET-DAPDH-*Bm*GDH (R-BM_E-EM2$_D$)	**1a-i** to (*R*)-**6a-i**
E. coli (OA53)	pACYC-*Pa*TA-*Cg*TD, pCDF-*Bs*AATA-*Bs*GluDH-*Ec*Glr-*Cb*FDH (A-BM_C-EM3)	**1a-i** to (*R*)-**6a-i**
E. coli (OA54)	pACYC-*Pa*TA-*Cg*TD, pET-*Bs*AATA-*Bs*GluDH-*Ec*Glr-*Cb*FDH (A-BM_E-EM3)	**1a-i** to (*R*)-**6a-i**
E. coli (OA55)	pACYC-*Pa*TA-*Cg*TD, pRSF-*Bs*AATA-*Bs*GluDH-*Ec*Glr-*Cb*FDH (A-BM_R-EM3)	**1a-i** to (*R*)-**6a-i**
E. coli (OA56)	pCDF-*Pa*TA-*Cg*TD, pACYC-*Bs*AATA-*Bs*GluDH-*Ec*Glr-*Cb*FDH (C-BM_A-EM3)	**1a-i** to (*R*)-**6a-i**
E. coli (OA57)	pCDF-*Pa*TA-*Cg*TD, pET-*Bs*AATA-*Bs*GluDH-*Ec*Glr-*Cb*FDH (C-BM_E-EM3)	**1a-i** to (*R*)-**6a-i**
E. coli (OA58)	pCDF-*Pa*TA-*Cg*TD, pRSF-*Bs*AATA-*Bs*GluDH-*Ec*Glr-*Cb*FDH (C-BM_R-EM3)	**1a-i** to (*R*)-**6a-i**
E. coli (OA59)	pET-*Pa*TA-*Cg*TD, pACYC-*Bs*AATA-*Bs*GluDH-*Ec*Glr-*Cb*FDH (E-BM_A-EM3)	**1a-i** to (*R*)-**6a-i**
E. coli (OA60)	pET-*Pa*TA-*Cg*TD, pCDF-*Bs*AATA-*Bs*GluDH-*Ec*Glr-*Cb*FDH (E-BM_C-EM3)	**1a-i** to (*R*)-**6a-i**
E. coli (OA61)	pET-*Pa*TA-*Cg*TD, pRSF-*Bs*AATA-*Bs*GluDH-*Ec*Glr-*Cb*FDH (E-BM_R-EM3)	**1a-i** to (*R*)-**6a-i**

E. coli (OA62)	pRSF-*Pa*TA-*Cg*TD, pACYC-*Bs*AATA-*Bs*GluDH-*Ec*Glr-*Cb*FDH (R-BM_A-EM3)	**1a-i** to (*R*)-**6a-i**
E. coli (OA63)	pRSF-*Pa*TA-*Cg*TD, pCDF-*Bs*AATA-*Bs*GluDH-*Ec*Glr-*Cb*FDH (R-BM_C-EM3)	**1a-i** to (*R*)-**6a-i**
E. coli (OA64)	pRSF-*Pa*TA-*Cg*TD, pET-*Bs*AATA-*Bs*GluDH-*Ec*Glr-*Cb*FDH (R-BM_E-EM3)	**1a-i** to (*R*)-**6a-i**

[a] The strains were constructed by transforming the corresponding recombinant plasmids into *E. coli* BL21 (DE3) T7 express strains (New England Biolabs).

[b] The recombinant plasmids were constructed on pACYCDuet-1, pCDFDuet-1, pETDuet-1, and pRSFDuet-1 (Novagen), respectively.

Supplementary Table 4. Isolation of several α-functionalized organic acids and their applications.

Compound	Structure[a]	Applications
**4a**	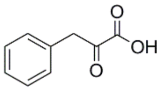	Precursor of phenyllactic acid and phenylalanine
**4i**	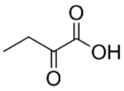	Precursor of 2-hydroxybutyrate and 2-amino butyrate
(*S*)-**5e**	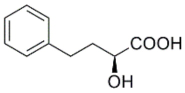	Key precursor for the synthesis of angiotensin converting enzyme inhibitor (ACEI).
(*S*)-**5i**	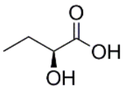	Key precursor for the synthesis of the peroxisome proliferator-activated receptor a (PPARa) agonist (*R*)-K-13675[2].
(*R*)-**5a**	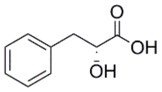	Chiral precursor of Englitazone, Statine, Danshensu, anti-HIV reagents[3].
(*R*)-**5i**	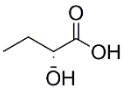	Key precursor for the synthesis of PPAR agonist MK-0533[4].
(*S*)-**6e**	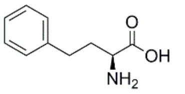	Key precursor in producing angiotensin-converting enzyme inhibitors[5].
(*S*)-**6i**	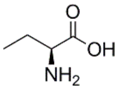	Key precursor for the synthesis of anti-tuberculotic ethambutol, brivaracetam, and the anti-epileptic levetiracetam[6].
(*R*)-**6a**	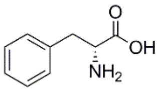	of the antidiabetic drug nateglinide [7], and the antitumor drug ubenimex[8].
(*R*)-**6h**	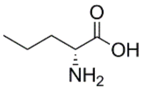	Key precursor of Pamamycin-607[9] and Epilachnene[10].

[a] The structures were identified by NMR (Supplementary Figure 28-S37) and HRMS (Supplementary Figure 38-S47) analysis.

Supplementary Table 5. Primers used in this study for plasmid constructions.

Name	Sequence (5' to 3')
**Enzyme Expression**	
BglII-*Pa*TA-F	GAAGATCTCATGCCTGTCATCGACCTG
KpnI-*Pa*TA-R	GGGGTACCTTATGAGCGACGAAAGG
BamHI-*Cg*TD-F	CGGGATCCGATGAGTGAAACATACGTGTCTG
SacI-*Cg*TD-R	CGAGCTCTTAGGTCAAGTATTCGTACTCAG
BamHI-D-AADH-F	CGGGATCCGATGGATAAACTGCGTGTTGC
SacI-D-AADH-R	CGAGCTCTTACACCAGTTTGCGAATCCATG
EcoRI-L-HicDH-F	CGGAATTCGATGGCACGTAAGATTGGAATTATCG
SacI-L-HicDH-R	CGAGCTCTTAGAGTGTATCCACAATTTCGTCG
EcoRI-D-HicDH-F	CCGGAATTCGATGAAGATCATTGCTTATGGAG
SacI-D-HicDH-R	CGAGCTCCTATTTGGCTGGACCGGTCACCTC
BglII-*Cb*FDH-F	GAAGATCTCATGAAGATCGTTTTAGTCTTATATG
KpnI-*Cb*FDH-R	GGGGTACCTTATTTCTTATCGTGTTTAC
BglII-*Bm*GDH-F	GAAGATCTCATGTATAAAGATTTAGAAG
KpnI-*Bm*GDH-R	GGGGTACCTTATCCGCGTCCTGCTTG
KpnI-EcoRI-*Cb*FDH-R	CGGGTACCGAATTCTTATTTCTTATCGTGTTTAC
EcoRI-RBS-*Bs*GLR-F	CGGAATTCTAAGGAGATATATAATGGAACAACCAATAGGAGTCATTG
KpnI-BsGLR-R	GGGGTACCCTATCTTTTAATCGGTTCTTGCAG
BamHI-*Bb*PheDH-F	CGGGATCCGAGCTTAGTAGAAAAAACATC
SacI-*Bb*PheDH-R	CGAGCTCTTAGTTGCGAATATCCCATTTTG
SacI-RBS-*Ec*GluDH-F	CGAGCTCTAAGGAGATATATAATGGATCAGACATATTCTCTGGAG-3'
SalI-*Ec*GluDH-R	ACGCGTCGACTTAAATCACACCCTGCGCCAGCATC
BamHI-*Bs*AATA-F	CGGGATCCGAAGGTTTTAGTCAATGGC
SacI-*Bs*AATA-R	CGAGCTCTTATGAAATGCTAGCAGCCTGTTG
**Enzyme Mutagenesis**	
F114N-F	CTGGCAATAACNBTGACGAAGCATCGGCTG
F114N-R	CAGCCGATGCTTCGTCAVNGTTATTGCCAG
R229N-F	GCAGCAGTCAAANBTGTCGGAGATCTC
R229N-R	GAGATCTCCGACAVNTTTGACTGCTGC

Supplementary Methods

General

Commercial reagents, standards, and solvents were purchased from Sigma Aldrich, Meryer Chemicals, Aladdin, and TCI chemicals, and used without further purification.

Genetic constructions

All genetic constructions were carried out by using standard molecular biology techniques with LATaq and rTaq DNA polymerase, restriction enzymes and T4 DNA ligase (all from Takara, Japan). Cloning inserts were created via PCR of ORFs of interest from their respective genomic or codon-optimized DNA with Phusion polymerase. Heterologous genes were amplified from their respective genomic (Supplementary Table 5) except for L-HicDH, D-HicDH, and DAPDH, which were synthesized by GenScript (Piscataway, NJ) with codon optimization (see Supplementary Methods for optimized sequences). Genes of *Pa*TA (NCBI: AE004953), *Cg*TD (GenBank: SJM46940.1), *Cb*FDH (GenBank: CAA09466.2), and L-AADH (NCBI: WP_063384663.1) were amplified from the genome of *Staphylococcus aureus*, *Corynebacterium glutamicum*, *Candida boidinii*, and *Bacillus badius*, respectively. *Ec*AATA (NCBI: WP_054495849.1) and *Ec*GluDH (NCBI: WP_044790188.1) genes were amplified from *E. coli*. *Bs*AATA (GenBank: AFQ56883.1) and *Bs*Glr (GenBank: BAA28871.1) genes were amplified from *B. subtilis*.

Optimized sequences

The diaminopimelate dehydrogenase gene (from *Symbiobacterium thermophilum* IAM 14863) was synthesized and codon optimized for *E. coli*. The optimized DNA sequence and is as follow:

DNA sequence: GGATCCGATGGATAAACTGCGTGTTGCCGTGGTTGGTTATGGTAACGTTGG
CCGCTATGCCTTAGAGGCCGTTCAAGCTGCCCCGGATATGGAACTGGTTGGTGTGGTTCG
TCGTAAAGTGCTGGCCGCAACCCCGCCCGAACTGACTGGTGTGCGCGTTGTTACCGATAT
TAGCCAGCTGGAAGGCGTTCAAGGTGCTTTACTGTGTGTTCCGACCCGCAGCGTGCCGG
AATATGCAGAGGCCATGCTGCGCCGCGGCATTCATACAGTGGATAGCTACGATATTCACGG
TGATCTGGCCGATCTGCGTCGTCGTTTAGATCCCGTTGCCCGCGAACATGGTGCCGCAGC
AGTGATTAGCGCCGGCTGGGACCCCGGTACCGACAGCATTATCCGCGCACTGCTGGAATT
TATGGCCCCGAAAGGCATCACCTATACCAACTTTGGCCCGGGTATGAGCATGGGTCACAG
CGTGGCCGTGAAAGCAATCCCGGGCGTGCGTGATGCTTTAAGCATGACCATTCCGGCCGG
CATGGGCGTTCACAAACGTGCCGTGTATGTGGAACTGGAACCGGGTGCCGATTTCGCCG

AAGTGGAACGCGCAATTAAGACCGATCCGTACTTTGTGCGCGACGAAACCCGCGTGACC
CAAGTTGAAAGCGTTAGCGCTTTAATGGATGTGGGCGTGGGTGTGGTGATGGAACGCAA
AGGCGTTAGCGGTGCCACCCATAACCAGCTGTTTCGCTTCGAGATGCGCATCAACAATCC
GGCACTGACCGCCCAAGTTATGGTTGCAGCTTTACGCGCAGCAGCACGTCAGAAACCGG
GCTGCTATACCATGATCGAGATCCCGGTGATCGACTACTTACCGGGTGATCGCGAAGCATG
GATTCGCAAACTGGTGTAAGAGCTC

Protein sequence: MDKLRVAVVGYGNVGRYALEAVQAAPDMELVGVVRRKVLAATPPELTG
VRVVTDISQLEGVQGALLCVPTRSVPEYAEAMLRRGIHTVDSYDIHGDLADLRRRLDPVAR
EHGAAAVISAGWDPGTDSIIRALLEFMAPKGITYTNFGPGMSMGHSVAVKAIPGVRDALSM
TIPAGMGVHKRAVYVELEPGADFAEVERAIKTDPYFVRDETRVTQVESVSALMDVGVGVV
MERKGVSGATHNQLFRFEMRINNPALTAQVMVAALRAAARQKPGCYTMIEIPVIDYLPGDR
EAWIRKLV

The L-2-hydroxyisocaproate dehydrogenase (L-HicDH) gene (from *Lactobacillus confuses*) was synthesized and codon optimized for *E. coli*. The optimized DNA sequence and is as follow:

DNA sequence: ATGGCACGTAAGATTGGAATTATCGGCCTTGGAAACGTTGGGGCTGCAGTA
GCGCACGGATTGATTGCACAAGGTGTAGCCGACGACTACGTCTTTATTGATGCAAACGAA
GCAAAGGTGAAGGCTGATCAAATTGATTTCCAAGACGCAATGGCGAACTTGGAAGCGCA
CGGTAACATTGTGATTAACGATTGGGCAGCCTTGGCTGATGCTGATGTTGTGATTTCAACA
CTGGGGAACATCAAGTTGCAACAAGACAACCCAACCGGTGACCGTTTTGCTGAGTTGAA
GTTTACCAGCAGCATGGTGCAATCAGTCGGCACAAACTTGAAGGAATCTGGTTTCCACGG
CGTATTGGTCGTGATTTCAAACCCGGTCGACGTGATTACGGCCTTGTTCCAACACGTGAC
TGGTTTCCCAGCTCACAAGGTTATCGGAACCGGTACTTTGCTTGACACGGCGCGTATGCA
ACGTGCAGTTGGTGAGGCGTTTGATTTGGATCCACGTTCTGTTTCAGGTTACAACTTGGG
TGAGCACGGTAACTCACAATTCGTAGCTTGGTCAACGGTGCGCGTGATGGGTCAACCAAT
CGTGACGTTGGCTGATGCCGGCGATATTGACTTGGCGGCCATCGAAGAGGAAGCACGTA
AGGGTGGCTTCACGGTCTTGAATGGTAAGGGCTACACGAGTTATGGTGTTGCAACGTCAG
CAATCCGCATTGCCAAGGCTGTTATGGCTGACGCGCATGCTGAATTGGTTGTCTCAAATCG
TCGCGATGACATGGGAATGTACTTGTCATACCCAGCGATTATTGGTCGCGATGGTGTCTTG
GCAGAAACGACGCTTGATTTGACGACGGATGAGCAAGAAAAGCTTTTGCAATCACGTGA
CTACATCCAACAACGTTTCGACGAAATTGTGGATACACTCTAA

Protein sequence: MARKIGIIGLGNVGAAVAHGLIAQGVADDYVFIDANEAKVKADQIDFQDA

MANLEAHGNIVINDWAALADADVVISTLGNIKLQQDNPTGDRFAELKFTSSMVQSVGTNLK
ESGFHGVLVVISNPVDVITALFQHVTGFPAHKVIGTGTLLDTARMQRAVGEAFDLDPRSVSG
YNLGEHGNSQFVAWSTVRVMGQPIVTLADAGDIDLAAIEEEARKGGFTVLNGKGYTSYGVA
TSAIRIAKAVMADAHAELVVSNRRDDMGMYLSYPAIIGRDGVLAETTLDLTTDEQEKLLQSR
DYIQQRFDEIVDTL

The D-2-hydroxyisocaproate dehydrogenase (D-HicDH) gene (from *Lactobacillus paracasei*) was synthesized and codon optimized for *E. coli*. The optimized DNA sequence and is as follow:

DNA sequence: ATGAAGATCATTGCTTATGGAGCCCGCGTGGACGAGATCCAGTATTTCAA
ACAGTGGGCCAAGGACACTGGAAACACTCTTGAATACCACACTGAGTTCCTGGATGAGA
ATACTGTTGAGTGGGCCAAGGGTTTTGATGGCATCAATTCCCTGCAAACGACGCCGTAC
GCTGCGGGAGTGTTTGAAAAAATGCATGCGTACGGTATTAAATTTTTGACCATTCGCAA
CGTGGGTACTGACAATATCGACATGACTGCTATGAAGCAGTATGGGATCCGCCTGTCAA
ACGTACCGGCGTACTCTCCAGCTGCCATCGCCGAGTTCGCCTTGACAGACACTTTGTACT
TATTACGTAATATGGGGAAAGTTCAAGCCCAATTGCAAGCTGGGGACTACGAAAAGGC
CGGGACCTTTATTGGCAAAGAATTAGGACAGCAGACAGTTGGAGTGATGGGTACCGGA
CATATCGGCCAAGTAGCCATCAAACTGTTTAAGGGGTTTGGGGCCAAGGTTATTGCATA
CGATCCATACCCCATGAAAGGAGATCATCCCGATTTTGACTACGTCTCTCTTGAAGATCT
TTTTAAGCAGAGCGACGTTATCGACCTGCATGTACCAGGGATTGAACAGAATACGCATA
TCATCAATGAGGCCGCGTTCAACCTTATGAAGCCCGGCGCAATCGTTATTAACACAGCC
CGCCCTAATCTGATCGACACGCAAGCCATGCTGAGCAACCTGAAAAGCGGGAAACTGG
CTGGCGTGGGCATCGACACGTACGAGTACGAAACTGAAGACCTTTTGAATCTGGCAAAA
CATGGATCCTTCAAAGATCCATTATGGGATGAGTTGTTAGGCATGCCTAACGTTGTATTG
TCACCGCATATCGCGTACTACACGGAGACGGCTGTGCATAATATGGTTTATTTTTCTTTA
CAACATCTTGTCGATTTCCTGACCAAGGGAGAAACGAGCACCGAGGTGACCGGTCCAGC
CAAATAG

Protein sequence: MKIIAYGARVDEIQYFKQWAKDTGNTLEYHTEFLDENTVEWAKGFDGINSL
QTTPYAAGVFEKMHAYGIKFLTIRNVGTDNIDMTAMKQYGIRLSNVPAYSPAAIAEFALTDTL
YLLRNMGKVQAQLQAGDYEKAGTFIGKELGQQTVGVMGTGHIGQVAIKLFKGFGAKVIAY
DPYPMKGDHPDFDYVSLEDLFKQSDVIDLHVPGIEQNTHIINEAAFNLMKPGAIVINTARPNL
IDTQAMLSNLKSGKLAGVGIDTYEYETEDLLNLAKHGSFKDPLWDELLGMPNVVLSPHIAY
YTETAVHNMVYFSLQHLVDFLTKFKPARKLLVQQVVN

Fermentation medium and conditions

A single colony of recombinant *E. coli* strain was cultivated overnight (10-12 h, 37 ºC) in LB medium (10 g l^{-1} peptone, 5 g l^{-1} yeast extract, and 10 g l^{-1} NaCl; pH 7.0) with appropriate antibiotics (50 μg ml^{-1} chloramphenicol, 34 μg ml^{-1} streptomycin, 100 μg ml^{-1} ampicillin, 50 μg ml^{-1} kanamycin or a combination of them) and used as the inoculum (1%). The culture was then transferred into 50 ml Terrific Broth (TB) medium (24 g l^{-1} yeast extract, 12 g l^{-1} tryptone, 5 g l^{-1} glucose, 2.31 g l^{-1} KH_2PO_4, and 16.43 g l^{-1} K_2HPO_4; pH 7.0) containing appropriate antibiotics in a 500 ml flask. When the OD_{600} of the culture broth reached 0.6-0.8, isopropyl β-D-1-thiogalactopyranoside (IPTG) was added to a final concentration of 0.4 mM to induce gene expression. The cells were inducted at 25 °C for 16 h and collected by centrifugation (12000×g, 5 min). Then, the cell pellets were resuspended in an appropriate buffer to the desired density as resting cells for biotransformation.

Additional larger fermentations were conducted in a 7.5 L fermentation system (INFORS HT Labfors, Switzerland) with an air flow rate of 1.5 vvm and a stirrer speed of 600 rpm. The pH was maintained at 7.0 by automatically feeding concentrated carbon and nitrogen resources (400 g l^{-1} glucose, 100 g l^{-1} yeast extract, and 25 g l^{-1} tryptone; start feeding after a steep rise in dissolved oxygen, 14 ml h^{-1}). Enzyme expression was induced at 25 ºC with 5 g l^{-1} lactose at an optical density of 4 at 600 nm. Pre-cultures were grown in 500 ml flasks as described above. The cell pellets were collected for preparative biotransformation after 12 h induction.

Enzyme purification

*Pa*TA and *Cg*TD were individually overexpressed and purified from *E. coli* BL21 (DE3) with pETDuet-1 plasmid. Strains were first grown in LB medium with suitable antibiotics at 37ºC, 200 rpm. When the OD_{600} of the culture broth reached 0.6-0.8, IPTG was added to a final concentration of 0.4 mM to induce gene expression. The cells were inducted at 25 °C for 16 h and collected by centrifugation (12000×g, 5 min), and resuspended in buffer A (500 mM NaCl, 20 mM imidazole, 20 mM phosphate buffer, pH 7.4; 10 ml g^{-1} of wet weight). The cell suspensions were lysed by sonication and centrifuged at 14,000 g for 0.5 h. The subsequent experiments were performed on an ÄKTA pure system (GE Healthcare, Waukesha, WI) with a HisTrap HP column (5 ml, GE Healthcare). All operations were conducted at 4ºC when necessary.

Directed evolution experiments

In order to broaden the substrate channel, small amino acids were selected (Ala, Gly, Cys, Ile, Leu,

Ser, Thr, and Val) to modify the substrate channel (Phe114 and Arg229) with different levels of hindrance. Therefore, the NBT degeneracy was chosen because 8 of the 12 amino acids encoded by NBT were small amino acids. A smart library was constructed by a double-site semisaturated mutation strategy. In order to cover the designed 95% of all possible sequence variants, more than 430 colonies should be screened. Therefore, we screened 500 colonies in this experiment.

Isolation protocols

Isolation protocol for **4** and **5**: The reaction supernatant was adjusted to pH>12 with NaOH (8 M) and washed with ethyl acetate two times (2×25 ml) to remove remained aldehyde and other organic impurities. The aqueous phase was acidified to pH 1 with 8.0 M HCl and extracted with ethyl acetate (3×100 ml). The organic phases were combined, dried over Na_2SO_4, filtered through a pad of silica gel and evaporated under vacuum to afford corresponding products in high chemical purity.

Isolation protocol for **6**: The amino acids were purified using a Dowex 50WX8 cation exchange column. First, the resin was conditioned by washing with NH_4OH (2 M, 2×30 ml), HCl (2 M, 2×30 ml) and H_2O (4×30 ml). Then, the crude reaction mixture was acidified with 1 M HCl and loaded onto the column. Finally, the column was washed with HCl (1M, 2×30 ml), H_2O (4×30 ml) and eluted with NH_4OH (2 M, 4×30 ml). Fractions containing amino acids were combined and lyophilized to remove the water, and then purified by preparation thin liquid chromatography (PTLC) (^{n}BuOH/H_2O/AcOH, 4:1:1). Silica gel containing target amino acid was collected and eluted with ^{n}BuOH/H_2O (2:1). After filtration, the organic solvent was removed by evaporation, and the product was dried overnight under vacuum. The so obtained solid was washed with (EtOH/H_2O, 9:1) affording the corresponding amino acids **6** in high chemical purity.

The purified products were further identified by NMR and HRMS analysis.

Chromatography analysis

Identification of products are accomplished by HPLC analysis based on the integration of monomer peaks using external commercial standards.

Analysis of the concentration of **2**, **3**, and **6** were carried out using an Agilent 1260 HPLC system and a Agilent SB-AQ C18 column (250×4.6 mm, 5 μm) with gradient elution at 35 °C and a flow rate of 1 ml min^{-1}. The mobile phase gradient was formed by buffer A (10 mM KH_2PO_4 buffer, pH 5.3 adjusted by KOH) and buffer B (buffer A/acetonitrile/methanol, 1:5:3 by volume, pH 5.3 adjusted by acetic

acid). The A/B ratios were 80:20, 73:27, 50:50, 30:70, 25:75, 20:80, 40:60, 60:40, and 20:80, at run times of 0, 4, 8, 12, 16, 20, 24, 28 and 32 min, respectively. *o*-Phthaldialdehyde (OPA) was used as the pre-column derivation reagent, and the products were monitored by a fluorescence detector (FLD) with excitation and emission wavelength at 330 and 465 nm, respectively.

Analysis of the concentration of **1a-g**, **4a-g**, and **5a-g** was carried out using an Agilent 1260 HPLC system with an UV detector at 254 nm. Column: Phenomenex Luna C18 column (250×4.6mm, 5 μm), flow: 1 ml min^{-1}, temperature: 30 ºC, mobile phase: 60% methanol with 0.05 % TFA.

Analysis of the concentration of **1h-i**, **4h-i**, and **5h-i** was performed on Dionex UltiMate 3000 HPLC system (Thermo Scientific, Waltham, MA, USA) with an Aminex HPX-87H column (300×7.8 mm, 9 μm; Bio-Rad Laboratories, Inc., Hercules, CA, USA) and monitored with a refractive index detector (RID). Mobile phase: 5 mM H_2SO_4, flow rate: 0.4 ml min^{-1}, temperature: 55 ºC.

Analysis of the e.e. of **5a-g** was measured using Waters Alliance e2695 HPLC (Waters Co., USA) with UV detector at 210 nm. Column: Daicel Chiralpak IC-3 (250 mm×4.6 mm, 3 μm; Daicel Co., Japan), mobile phase: *n*-hexane/isopropanol/TFA (95/5/0.05), flow rate: 0.8 ml min^{-1}, temperature: 30 ºC.

Analysis of the e.e. of **5h-i** was performed on Dionex UltiMate 3000 HPLC system with a RID detector. Column: MCI GEL CRS10W (300×7.8 mm, Japan), mobile phase: 2 mM $CuSO_4$, flow rate: 0.4 ml min^{-1}, temperature: 25 ºC.

Analysis of the e.e. of **6** was conducted using Agilent 1260 HPLC with Daicel Crownpak CR-I(+) column (150×3 mm, 5 μm; Daicel Co., Japan) and pH 1.5 $HClO_4$ a.q./acetonitrile (80/20, v/v) as the mobile phase. Flow: 0.4 ml min^{-1}, temperature: 25 ºC, wavelength: 210 nm.

Supplementary Notes

2-oxo-3-phenylpropanoic acid: 4a

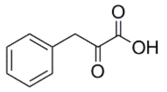

White solid (75% yield, 94% purity). ^{1}H NMR (400 MHz, D_2O+NaOD) δ 7.60 – 7.07 (m, 5H), 3.09 – 2.75 (s, 2H). ^{13}C NMR (101 MHz, D_2O+NaOD) δ 182.45, 138.34, 129.45, 128.60, 126.64, 57.48, 40.79 (Supplementary Figure 28). HRMS (ESI$^-$) *m/z*: calcd. for $C_9H_8O_3$ [M-H]$^-$: 163.0401, found: 163.0287 (Supplementary Figure 38). Literature[11]: ^{1}H NMR (DMSO) δ 2.97 (s, 2H), 7.16~7.67(m, 5H). ^{13}C NMR(DMSO), δ 45.76, 135.25, 128.52, 128.59, 130.29.

2-oxobutanoic acid: **4i**

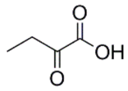

Colorless oil (66% yield, 96% purity). ^{1}H NMR (400 MHz, D_2O+NaOD) δ 3.20 – 1.68 (m, 2H), 1.13 – 0.58 (m, 3H). ^{13}C NMR (101 MHz, D_2O+NaOD) δ 180.93, 101.58, 38.13 (d, *J* = 60.8 Hz), 12.89 (d, *J* = 72.9 Hz) (Supplementary Figure 29). HRMS (ESI$^-$) *m/z*: calcd. for $C_4H_6O_3$ [M-H]$^-$: 101.0244, found: 101.0205 (Supplementary Figure 39). Literature[12]: ^{1}H NMR (250 MHz, $CDCl_3$) δ 5.34 (s, 1H), 2.89-2.99 (m, 2H), 1.40 and 0.88 (tt, J = 129.1, 7.3 Hz, 3H); ^{13}C NMR (400 MHz, $CDCl_3$) δ 4.9, 29.1 (d, J = 35.8 Hz), 157.6, 194.3.

(*S*)-2-hydroxy-4-phenylbutanoic acid: (*S*)-5e

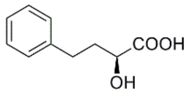

White solid (45% yield, 95% purity). ^{1}H NMR (400 MHz, D_2O+NaOD) δ 7.38 (t, *J* = 7.5 Hz, 2H), 7.35 – 7.23 (m, 3H), 3.27 (t, *J* = 6.4 Hz, 1H), 2.66 (t, *J* = 8.3 Hz, 2H), 1.89 (dh, *J* = 29.0, 7.0 Hz, 2H). ^{13}C NMR (101 MHz, D_2O+NaOD) δ 183.34, 142.46, 128.71, 128.56, 126.08, 65.85, 36.92, 31.53 (Supplementary Figure 30). HRMS (ESI$^-$) *m/z*: calcd. for $C_{10}H_{12}O_3$ [M-H]$^-$: 179.0714, found: 179.0511 (Supplementary Figure 40). Literature[13]: ^{1}H NMR ($CDCl_3$, 400 MHz): δ (ppm) 1.97-2.00 (m, 1H), 2.12-2.18 (m, 1H), 2.76-2.80 (t, *J* = 7.8 Hz, 2H), 4.24 (dd, *J* = 7.6, 5.2 Hz, 1H), 7.16-7.29 (m, 5 H); ^{13}C NMR ($CDCl_3$, 101 MHz): δ (ppm) 30.9, 35.6, 69.4, 126.1, 128.4, 128.5, 140.7, 179.5.

(*S*)-2-hydroxybutanoic acid: (*S*)-5i

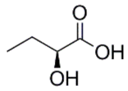

Colorless oil (78% yield, 98% purity). ^{1}H NMR (400 MHz, D_2O+NaOD) δ 3.35 – 3.26 (m, 1H), 1.77 – 1.57 (m, 2H), 0.90 (td, *J* = 7.5, 1.5 Hz, 3H). ^{13}C NMR (101 MHz, D_2O+NaOD) δ 181.81, 56.93, 26.85, 9.12 (Supplementary Figure 31). HRMS (ESI$^-$) *m/z*: calcd. for $C_4H_8O_3$ [M-H]$^-$: 103.0401, found: 103.0333 (Supplementary Figure 41). Literature[14]: ^{1}H NMR ($CDCl_3$) δ 1.00 (t, *J* = 7.4 Hz, 3H) ,1.75-1.88 (m, 2H), 4.24 (dd, *J* = 4.5, 6.9 Hz, 1H), 6.72 (br, 1H)

(*R*)-2-hydroxy-3-phenylpropanoic acid: (*R*)-5a

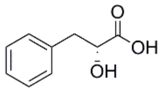

White solid (69% yield, 95% purity). ^1H NMR (400 MHz, D_2O+NaOD) δ 7.43 – 7.27 (m, 5H), 4.27 (ddt, J = 8.0, 4.5, 1.2 Hz, 1H), 3.11 (dd, J = 14.3, 4.4 Hz, 1H), 2.94 – 2.83 (m, 1H). ^{13}C NMR (101 MHz, D_2O+NaOD) δ 180.84, 138.32, 129.44, 128.52, 126.56, 73.44 (d, J = 9.8 Hz), 40.46 (Supplementary Figure 32). HRMS (ESI^-) *m/z*: calcd. for $C_9H_{10}O_3$ $[M-H]^-$: 165.0557, found: 165.0378 (Supplementary Figure 42). Literature[15]: ^1H NMR (400 MHz, D_2O), δ/ppm: 2.76 (dd, J = 14.0, 8.0 Hz, 1H), 2.99 (dd, J = 14.0, 4.2 Hz, 1H), 4.15 (dd, J = 7.9, 4.3 Hz, 1H), 7.35–7.12 (m, 5H).

(*R*)-2-hydroxybutanoic acid: (*R*)-5i

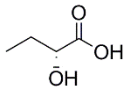

Colorless oil (63% yield, 97% purity). ^1H NMR (400 MHz, D_2O+NaOD) δ 3.35 – 3.26 (m, 1H), 1.77 – 1.57 (m, 2H), 0.90 (td, J = 7.5, 1.5 Hz, 3H). ^{13}C NMR (101 MHz, D_2O+NaOD) δ 181.81, 56.93, 26.85, 9.12 (Supplementary Figure 33). HRMS (ESI^-) *m/z*: calcd. for $C_4H_8O_3$ $[M-H]^-$: 103.0401, found: 103.0520 (Supplementary Figure 43). Literature[14]: ^1H NMR ($CDCl_3$) δ 1.00 (t, J = 7.4 Hz, 3H) ,1.75-1.88 (m, 2H), 4.24 (dd, J = 4.5, 6.9 Hz, 1H), 6.72 (br, 1H).

(*S*)-2-amino-4-phenylbutanoic acid: (*S*)-6e

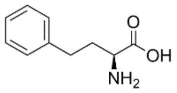

White solid (51% yield, 97% purity). ^1H NMR (400 MHz, D_2O+NaOD) δ7.38 – 7.23 (m, 5H), 3.27 (t, J = 6.4 Hz, 1H), 2.66 (t, J = 8.3 Hz, 2H), 1.89 (dh, J = 29.0, 7.0 Hz, 2H). ^{13}C NMR (101 MHz, D_2O+NaOD) δ 183.34, 142.46, 128.63 (d, J = 15.4 Hz), 126.08, 55.85, 36.92, 31.53 (Supplementary Figure 34). HRMS (ESI^-) *m/z*: calcd. for $C_{10}H_{13}NO_2$ $[M-H]^-$: 178.0874, found: 178.0815 (Supplementary Figure 44). Literature[16]: ^1H NMR (400 MHz, D_2O) δ 2.15–2.36 (m, 2H), 2.70–2.88 (m, 2H), 4.07 (t, J = 5.8 Hz, 1H), 7.27–7.43 (m, 5H); ^{13}C NMR (100 MHz, D_2O) δ 30.5, 31.9, 57.4, 126.6, 128.5, 128.9, 140.3, 173.3.

(*S*)-2-aminobutanoic acid: (*S*)-6i

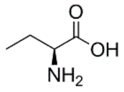

White solid (61% yield, 98% purity). ^1H NMR (400 MHz, D_2O+NaOD) δ 3.18 (t, J = 6.2 Hz, 1H), 1.68 – 1.50 (m, J = 6.8 Hz, 2H), 0.88 (t, J = 7.5 Hz, 3H). ^{13}C NMR (101 MHz, D_2O+NaOD) δ 183.69, 57.21, 27.79, 9.38 (Supplementary Figure 35). HRMS (ESI^-) *m/z*: calcd. for $C_4H_9NO_2$ $[M-H]^-$: 102.0561, found: 102.0523 (Supplementary Figure 45). Literature[17]: ^1H NMR (360 MHz, $C^2H_3O^2H$) δ 1.1 (t, J = 7.5, 3H), 1.9 (m, J = 6.7 Hz, 2H) and 3.5 (t, J = 6.0, 1H).

(*R*)-phenylalanine: (*R*)-6a

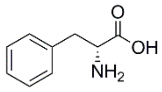

White solid (67% yield, 97% purity). ^{1}H NMR (400 MHz, D_2O+NaOD) δ 7.43 – 7.25 (m, 5H), 3.50 (td, *J* = 6.4, 5.8, 1.6 Hz, 1H), 3.00 (dd, *J* = 13.6, 5.6 Hz, 1H), 2.85 (dd, *J* = 13.5, 7.3 Hz, 1H). ^{13}C NMR (101 MHz, D_2O+NaOD) δ 182.45, 138.34, 129.45, 128.60, 126.64, 57.48, 40.79 (Supplementary Figure 36). HRMS (ESI^-) *m/z*: calcd. for $C_9H_{11}NO_2$ $[M-H]^-$: 164.0717, found: 164.0691 (Supplementary Figure 46). Literature[17]: ^{1}H NMR (360 MHz, $C^2H_3O^2H$) δ 3.0 (dd, *J* = 14.5, 9.0 Hz, 1H), 3.3 (*J* = 4.3 Hz, 1H), 3.8(dd, *J* = 4.3, 9.0 Hz, 1H) and 7.6 (m, 5H).

(*R*)-2-aminopentanoic acid: (*R*)-6h

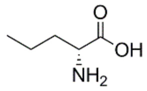

White solid (73% yield, 96% purity). ^{1}H NMR (400 MHz, D_2O+NaOD) δ 3.30 – 3.22 (m, 1H), 1.67 – 1.47 (m, 2H), 1.32 (h, *J* = 7.5 Hz, 2H), 0.91 (t, *J* = 7.4 Hz, 3H). ^{13}C NMR (101 MHz, D_2O+NaOD) δ 183.54, 55.77, 36.72, 18.40, 13.33 (Supplementary Figure 37). HRMS (ESI^-) *m/z*: calcd. for $C_5H_{11}NO_2$ $[M-H]^-$: 116.0717, found: 116.0706 (Supplementary Figure 47). Literature[18]: ^{1}H NMR(D_2O): δ 0.94 (t, *J* = 8.4 Hz, 3H), 1.42(m, 2H), 1.64~1.71 (m, 2H), 3.67(t, *J* = 7.2 Hz, 1H).

Supplementary References

1. Parmeggiani, F., Weise, N.J., Ahmed, S.T. & Turner, N.J. Synthetic and therapeutic applications of ammonia-lyases and aminomutases. *Chem. Rev.* **118**, 73-118 (2017).
2. Yamazaki, Y., Araki, T., Koura, M. & Shibuya, K. A practical synthesis of the PPARα agonist, (*R*)-K-13675, starting from (*S*)-2-hydroxybutyrolactone. *Tetrahedron* **64**, 8155-8158 (2008).
3. Xu, G., Zhang, L. & Ni, Y. Enzymatic preparation of D-phenyllactic acid at high space-time yield with a novel phenylpyruvate reductase identified from *Lactobacillus* sp. CGMCC 9967. *J. Biotechnol.* **222**, 29-37 (2016).
4. Acton, J.J. et al. Discovery of (2*R*)-2-(3-{3-[(4-Methoxyphenyl)carbonyl]-2-methyl-6-(trifluoromethoxy)-1H-indol-1-yl}phenoxy)butanoic acid (MK-0533): a novel selective peroxisome proliferator-activated receptor gamma modulator for the treatment of type 2 diabetes mellitus wi. *J. Med. Chem.* **56**, 3846-3854 (2013).
5. Hayashi, K. et al. Studies on angiotensin converting enzyme inhibitors. 4. Synthesis and angiotensin converting enzyme inhibitory activities of 3-acyl-1-alkyl-2-oxoimidazolidine-4-carboxylic acid derivatives. *J. Med. Chem.* **32**, 289-297 (1989).
6. Tao, R., Jiang, Y., Zhu, F. & Yang, S. A one-pot system for production of L-2-aminobutyric acid from L-threonine by L-threonine deaminase and a NADH-regeneration system based on L-leucine dehydrogenase and formate dehydrogenase. *Biotechnol. Lett.* **36**, 835-841 (2014).
7. Dunn, C.J. & Faulds, D. Nateglinide. *Drugs* **60**, 607–615 (2000).
8. Jiang, Y. et al. Synthesis and biological characterization of ubenimex-fluorouracil conjugates for anti-cancer therapy. *Eur. J. Med. Chem.* **143**, 334-347 (2017).
9. Fraser, B.H., Mulder, R.J. & Perlmutter, P. The total synthesis of pamamycin-607. Part 2: Synthesis of the C6–C18 domain. *Tetrahedron* **62**, 2857-2867 (2006).
10. Farmer, J.J., Attygalle, A.B., Smedley, S.R., Eisner, T. & Meinwald, J. Defensive mechanisms of arthropods. Part 144. Absolute configuration of insect-produced epilachnene. *Tetrahedron Lett.* **38**, 2787-2790 (1997).
11. Zhou, J.L., Shi, X.W., Hao, H.S., Shi, W. & Chen-Rui, Z.I. Synthesis of α-ketophenylalanine calcium. *Fine Chemical Intermediates* **40**, 31-32 (2010).
12. Lichtenecker, R., Ludwiczek, M.L., Schmid, W. & Konrat, R. Simplification of protein NOESY spectra using bioorganic precursor synthesis and NMR spectral editing. *J. Am. Chem. Soc.* **126**, 5348-5349 (2004).
13. Guo, T. & Chien-Hong, C. Synthesis of α-hydroxy carboxylic acids via a Nickel(II)- catalyzed hydrogen transfer process. *Adv. Synth. Catal.* **353**, 1918-1922 (2011).

14. Chenault, H.K. et al. Enzymatic routes to enantiomerically enriched 1-butene oxide. *J. Org. Chem.* **52**, 2608-2611 (1987).
15. Ma, B.-D. et al. Increased catalyst productivity in α-hydroxy acids resolution by esterase mutation and substrate modification. *Acs Catal.* **4**, 1026-1031 (2014).
16. Drummond, L.J. & Sutherland, A. Asymmetric synthesis of allylic secondary alcohols: a new general approach for the preparation of α-amino acids. *Tetrahedron* **66**, 5349-5356 (2010).
17. Beresford, K.J.M., Church, N.J. & Young, D.W. Synthesis of α-amino acids by reaction of aziridine-2-carboxylic acids with carbon nucleophiles. *Org. Biomol. Chem.* **4**, 2888-2897 (2006).
18. Qian, C., Gong, L. & Chen, X.Z. Preparation of optical active aliphatic α-amino acid from fatty acid: synthesis of L-norvaline and D-norvaline. *Res. Chem. Intermed.* **35**, 117-121 (2009).
